# LVS Δ*capB*-vectored multiantigenic melioidosis vaccines protect against lethal respiratory *Burkholderia pseudomallei* challenge in highly sensitive BALB/c mice

**DOI:** 10.1128/mbio.00186-24

**Published:** 2024-03-21

**Authors:** Michael V. Tullius, Richard A. Bowen, Peter S. Back, Saša Masleša-Galić, Susana Nava, Marcus A. Horwitz

**Affiliations:** 1 Division of Infectious Diseases, Department of Medicine, Center for Health Sciences, School of Medicine, University of California, Los Angeles, California, USA; 2 Department of Biomedical Sciences, Colorado State University, Fort Collins, Colorado, USA; Fondazione Biotecnopolo di Siena, Siena, Italy

**Keywords:** vaccine, LVS Δ*capB*, *Burkholderia pseudomallei*, melioidosis, select agent, live attenuated vaccine

## Abstract

Melioidosis, caused by the intracellular bacterial pathogen and Tier 1 select agent *Burkholderia pseudomallei* (Bp), is a highly fatal disease endemic in tropical areas. No licensed vaccine against melioidosis exists. In preclinical vaccine studies, demonstrating protection against respiratory infection in the highly sensitive BALB/c mouse has been especially challenging. To address this challenge, we have used a safe yet potent live attenuated platform vector, LVS Δ*capB*, previously used successfully to develop vaccines against the Tier 1 select agents of tularemia, anthrax, and plague, to develop a melioidosis vaccine. We have engineered melioidosis vaccines (rLVS Δ*capB*/Bp) expressing multiple immunoprotective Bp antigens among type VI secretion system proteins Hcp1, Hcp2, and Hcp6, and membrane protein LolC. Administered intradermally, rLVS Δ*capB*/Bp vaccines strongly protect highly sensitive BALB/c mice against lethal respiratory Bp challenge, but protection is overwhelmed at very high challenge doses. In contrast, administered intranasally, rLVS Δ*capB*/Bp vaccines remain strongly protective against even very high challenge doses. Under some conditions, the LVS Δ*capB* vector itself provides significant protection against Bp challenge, and consistent with this, both the vector and vaccines induce humoral immune responses to Bp antigens. Three-antigen vaccines expressing Hcp6-Hcp1-Hcp2 or Hcp6-Hcp1-LolC are among the most potent and provide long-term protection and protection even with a single intranasal immunization. Protection via the intranasal route was either comparable to or statistically significantly better than the single-deletional Bp mutant Bp82, which served as a positive control. Thus, rLVS Δ*capB*/Bp vaccines are exceptionally promising safe and potent melioidosis vaccines.

**IMPORTANCE:**

Melioidosis, a major neglected disease caused by the intracellular bacterial pathogen *Burkholderia pseudomallei*, is endemic in many tropical areas of the world and causes an estimated 165,000 cases and 89,000 deaths in humans annually. Moreover, *B. pseudomallei* is categorized as a Tier 1 select agent of bioterrorism, largely because inhalation of low doses can cause rapidly fatal pneumonia. No licensed vaccine is available to prevent melioidosis. Here, we describe a safe and potent melioidosis vaccine that protects against lethal respiratory challenge with *B. pseudomallei* in a highly sensitive small animal model—even a single immunization is highly protective, and the vaccine gives long-term protection. The vaccine utilizes a highly attenuated replicating intracellular bacterium as a vector to express multiple key proteins of *B. pseudomallei*; this vector platform has previously been used successfully to develop potent vaccines against other Tier 1 select agent diseases including tularemia, anthrax, and plague.

## INTRODUCTION

Melioidosis, a disease endemic in many tropical areas of the world, with ~165,000 cases and 89,000 deaths per year (1, 2), is caused by the intracellular bacterial pathogen *Burkholderia pseudomallei* (Bp). Infection with Bp occurs via inhalation, ingestion, and entry through broken skin (1, 3). Most natural disease is thought to occur via percutaneous inoculation with contaminated soil or water and also via inhalation (3, 4). Melioidosis can present as an acute infection (85% of cases); a chronic infection with symptoms lasting >2 months (11% of cases); or re-activation from latency (4% of cases) (5). Even in a high-resource setting, mortality from naturally acquired melioidosis is ~10%, and where resources are more limited, mortality is ~40%. For acute pneumonia with septic shock, mortality is very high (up to 90%). Prolonged treatment is required—a minimum of 10–14 days of intravenous antibiotics, followed by 3–6 months of oral antibiotics to prevent relapse (5). In addition to its significant public health burden, Bp is categorized as a Tier 1 select agent by CDC. Bp is easily aerosolized and, given the high mortality of pneumonic melioidosis, transmission via inhalation is the route of greatest concern in a bioterrorist attack; inhalation of even low doses of Bp is rapidly fatal in animal models.

In view of Bp’s significant public health burden and potential for weaponization, a potent vaccine against Bp is needed, but currently there are no licensed vaccines. Our approach to developing a safe, effective Bp vaccine that provides long-lasting immunity, is to use a live attenuated intracellular bacterial vector, LVS Δ*capB*, to express Bp antigens. LVS Δ*capB* is derived from Live Vaccine Strain or LVS, a tularemia vaccine developed in the early 1900s that has been administered to ~60 million people including ~5,000 U,S, laboratory workers. LVS is derived from *Francisella tularensis* subsp. *holarctica*, a less virulent subspecies of *F. tularensis* than the Tier 1 select agent *F. tularensis* subsp. *tularensis*. Attenuated by serial passage on artificial medium, LVS has two major attenuating deletions and several minor ones, and yet retains significant toxicity. LVS Δ*capB*, with a third major attenuating deletion resulting from knockout of the *capB* gene, has minimal toxicity; it is >10,000-fold less virulent than LVS when administered to highly sensitive mice intranasally (6). Despite its low virulence, LVS Δ*capB* retains the capacity to invade and multiply in host macrophages among other antigen-presenting cells (6). Consequently, in addition to safety, a major advantage of the LVS Δ*capB* vector is its ability to induce both potent humoral and broad T-cell (CD4+ and CD8+) responses to expressed antigens (7–11).

Utilizing the LVS Δ*capB* vector platform to express immunoprotective antigens of target pathogens, our laboratory has developed potent vaccines against tularemia, anthrax, plague, and COVID-19 (7–11). In the current study, to construct vaccines against melioidosis, we selected four promising Bp antigens—Hcp1, Hcp2, Hcp6, and LolC—for expression in LVS Δ*capB*; these antigens were selected based upon their (i) documented protective capacity as subunit vaccines in mouse studies (12–14); (ii) capacity to generate an immune response in melioidosis patients (Hcp1 and LolC) (12, 15, 16); and (iii) high sequence conservation in Bp strains. We generated rLVS Δ*capB*/Bp vaccines expressing individual Bp antigens as well as fusion proteins comprising 2, 3, or 4 Bp antigens. We adapted the Electra cloning system (17) to facilitate the construction of a large number of fusion protein variants so that we could optimize for heterologous expression of the antigens by the LVS Δ*capB* vector. We varied the coding regions (native vs codon-optimized), the order of the coding regions, and the linkers fusing the coding regions in order to identify the best-expressed fusion proteins. By doing so, we achieved good expression of fusion proteins consisting of two or three antigens, with molecular weights of 42–67 kDa; however, the expression of fusion proteins comprising all four antigens (~87 kDa) was relatively poor.

We assessed the efficacy of our rLVS Δ*capB*/Bp vaccines in BALB/c mice, a strain of mice especially sensitive to Bp (18–20). Moreover, we challenged these mice with lethal doses of highly virulent Bp via the respiratory (intranasal) route, the most difficult route to protect against. This challenge model set a high bar for our vaccines, as very few vaccines have demonstrated significant efficacy against lethal respiratory challenge in BALB/c mice (21). In a series of six independent experiments, we demonstrate that rLVS Δ*capB*/Bp vaccines can induce high levels of protective immunity against lethal respiratory challenge in BALB/c mice when the vaccines are administered intradermally or intranasally. When mice were immunized by the intradermal (ID) route, the protective efficacy of the rLVS Δ*capB*/Bp vaccines was overwhelmed at high challenge doses, but when mice were immunized by the intranasal (IN) route, protection remained strong even at high challenge doses. Intranasal vaccination resulted in protective efficacy greater than the positive control vaccine, Bp82, a live attenuated Bp strain with a single major attenuating deletion (22) and hence unsuitable for clinical use, due to safety concerns regarding reversion to virulence of a vaccine comprising an attenuated bacterial pathogen when only one major attenuating mutation is present (23). While most of our studies utilized a homologous prime-boost vaccination strategy (three doses, 4 weeks apart), even a single intranasal immunization was highly protective. Surprisingly, the LVS Δ*capB* vector by itself was sometimes capable of eliciting potent protection, particularly when delivered by the intranasal route. rLVS Δ*capB*/Bp vaccines expressing three-antigen fusion proteins Hcp6-Hcp1-Hcp2 or Hcp6-Hcp1-LolC were among the most potent vaccines tested.

## MATERIALS AND METHODS

### Bacterial strains and media

The bacterial strains used in this study are listed in Table S1. *Escherichia coli* was grown on Luria-Bertani or YT agar and Luria-Bertani broth at 37°C. Ampicillin (100 µg/mL) and kanamycin (30 or 50 µg/mL) were included as appropriate. Bp82 was grown on Luria-Bertani (Lennox) agar plates containing 0.6 mM adenine at 37°C. LVS Δ*capB* and rLVS Δ*capB*/Bp strains were grown on chocolate agar plates (CA; Difco GC Medium Base + 1% [wt/vol] bovine hemoglobin + 1% [vol/vol] IsoVitaleX enrichment) and modified medium T broth (24, 25) at 37°C (we substituted N-Z-Amine A [enzymatic digest of casein] for the Casamino Acids [acid hydrolysate of casein] component of the formula). Kanamycin (7.5 µg/mL) was included for rLVS Δ*capB*/Bp strains maintaining pFNL plasmids. Bp 1026b was grown on BHI plates and colonies were harvested in BHI broth 24 h after plating. The Bp 1026b suspension was vortexed to a uniform appearance, glycerol was added with mixing to 15%, and 0.5 mL aliquots were frozen rapidly and stored at −80°C.

### Construction of Electra compatible *E. coli-Francisella* shuttle plasmids

To facilitate cloning and expression analysis of Bp antigens (individually or as fusion proteins), we first constructed three Electra compatible DAUGHTER plasmids. Electra cloning (17) is similar to fragment exchange (FX) cloning (26) and uses SapI, a type IIS restriction enzyme that produces 3 bp overhangs, together with T4 DNA ligase in a single-tube reaction.

To generate Electra compatible DAUGHTER plasmids, we first removed the four SapI restriction sites from the *E. coli-Francisella* shuttle expression vector pFNL/pbfr-SD-iglA (10), using the QuikChange Lightning Multi Site-Directed Mutagenesis Kit (Agilent; Santa Clara, CA). We used PrimerX software (27) to design four mutagenesis primers and, as all four SapI restriction sites occurred within open reading frames (ORFs), we ensured that the mutations did not affect the protein sequence from the mutated ORF’s translation (see Table S2 for primers). Next, we replaced the *iglA* coding sequence with three different Electra compatible *sacB* cassettes, to generate three Electra compatible DAUGHTER plasmids, pFNL-bfr-D1 (sacB), pFNL-bfr-D2[N3F-8H] (sacB), and pFNL-bfr-D3[C8H-3F] (sacB) (Tables S3 and S4). Using Electra cloning, ORFs from Electra compatible MOTHER plasmids can be readily cloned in frame into the three different DAUGHTER vectors, with expression of the ORF driven from the strong *Francisella* bacterioferritin promoter. Cloning into the D1 vector results in no fusion with the ORF, cloning into the D2 vector results in an N-terminal fusion of a dual 3×FLAG-His_8_ tag (MDYKDHDGDYKDHDIDYKDDDDKHHHHHHHHGGGS) with the ORF, and cloning into the D3 vector results in a C-terminal fusion of the ORF with a dual His_8_-3×FLAG tag (GGSHHHHHHHHDYKDHDGDYKDHDIDYKDDDDK). ORFs excised from Electra MOTHER plasmids with SapI have a 5′ overhang of ATG on the coding strand (coding for Met) and an ACC overhang on the complementary strand (compatible with the GGT overhang of the vector, coding for Gly). Thus, ORFs cloned into the D1 or D2 DAUGHTER vectors, in most cases, will have an extra Gly residue on the C-terminus of the protein, unless the native sequence ends in a Gly residue. The presence of the *sacB* cassette in these vectors (which is replaced by the desired ORF in the cloning reaction), permitted us to use undigested DAUGHTER vectors in Electra reactions instead of gel-purified, linearized vector. Selection of transformants on agar plates with kanamycin and 5% sucrose was highly successful in selecting for recombinant DAUGHTER plasmids as transformants that take up unmodified DAUGHTER plasmids are killed in the presence of sucrose due to expression of the *sacB* gene.

We used *in vivo* assembly (IVA) (28) to construct several additional Electra compatible DAUGHTER plasmids derived from the original three plasmids. We first created a new series of Electra plasmids, in which the unnecessary ampicillin resistance gene is removed, by amplifying the original Electra plasmids using primers pFNL-delAmp-F and pFNL-delAmp-R and transforming the polymerase chain reaction (PCR) products into *E. coli* to generate pFNLdA-bfr-D1 (sacB), pFNLdA-bfr-D2[N3F-8H] (sacB), and pFNLdA-bfr-D3[C8H-3F] (sacB). We also used IVA to construct additional derivatives of these new pFNLdA DAUGHTER plasmids with modified SapI overhangs to allow for cloning of ORFs from MOTHER plasmids which have different SapI overhangs (see further details below). Derivatives with a modified SapI overhang at the 5′ end of the cloning site (GCA or TCT, instead of ATG) have an ATG codon immediately preceding the SapI overhang for translation of the cloned ORF.

Recombinant plasmids were confirmed to be correct by restriction analysis and DNA sequencing.

### Electra cloning to construct expression plasmids with a single Bp antigen ORF

We purchased two Electra MOTHER plasmids from DNA2.0 (now ATUM; Newark, CA) containing an ORF encoding the Bp Hcp6 antigen (*hcp6*, BPSL3105), with one version codon-optimized for *Francisella tularensis* subsp. *holarctica* LVS (http://www.kazusa.or.jp/codon/cgi-bin/showcodon.cgi?species=376619&aa=1&style=GCG), and one version codon-optimized for *Listeria monocytogenes* (http://www.kazusa.or.jp/codon/cgi-bin/showcodon.cgi?species=1639&aa=1&style=GCG), cloned into pM268 and pM264 MOTHER plasmids, respectively.

For construction of additional Electra MOTHER plasmids containing ORFs for other Bp antigens (*hcp1* [BPSS1498, *hcp2* [BPSS0518], and *lolC* [BPSL2277]), we first generated plasmid pM264-sacB, a derivative of pM264 with a *sacB* cassette inserted in between the SapI restriction sites (Tables S2 and S5). This allowed us to use undigested pM264-sacB, rather than gel-purified, linearized vector, in Electra reactions to clone PCR products and synthetic DNA fragments. Transformation of Electra reactions into *E. coli* and plating on agar plates with ampicillin and 5% sucrose was highly successful in selecting for recombinant MOTHER plasmids, as transformants that take up residual pM264-sacB are killed in the presence of sucrose due to expression of the *sacB* gene.

For *hcp2*, *hcp6*, and *lolC*, we used GeneDesigner 2.0 software (DNA2.0/ATUM) to generate coding sequences codon-optimized for LVS and *L. monocytogenes*, purchased Electra compatible synthetic DNA fragments for these codon-optimized genes from SGI-DNA (San Diego, CA), and cloned them into the pM264-sacB. In the case of *lolC*, we only cloned the sequence encoding the periplasmic region (amino acid residues 51–273) (13). For *hcp1*, *hcp2*, and *hcp6*, we also amplified the native genes from Bp K96243 gDNA (gift from Christopher T. French) and cloned the PCR products into pM264-sacB. We were unsuccessful in amplifying the native Bp *lolC*.

For a typical cloning reaction, we would mix a MOTHER plasmid containing a Bp antigen ORF (Table S6), a pFNL DAUGHTER plasmid (Table S3), and Electra Cloning reagents (DNA2.0/ATUM) containing buffer, SapI, and T4 DNA ligase in a final volume of 5 µL and incubate at room temperature for 1 h. We would then use 1 µL of the cloning reaction to transform 10 µL of competent *E. coli* and select for recombinant clones on YT plates containing kanamycin and 5% sucrose.

Recombinant plasmids were confirmed to be correct by restriction analysis and DNA sequencing.

### Electra cloning to construct expression plasmids with multiple Bp antigens joined as a fusion protein

For our first set of fusion constructs, we joined *hcp6* or *lolC* to either *hcp1* or *hcp2* with a sequence coding for a flexible peptide linker (GSSG-GSSG) in between the two ORFs. To accomplish this, we first amplified *hcp6* and *lolC* by PCR, using a primer for the pM264 vector (pM264-FP) and a primer specific for the gene (hcp6_coLm-L-R, lolC_coLm-L-R, or lolC_coLVS-L-R) which included the sequence for the peptide linker in the primer tail. The PCR products were reamplified in a second PCR using primers pM264-FP and Linker-3gca-R, which allowed the resulting PCR products to be cloned by Gibson Assembly (29) into the SapI cloning sites of the pM264 MOTHER vector, replacing the 3′GGT SapI overhang with a GCA overhang. We then amplified *hcp1* and *hcp2* by PCR, using a primer specific for the gene (hcp1_nat-5gca-F, hcp1_coLm-5gca-F, hcp1_coLVS-5gca-F, hcp2_nat-5gca-F, hcp2_coLm-5gca-F, or hcp2_coLVS-5gca-F) and a primer specific for the pM264 vector (pM264-RP). The gene-specific primer included 30–33 nt of homology to the pM264 vector allowing the PCR product to be cloned by Gibson Assembly into the pM264 SapI cloning sites, replacing the 5′ATG SapI overhang with a GCA overhang. Fusions of two ORFs (*hcp6* plus *hcp1* or *hcp2*; and *lolC* plus *hcp1* or *hcp2*) were done by combining pM264 MOTHER plasmids with compatible GCA overhangs (Table S6) together with a pFNL DAUGHTER plasmid (Table S3) using Electra cloning to generate the desired expression plasmid (Table S7). For example:


$$\begin{array}{l} pFNL\;-\;bfr\;-\;D3[C8H\;-\;3F](sacB)+\\ \;\;\;pM264(3^{\prime }gca)\;-\;Bp\;hcp6\;(coLm)\;-\;(GSSG)2+pM264(5^{\prime }gca)\;-\;Bp\;hcp1(coLVS)\to \\ \;\;\;\;\;pFNL\;-\;bfr\;-\;Hcp6\;(coLm)\;-\;(GSSG)2\;-\;Hcp1(coLVS)\;-\;C8H\;-\;3F \end{array}$$

To facilitate the construction of subsequent fusion proteins, we made additional modifications to the Electra cloning system to simplify the in-frame joining of two or three ORFs. Using Gibson assembly or IVA (primer pairs listed in Table S2), we constructed five modified pM264-sacB MOTHER plasmids which vary in the SapI overhangs (Table S5), with the 5′ ATG overhang on the coding strand (coding for Met) and/or the GGT overhang for the 3′ end of the ORF (coding for Gly) being replaced by GCA (Ala) or TCT (Ser). We then cloned ORFs into the modified pM264-sacB plasmids. Finally, we performed Electra reactions combining two or three modified MOTHER plasmids (each with an ORF) and a DAUGHTER plasmid to construct expression plasmids with fusion protein genes. Fusions of two ORFs were done by combining pM264 MOTHER plasmids with compatible GCA or TCT overhangs (see GCA example above). Likewise, fusions of three ORFs were done by combining three MOTHER plasmids with compatible GCA and TCT overhangs together with a pFNL/pFNLdA DAUGHTER. For example:


$$\begin{array}{l} pFNLdA\;-\;bfr\;-\;D1(sacB)+\\ \;\;\;\;\;pM264(3^{\prime }gca)\;-\;ORF1+pM264(5^{\prime }gca,3^{\prime }tct)\;-\;ORF2+pM264(5^{\prime }tct)\;-ORF3\to \\ \;\;\;\;\;pFNLdA-bfr-ORF1-\;GCA\;(Ala)\;-ORF2\;-\;TCT\;(Ser)\;-\;ORF3 \end{array}$$

To enable joining two ORFs with a peptide linker, we constructed three separate linkers, a flexible linker (GSAGSAAGSGEF) (30) and two rigid linkers [A(EAAAK)_3_A and (AP)_10_] (31, 32), by annealing complementary oligonucleotides with the appropriate 3 bp overhangs for cloning into pM264(5′gca, 3′tct)-sacB (Tables S2, S4, and S6). We also constructed a series of modified pFNLdA plasmids using IVA (Table S3) that allows for direct cloning of inserts from modified pM264-sacB MOTHER plasmids (i.e. the modified insert can be cloned by itself, without fusion to one or two other inserts), such that individual components of the desired fusion protein can be tested. For example:


$$\begin{array}{l} pFNLdA\;-\;bfr\;-\;D31(5^{\prime }gca)[C8H\;-\;3F]\;-\;sacB+pM264(5^{\prime }gca)\;-\;ORF2\to \\ \;\;\;pFNLdA\;-\;bfr\;-\;ORF2\;-\;C8H\;-\;3F \end{array}$$

For expression of four-antigen fusion proteins, we first attempted to combine two of the two-antigen constructs that were expressed well in LVS Δ*capB*, Hcp6-(GSSG)2-Hcp1 and LolC-(GSSG)2-Hcp2. To achieve this, we amplified the Hcp6-(GSSG)2-Hcp1 ORF from the pFNL-bfr-Hcp6 (coLm)-(GSSG)2-Hcp1 (coLVS)-C8H-3F expression plasmid (Table S7) using primers hcp6_coLm-(ATG)-F and hcp1_coLVS-(GCA)-R (Table S2) and cloned the PCR product into pM264(3′gca)-sacB, generating pM264(3′gca)-Hcp6 (coLm)-(GSSG)2-Hcp1 (coLVS) (Table S6). Likewise, we amplified the Hcp6-(GSSG)2-Hcp1 ORF using primers hcp6_coLm-(ATG)-F and hcp1_coLVS-(TCT)-R and cloned the PCR product into pM264(3′tct)-sacB, generating pM264(3′tct)-Hcp6 (coLm)-(GSSG)2-Hcp1 (coLVS). The final required MOTHER plasmid, pM264(5′tct)-LolC (coLVS)-(GSSG)2-Hcp2 (coLVS) was constructed by cloning a PCR product of the LolC-(GSSG)2-Hcp2 ORF [using primers lolC_coLVS-(TCT)-F and hcp2_coLVS-(GGT)-R] into pM264(5′tct)-sacB. We then performed Electra reactions combining two MOTHER plasmids (each with a two-antigen ORF) and a DAUGHTER plasmid, with or without one of the three MOTHER plasmids containing a peptide linker ORF, to construct expression plasmids with four-antigen fusion protein genes as detailed above. As these constructs were very poorly expressed by LVS Δ*capB*, we next turned to expressing three-antigen fusion proteins.

For the expression of three-antigen fusion proteins, we took a systematic approach, linking LolC to either the N-terminus or C-terminus of the most highly expressed two-antigen ORFs [Hcp6-(GSSG)2-Hcp1 and Hcp6-(GSSG)2-Hcp2]. For each combination of antigens, the C-terminal portion was linked to the N-terminal portion by one of three peptide linkers or directly (only a single alanine inserted between the two), for a total of 16 three-antigen fusion protein variations, using the same cloning approach as detailed above for the four-antigen constructs (Table S8).

### Construction and characterization of rLVS Δ*capB*/Bp strains

Expression plasmids were introduced into LVS Δ*capB* by electroporation or chemical transformation. To prepare electrocompetent cells, we grew LVS Δ*capB* overnight in medium T at 37°C with shaking, subcultured in medium T to an OD_600_ of 0.1 or 0.2, and cultured for an additional 4 or 5 h, reaching an OD_600_ of 0.7–1.1. The bacteria were harvested by centrifugation at 4°C, washed two or three times with ice-cold sucrose-glycerol wash buffer (SGWB; 10% [vol/vol] glycerol, 500 mM sucrose; pH ~7) (33), resuspended in a final volume of SGWB equivalent to 1/50th to 1/10th of the original culture volume, and aliquots stored at −80°C until needed. For electroporation, 1–2 µL of plasmid DNA was mixed with 30–50 µL of electrocompetent cells on ice and transferred to a pre-chilled electroporation cuvette (0.1 cm, Bio-Rad). After the pulse was applied (1.8 kV, 25 µF, and 200 Ω), we resuspended electroporated bacteria in 0.5 mL medium T, incubated at 37°C for 3–5 h, and then plated on CA containing 7.5 µg/mL kanamycin. Typically, individual clones were picked for analysis after 3–5 days of incubation at 37°C.

To prepare chemically competent cells, we grew LVS Δ*capB* overnight in medium T at 37°C with shaking, subcultured in medium T to an OD_600_ of 0.1, and cultured for an additional 4 h, reaching an OD_600_ of 0.5. Competent cells were prepared using the method that Inoue et al. described for *E. coli* (34), except that we grew LVS Δ*capB* at 37°C instead of a reduced temperature. Aliquots of competent cells were stored at −80°C until needed. For transformation of LVS Δ*capB*, we modified a typical protocol we use for small-scale *E. coli* transformations. We mixed 1 µL of plasmid DNA with 20 µL of competent cells in a PCR tube on ice; incubated on ice for 30 min; applied a 42°C heat shock for 45 s in a thermal cycler machine and cooled to 4°C; incubated on ice for 2 min; added recovery medium (130 µL medium T) and mixed gently; incubated at 37°C for 3 h (no shaking); and plated the entire transformation on CA containing 7.5 µg/mL kanamycin. Typically, individual clones were picked for analysis after 3–5 days of incubation at 37°C.

Individual clones were picked and suspended into 2 mL medium T broth containing 7.5 µg/mL kanamycin and grown overnight at 37°C with shaking. To analyze recombinant protein expression, we prepared cell lysates from 1 mL of overnight culture by pelleting the cells by centrifugation; freezing the cell pellet at −30°C for at least 1 h; resuspending the thawed cell pellet in 0.1 mL of Complete Bacterial Protein Extraction Reagent (B-PER; Thermo Scientific) containing 5 mM EDTA and a protease inhibitor cocktail (HALT Protease Inhibitor Single Use Cocktail, Thermo Scientific); and incubating at room temperature for 10–20 min, at which point the bacterial suspensions had cleared. After centrifuging to clear any remaining cellular debris, we mixed 20 µL of supernatant with 20 µL of 2× SDS-PAGE sample buffer and boiled for 5 min. Boiled lysates were analyzed on Any kD Mini-PROTEAN TGX Stain-Free Protein Gels (Bio-Rad) and total protein was visualized by UV light and/or by Coomassie Blue staining. Recombinant proteins with a 3×FLAG tag were detected by Western blotting using horseradish peroxidase (HRP) conjugated anti-FLAG monoclonal antibody (100,000-fold dilution, Sigma) and Clarity Western ECL Substrate (Bio-Rad). Hcp1 was detected by Western blotting using rat anti-Hcp1 antibody (5,000-fold dilution, generously provided by Christopher T. French) and goat anti-rat HRP (10,000-fold dilution, Invitrogen).

### Preparation of vaccine stocks

Bp82 was grown overnight at 37°C on Luria-Bertani (Lennox) agar plates containing 0.6 mM adenine. Bacterial colonies were scraped from the agar plates into phosphate-buffered saline (PBS) containing 15% (vol/vol) glycerol and clumps dispersed by vortexing and pipetting. Large aggregates were allowed to settle for 10 min and then the upper portion of the suspension (avoiding any pellet) was aliquoted and stored at −80°C until needed.

LVS Δ*capB* and rLVS Δ*capB*/Bp vaccines expressing Bp antigens were grown from a frozen stock overnight at 37°C with shaking in medium T broth (+ 7.5 µg/mL kanamycin for strains with a pFNL plasmid), subcultured in medium T to an OD_600_ of 0.001–0.003, and grown for 18–20 h, reaching an OD_600_ of 0.6–3.8 (equivalent to 8.4–10.4 generations of growth, doubling time: 1.7–2.1 h, median = 1.9 h, *N* = 29). The bacteria were harvested by centrifugation at 4°C, washed two times with PBS, resuspended in a final volume of PBS-20% (vol/vol) glycerol equivalent to 1/10 of the original culture volume, and aliquots stored at −80°C until needed. We checked the vaccine stocks for the stability of the plasmid and antigen expression as follows. Dilutions of a thawed vaccine stock were first plated on CA to determine the post-freeze titer. Twenty individual clones were then patched onto CA plates with and without kanamycin to determine stability of the pFNL plasmid. To validate that the correct plasmids were present in the rLVS Δ*capB*/Bp vaccines, we isolated plasmid DNA from thawed vaccine stocks using the Zyppy Plasmid Miniprep Kit (Zymo Research; Irvine, CA) and confirmed it to be correct by restriction analysis, as well as by DNA sequencing of the expression cassette and/or PCR of the expression cassette, followed by DNA sequencing of the PCR product. Finally, vaccine stocks were used to inoculate Medium T broth containing 7.5 µg/mL kanamycin, grown overnight, and lysates prepared to check recombinant protein expression (as described above).

### Mice

For protection studies at Colorado State University (CSU), 6- to 8-week-old female BALB/c mice were purchased from Charles River or Envigo, held four per cage, and provided food and water *ad libitum*. Mice were acclimated for 1 week prior to initiating the experiments.

For immunology studies at University of California, Los Angeles (UCLA), 6- to 8-week-old BALB/c mice (half male and half female) were purchased from The Jackson Laboratory, held four per cage, and provided food and water *ad libitum*. Mice were acclimated for 1 week prior to initiating the experiments.

### Protective efficacy and immunology studies

For rLVS Δ*capB*/Bp vaccines, we immunized mice by either the ID route at the base of the tail (1, 2, 4, or 8 × 10^6^ CFU) or the IN route (2 × 10^6^ CFU) using a homologous boosting regimen administered at weeks 0, 4, and 8. In the case of IN vaccination, additional groups of mice received only one dose (week 8) or two doses (weeks 4 and 8) of vaccine instead of the usual three doses (constant immunization-challenge interval). Mice immunized by the IN route were anesthetized with ketamine/xylazine and administered 20 µL of vaccine divided between both nostrils. Each protective efficacy experiment included three control groups: a negative control group (sham-immunized), a positive control group (immunized with 1 × 10^6^ CFU Bp82 by the ID route), and the parental vector (LVS Δ*capB*).

In protective efficacy studies, we collected blood 1 week prior to challenge to prepare serum, which was stored at −80°C until analysis for antibody to Bp and LVS antigens. Four, 6, or 12 weeks after the last vaccine dose, mice were challenged by the IN route with a lethal dose of Bp 1026b (1,430–7,800 CFU) and monitored for survival for 6 weeks. Mice were euthanized when they reached humane endpoints. At the end of the 6-week period for monitoring survival, the surviving mice were euthanized, and the lung, liver, and spleen were examined for abscesses and then homogenates cultured for Bp: 10 mg of each organ were plated representing approximately 13% of the spleen, 1% of the liver, and 7% of the lung. Mice with no abscesses and no detectable Bp in the three organs were recorded as having sterile immunity.

For immunology studies, 1 week after the last vaccine dose, we euthanized mice and removed the spleen and lungs to assess immune responses.

### Expression and purification of recombinant Bp antigens

We used the Expresso Rhamnose SUMO Cloning and Expression System (Lucigen) to obtain purified recombinant Bp antigens for immunology assays. ORFs for *hcp1*, *hcp2*, *hcp6*, and *lolC* (codon-optimized for LVS) were amplified from pM264/pM268 plasmids and cloned into the pRham N-His SUMO Kan expression vector (Tables S2 and S9). The coding regions were amplified (starting with the second codon) with tails recommended by Lucigen for cloning into their linearized vector (tails have 18 nt of homology to the vector to allow for IVA) such that the Bp genes will be fused downstream of a His_6_-SUMO tag under the control of the L-rhamnose-inducible rhaP_BAD_ promoter. The second codon for both *hcp1* and *hcp6* codes for leucine, which is inefficiently cleaved by SUMO protease. Therefore, we inserted codons for Gly-Ser in front of the Leu codon for these two genes to facilitate cleavage of the SUMO tag. Purified PCR products and linearized pRham N-His SUMO Kan expression vector were mixed together, transformed into competent *E. coli* (E. cloni 10G, Lucigen), and clones selected on YT containing 30 µg/mL kanamycin. We confirmed that the recombinant plasmids were correct by restriction analysis and DNA sequencing and confirmed that we could obtain high level, inducible expression of the N-His-SUMO tagged recombinant proteins before proceeding with purification.

To induce fusion protein expression, we used an auto-induction protocol, inoculating 10 mL of an overnight culture into 1 L LB with 0.05% glucose, 0.05% rhamnose, and 30 µg/mL kanamycin in a 2.8-L Fernbach flask, and incubating at 28°C for 24 h with shaking (reaching an OD_600_ of ~5). The bacteria were harvested by centrifugation at 4°C, washed first with 50 mL ice-cold 2× TE pH 8.0, followed by 25 mL ice-cold ddH_2_O, and the washed cell pellets were stored at −80°C until needed. Recombinant proteins were extracted by resuspending thawed cell pellets in 50 mL B-PER Complete Bacterial Protein Extraction Reagent (Thermo Scientific) and incubating at room temperature for 60 min. The extract was then clarified by centrifugation at 10,000 × *g* for 60 min at 4°C and the supernatant was filtered (0.2 µm). Filtered extract was mixed with 2 mL of HisPur Cobalt Superflow Agarose resin (Thermo Scientific) and rocked gently at 4°C overnight for binding of the His-SUMO-tagged recombinant protein to the resin. After the column was extensively washed, we eluted recombinant protein with 150 mM imidazole in 50 mM NaH_2_PO_4_-300 mM NaCl buffer (pH 7.4). We performed concentration and buffer exchange of the 150 mM imidazole elution fractions using an Amicon Ultra-15 centrifugal filter device (10,000 MWCO) (Millipore) into 20 mM Tris-150 mM NaCl-10% (vol/vol) glycerol pH 8.0, added DTT to a final concentration of 2 mM, and digested with SUMO Express Protease (Lucigen) overnight at 4°C at a concentration of ~1 U per 2 mg of His-SUMO-tagged recombinant protein. The digests (<0.5 mL) were diluted to 20 mL PBS and passed through a HisPur Cobalt column for subtractive chromatography to remove the cleaved His_6_-SUMO fragment, the SUMO Express Protease (His tagged), and *E. coli* protein contaminants that bound to the column during the original binding step. The flow through was concentrated and the buffer was exchanged into PBS using an Amicon Ultra-15 centrifugal filter device (10,000 MWCO), and then filtered to sterilize with a 0.2-µm Spin-X column (Corning). We measured the protein concentration of the purified proteins using the protein’s extinction coefficient at 280 nm (calculated using the ProtParam tool, https://web.expasy.org/protparam/) (35) and assessed purity by SDS-PAGE. Aliquots were stored at −80°C until needed.

### Other antigens

A peptide pool for Hcp2 (41 individual 15 amino acid peptides with an 11 amino acid overlap between adjacent peptides, >70% purity) was purchased from JPT Peptide Technologies (Berlin, Germany). To prepare heat-inactivated Bp82 and LVS Δ*capB*, we scraped bacteria from agar plates, washed with PBS, incubated at 80°C for 30 min or 1 h (respectively) to kill the bacteria, and stored in aliquots at −80°C.

### Isolation of splenocytes and lung cells

One week after the last vaccine dose, we euthanized mice and removed the spleen and lungs to assess immune responses. Single-cell suspensions of splenocytes were prepared by gently pressing the cells out of the spleen sac; lysing red blood cells with PharmLyse (BD Pharmingen); washing the cells; and filtering through a 70 µm nylon cell strainer (Falcon). Single-cell suspensions of lung cells were prepared by cutting the lung into small pieces with a scalpel; incubating at 37°C for 1 h with shaking in 10 mL of digestion solution (300 U/mL collagenase type II [Worthington] and 0.15 mg/mL DNase I [Worthington] in PBS); filtering through a 40-µm nylon cell strainer (Falcon); lysing red blood cells with PharmLyse (BD Pharmingen); and washing the cells. Advanced RPMI-1640 (Invitrogen) supplemented with 2% heat-inactivated fetal bovine serum, 2 mM glutamine dipeptide (glutaGRO Supplement, Corning), 10 mM HEPES buffer, 50 µM β-mercaptoethanol, and penicillin (100 IU/mL)-streptomycin (100 µg/mL) was used as the medium.

### Flow cytometry analysis

Single-cell suspensions of splenocytes (5 × 10^5^ viable cells per well) and lung cells (2–3 × 10^5^ viable cells per well) were stimulated with individual antigens or left without antigen for 6 h in U-bottom, 96-well tissue culture plates in 200 µL medium at 37°C in a humidified incubator (95% air, 5% CO_2_). For antigen stimulation, we used rHcp1, rHcp6, and rLolC each at a final concentration of 10 µg/mL and the Hcp2 peptide pool at a final concentration of 1 µg/mL for each peptide (41 µg/mL for the total pool). Heat-inactivated Bp82 and LVS Δ*capB* were used at 5 × 10^6^ CFU per well (CFU assayed prior to heat inactivation). Anti-CD28 antibody (Clone 37.51) was included in all wells as a co-stimulant at 2 µg/mL. The protein transport inhibitor brefeldin A (5 µg/mL final concentration) was added to all wells for the final 4 h of incubation. At the end of the 6-h incubation, we performed viability staining using Fixable Viability Dye eFluor 780 (eBioscience); fixed and permeabilized the cells using the Cyto-Fast Fix/Perm Buffer Set (BioLegend); blocked Fc Receptors with anti-mouse CD16/32 antibody (TruStain FcX PLUS, BioLegend); and stained intracellular and surface antigens using fluorescent antibodies for CD3, CD4, CD8, IFNγ, TNFα, IL-2, IL-17A, Perforin, and Granzyme B (Table S10). A minimum of 16,000 live CD3+ T cells per spleen sample (median, ~29,000) and a minimum of 2,500 live CD3+ T cells per lung sample (median, ~5,000 in the first experiment and ~9,000 in the second experiment) were acquired with a BD LSRII flow cytometer equipped with a high throughput sampler and five lasers (355, 405, 488, 561, and 640 nm). The frequencies of live CD3+CD4+ and CD3+ CD8+ T cells expressing IFNγ, IL-2, TNFα, IL-17, Granzyme B, and Perforin were determined using FlowJo (FlowJo; Ashland, OR) and FCS Express software (*De Novo* Software; Pasadena, CA). Background numbers of cells producing cytokines without antigen stimulation were subtracted.

### Serum antibody

Frozen serum (obtained from vaccinated mice 1 week prior to challenge) was thawed at 4°C and assayed for antibody to Bp and LVS Δ*capB* antigens by enzyme-linked immunosorbent assay (ELISA). High Binding enzyme immunoassay (EIA) plates (Corning) were coated with 100 µL of antigen per well in 0.1 M sodium carbonate buffer pH 9.6 (protein antigens: 1 µg/mL; heat-inactivated LVS Δ*capB* or Bp82: 5 × 10^7^ CFU/mL [CFU prior to heat inactivation]) for 4 h at room temperature and then blocked with 3% (wt/vol) bovine serum albumin (BSA) in PBS. The plates were then washed with PBS-0.05% TWEEN 20 before adding 100 µL per well of immune serum diluted in PBS-1% (wt/vol) BSA (we typically prepared a 200-fold dilution, followed by serial 4-fold dilutions out to 204,800-fold dilution of the original serum). Plates were incubated overnight at 4°C with immune sera, washed with PBS-0.05% TWEEN 20, and then the alkaline phosphatase (AP) conjugated secondary antibody was added (100 µL per well at 1:5,000 dilution in PBS-1% [wt/vol] BSA, goat anti-mouse immunoglobulin G [IgG]-AP [Sigma]). After 90 min at room temperature, the plates were washed with TBS-0.05% TWEEN-80, developed with the Alkaline Phosphatase Substrate Kit (Bio-Rad) according to the manufacturer’s instructions, and absorbance at 415 and 750 nm measured with a Bio-Rad iMark microplate reader. We plotted log (A415–A750) vs log serum dilution to visualize the data. We calculated the endpoint titer as the dilution where the measurement intersects the cutoff, using interpolation between data points, and with the cutoff equal to the sham mean + 3 SD, but at least 0.1. In a few cases, sham mice clearly reacted to an antigen, so these mice were excluded for purposes of calculating the endpoint titer cutoff. However, they were not excluded from the final titer results. The Python source code for processing and visualizing the raw ELISA data and calculating endpoint titers is available at https://github.com/mvtullius/serum-antibody-ELISA.

### Statistics

For efficacy studies, we processed survival data with a Python script, comparing groups using pairwise log-rank tests (lifelines package [36]), with Holm-Bonferroni correction of *P* values for multiple comparisons (statsmodels package [37]). The source code is available at https://github.com/mvtullius/Bp-survival-analysis. *P* values for selected comparisons were confirmed using GraphPad Prism 9.3.1. Serum IgG titers were compared to a control group using ordinary one-way ANOVA with Dunnett’s multiple comparisons test (Prism 9.3.1).

## RESULTS

### Construction of Bp vaccines expressing a single Bp antigen based on the LVS Δ*capB* platform

To construct vaccines against melioidosis, we selected four promising antigens from Bp for expression in LVS Δ*capB*: Hcp1 (BPSS1498), Hcp2 (BPSS0518), Hcp6 (BPSL3105), and LolC (BPSL2277). Hcp1, Hcp2, and Hcp6 are surface-associated components from three of the six Type VI secretion systems (T6SSs) present in Bp (12). LolC is a membrane protein involved in lipoprotein sorting in Gram-negative bacteria (13). All four of these proteins have been shown to provide some level of protective efficacy against Bp challenge in mice when administered as a recombinant protein in adjuvant (12–14).

To facilitate the construction and characterization of rLVS Δ*capB*/Bp vaccines expressing Bp antigens, we adapted the *E. coli-Francisella* shuttle expression vector pFNL/pbfr-SD-iglA (10) to construct three Electra compatible DAUGHTER plasmids that allow for ORFs from Electra compatible MOTHER plasmids to be cloned downstream of the strong *Francisella* bacterioferritin promoter with a ribosomal binding site (Tables S3 and S4). The three versions of the expression plasmid allow cloning of ORFs without an additional tag [pFNL-bfr-D1 (sacB)]; with an N-terminal fusion of a dual 3×FLAG-His_8_ tag {pFNL-bfr-D2[N3F-8H] (sacB)}; or with a C-terminal fusion of a dual His_8_-3×FLAG tag {pFNL-bfr-D3[C8H-3F] (sacB)}. Next, we transferred ORFs coding for the four Bp antigens to the three expression plasmids, electroporated the plasmids into LVS Δ*capB*, and analyzed cleared cell lysates from individual clones for expression of the heterologous proteins. To improve our chances of obtaining good expression, we tested two or three versions of each gene: (i) the native gene amplified from Bp K96243 gDNA; (ii) a synthetic gene codon-optimized for LVS (coLVS); and (iii) a synthetic gene codon-optimized for *Listeria monocytogenes* (coLm). The coLm gene was intended for a separate project using attenuated *L. monocytogenes* as a vaccine vector, but as it was available, we tested it in LVS Δ*capB* as well.

**Fig 1:**
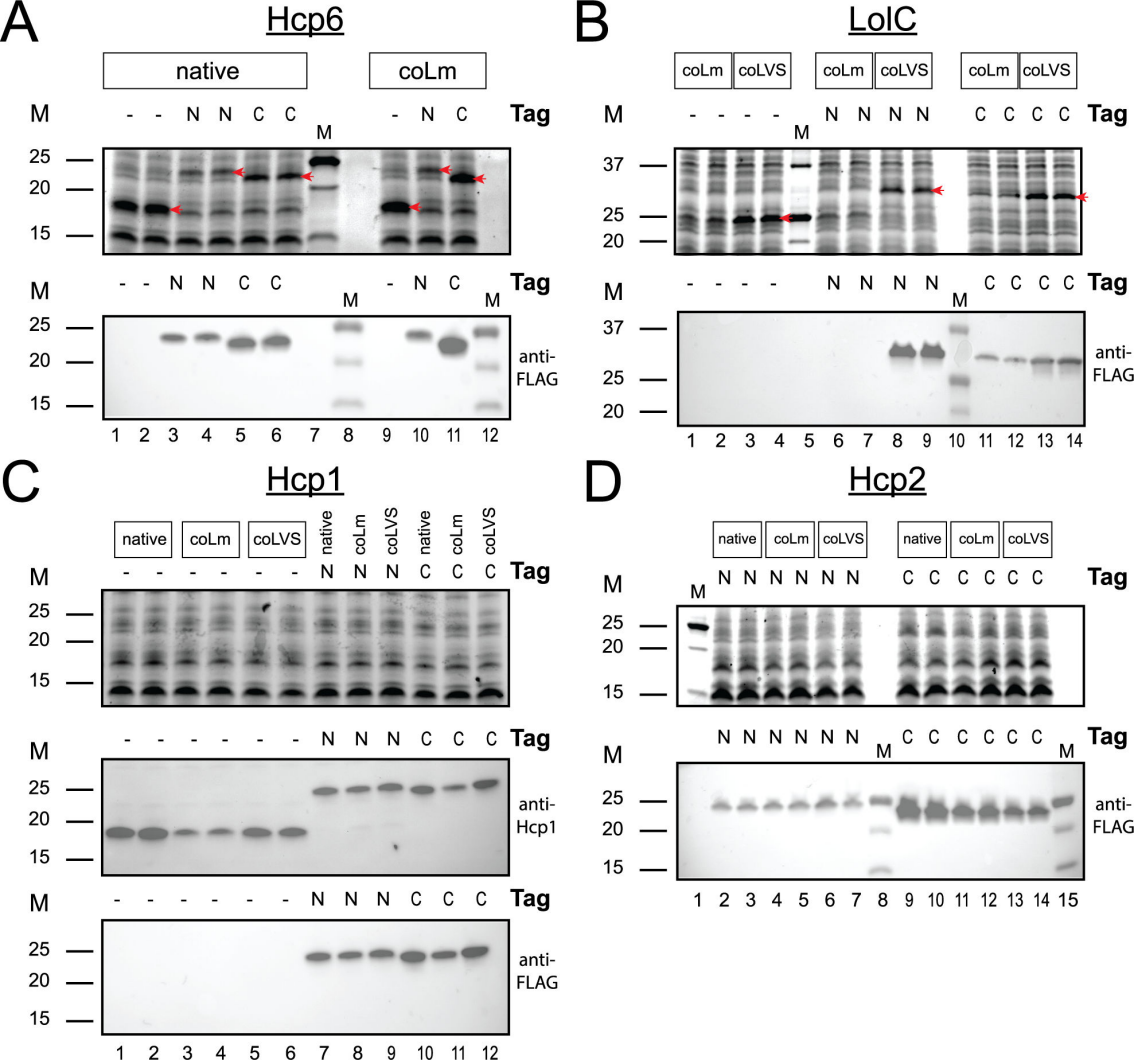
Expression of Bp antigens by rLVS Δ*capB* strains. Cleared cell lysates from rLVS Δ*capB* strains expressing Bp Hcp6 (**A**), LolC (**B**), Hcp1 (**C**), or Hcp2 (**D**) were analyzed by SDS-PAGE (upper image) and Western blotting (lower image or images) using anti-FLAG monoclonal antibody or anti-Hcp1 antibody, as indicated. The amount of lysate loaded per lane is equivalent to the amount of cells from 10 µL of overnight culture (**A and C**) or 25 µL of overnight culture (**B and D**). The version of the gene (native, codon-optimized for *L. monocytogenes* [coLm], or codon-optimized for LVS [coLVS]) and the presence or absence of a FLAG tag (N-terminal [N] or C-terminal [C]) is indicated above the lanes. Red arrows indicate the positions of the recombinant proteins in SDS-PAGE images (**A and B**). For LolC (**B**), the plasmid used to construct the two LolC (coLm) clones with an N-terminal FLAG tag was discovered to be defective after construction of the strains, thus explaining the lack of expression of LolC for these two clones. M, molecular mass markers in kDa. Lane numbers are indicated underneath the lower image for panels A–D.

We obtained strong expression of Hcp6 by LVS Δ*capB*, with or without a fusion tag, with bands visible on an SDS-PAGE gel when stained for total protein (Fig. 1A, upper image). The coLm gene (lanes 9–11) appeared to be expressed similarly to or somewhat better than the native gene (lanes 1–6); expression of the C-terminally tagged protein (lanes 5 and 6; lane 11) appeared slightly reduced compared with the untagged protein (lanes 1 and 2; lane 9, respectively), while the N-terminally tagged protein (lanes 3 and 4; lane 10) was even further reduced (for both native and coLm versions of the *hcp6* gene). Surprisingly, expression of the gene codon-optimized specifically for LVS (coLVS) was lower than expression of the gene codon-optimized specifically for *L. monocytogenes* (coLm) (data not shown), and so we decided to use the coLm version of *hcp6* for further constructs. As with Hcp6, we obtained good expression of LolC in LVS Δ*capB*, with bands visible on an SDS-PAGE gel when stained for total protein, although in this case the coLVS gene was superior to the coLm gene (Fig. 1B, upper image). Based on total protein, the untagged LolC (lanes 3 and 4) was expressed somewhat better than the C-terminally tagged LolC (lanes 13 and 14) and the N-terminally tagged protein (lanes 8 and 9). However, the N-terminally tagged coLVS version of LolC produced stronger bands than the C-terminally tagged LolC by Western blotting (Fig. 1B, lower image, lanes 8 and 9 and lanes 13 and 14). Hcp1 had seemingly less expression than Hcp6 and LolC, with no bands visible on an SDS-PAGE gel stained for total protein (Fig. 1C, upper image). The stain we used for detection of total protein becomes fluorescent after reacting with tryptophan residues. That Hcp1 only has a single tryptophan, whereas Hcp6 has four and LolC has three, may account for its apparent reduced expression compared with these other two proteins. Based on Western blotting using an anti-Hcp1 antibody (Fig. 1C, middle image), the untagged native *hcp1* gene (lanes 1 and 2) seemed to have the best expression, followed by the coLVS gene (lanes 5 and 6), and then the coLm gene (lanes 3 and 4), which had the least expression. The coLm gene also appeared to have less expression than the native and coLVS genes when tagged on the N-terminus (compare lane 8 with lanes 7 and 9) or C-terminus (compare lane 11 with lanes 10 and 12). Finally, Hcp2 (containing three tryptophan residues) also had lower expression than Hcp6 and LolC, with no bands visible on an SDS-PAGE gel stained for total protein (Fig. 1D, upper image). However, the FLAG-tagged proteins were easily detected by Western blotting (Fig. 1D, lower image). There is seemingly greater expression for C-terminal Hcp2 fusions (lanes 9–14) compared with N-terminal fusions (lanes 2–7), although this may reflect different affinities of the anti-FLAG antibody for N-terminal and C-terminal fusions and not just the expression level. Expression of N-terminally tagged Hcp2 appears similar for native, coLm, and coLVS genes, but expression of C-terminally tagged Hcp2 appears somewhat better for the native construct (lanes 9 and 10) compared with the coLm and coLVS constructs (lanes 11–14).

### Construction of Bp vaccines expressing two Bp antigens

**Fig 2:**
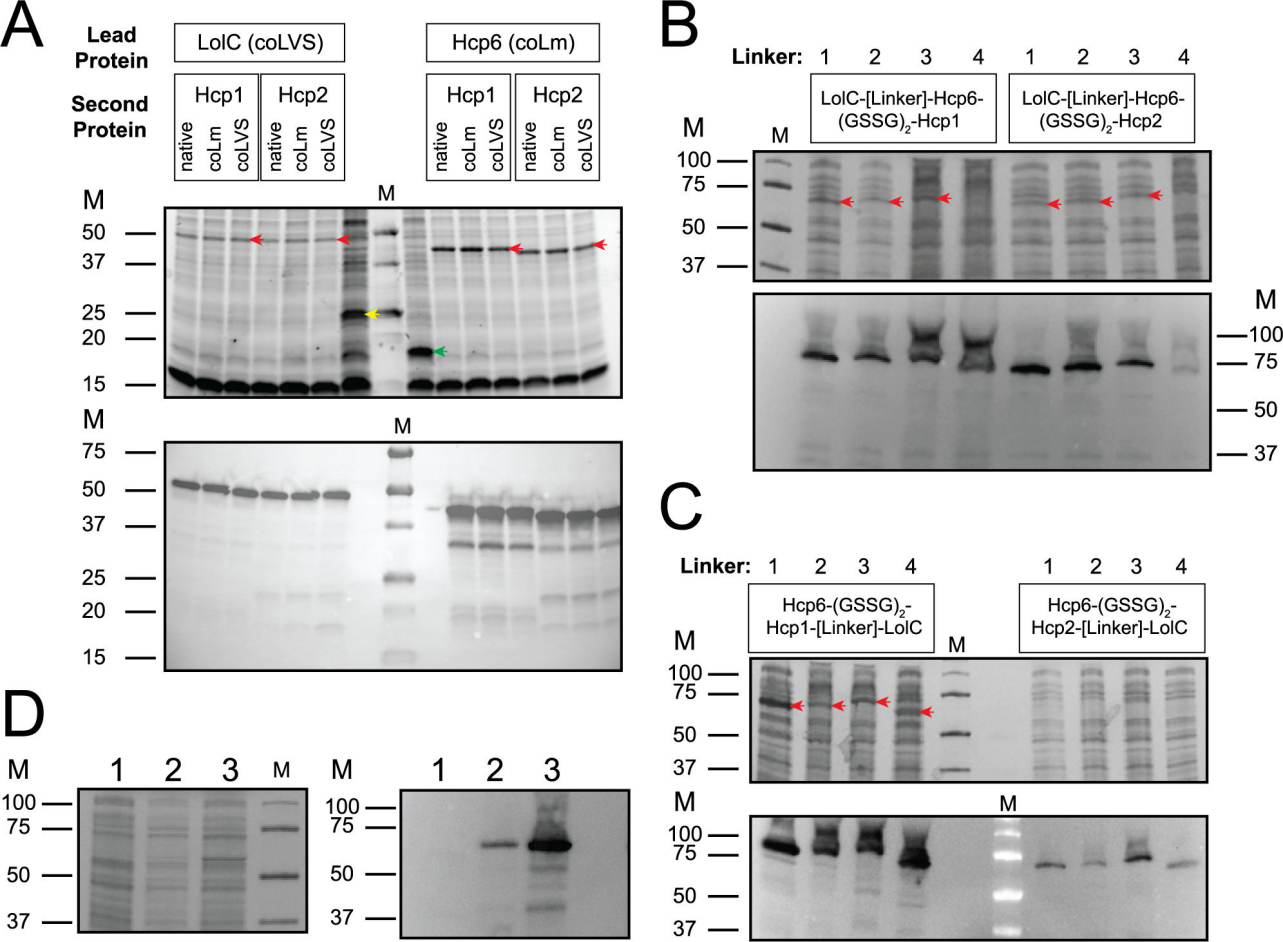
Expression of two-antigen and three-antigen fusion proteins by rLVS Δ*capB* strains. Cleared cell lysates from rLVS Δ*capB* strains expressing Bp fusion proteins with a C-terminal FLAG tag were analyzed by SDS-PAGE (upper image in panels A–C; left image in panel D) and Western blotting using anti-FLAG monoclonal antibody (lower image in panels A–C; right image in panel D). (**A)** Two-antigen fusion proteins (LolC-Hcp1, LolC-Hcp2, Hcp6-Hcp1, and Hcp6-Hcp2): The version of the gene (native, coLm, or coLVS) is indicated above the lanes. Strains expressing untagged LolC (yellow arrow) and untagged Hcp6 (green arrow) were included as controls. (B and C) Three-antigen fusion proteins (LolC-Hcp6-Hcp1, LolC-Hcp6-Hcp2, Hcp6-Hcp1-LolC, and Hcp6-Hcp2-LolC): The third antigen (LolC) was fused to either the N-terminus (**B**) or the C-terminus (**C**) of Hcp6-(GSSG)_2_-Hcp1 or Hcp6-(GSSG)_2_-Hcp2, using one of four linkers (indicated above the gels): (1) GSAGSAAGSGEF; (2) A(EAAAK)_3_A; (3) (AP)_10_; (4) direct linkage (single alanine residue). (**D)** Expression of Hcp6-Hcp1-Hcp2 by rLVS Δ*capB*. Cleared cell lysate from an rLVS Δ*capB* strain expressing the three-antigen fusion protein Hcp6-(GSSG)_2_-Hcp1-Hcp2 with a C-terminal FLAG tag (lane 3) was analyzed by SDS-PAGE (left image) and Western blotting (right image) using anti-FLAG monoclonal antibody. Lane 1: rLVS Δ*capB* strain with an empty plasmid. Lane 2: rLVS Δ*capB* strain with an alternate version of Hcp6-(GSSG)_2_-Hcp1-Hcp2. The amount of lysate loaded per lane is equivalent to the amount of cells from 20 or 25 µL of overnight culture. Red arrows indicate the positions of the recombinant three-antigen fusion proteins when apparent. M, molecular mass markers in kDa.

To increase the antigen repertoire of the rLVS Δ*capB*/Bp vaccines, we next sought to express fusion proteins consisting of two Bp antigens joined by a flexible linker. We constructed pFNL expression plasmids in which Hcp6 or LolC (the two best-expressed proteins) were the lead protein fused via a GSSGGSSG flexible peptide linker to a C-terminal Hcp1 or Hcp2 protein, electroporated the plasmids into LVS Δ*capB*, and analyzed expression by SDS-PAGE and Western blotting (Fig. 2A). Expression of the two-antigen fusion proteins was maintained at a relatively high level, similar to the individual Hcp6 and LolC proteins (Fig. 1A and B), with bands visible on an SDS-PAGE stained for total protein (Fig. 2A, upper image). Expression levels appeared to be unaffected by which gene (*hcp1* or *hcp2*) or which version of the gene (native, coLVS, or coLm) was at the C-terminus, suggesting that the lead gene (*hcp6* or *lolC*) has the greater influence on expression level.

### Efficacy of LVS Δ*capB* platform vaccines expressing combinations of two Bp antigens in the highly sensitive BALB/c mouse model of pneumonic melioidosis

**Fig 3:**
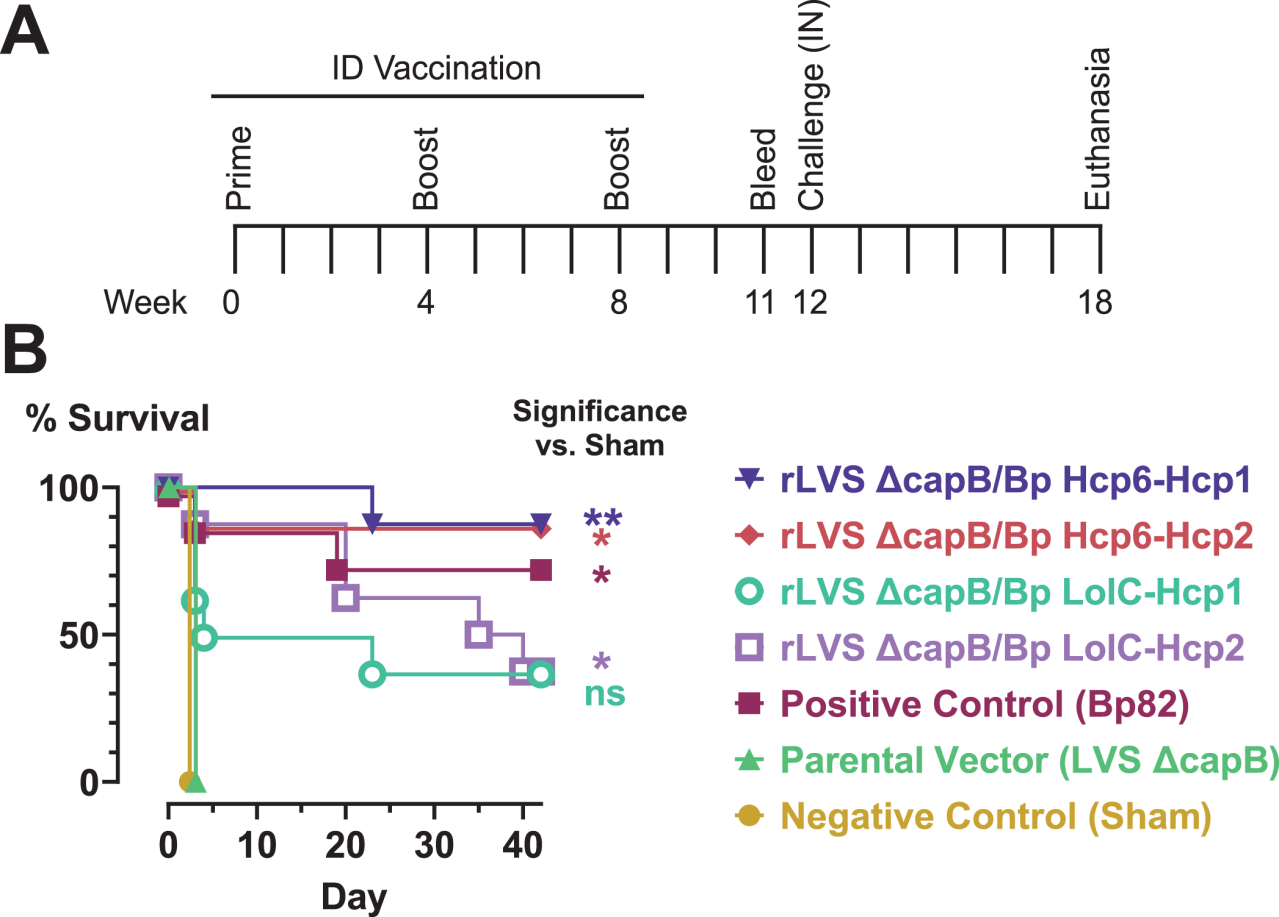
Efficacy experiment 1: rLVS Δ*capB* vaccines expressing two Bp antigens are protective against IN challenge with virulent Bp 1026b in highly sensitive BALB/c mice. (**A)** Experimental schedule. (**B)** Kaplan-Meier survival curves. BALB/c mice (*n* = 8/group) were immunized by the ID route three times, four weeks apart (weeks 0, 4, and 8) with 1 × 10^6^ CFU of LVS Δ*capB* (parental vector control), Bp82 (positive control: a moderately attenuated single deletional mutant strain of Bp), or rLVS Δ*capB* strains expressing fusion proteins of Bp antigens (Hcp6-Hcp1, Hcp6-Hcp2, LolC-Hcp1, and LolC-Hcp2). Mice sham-immunized with PBS served as a negative control. At week 12, mice were challenged by the IN route with 2,225 CFU (5× LD_50_) Bp 1026b and monitored for survival for 6 weeks. Surviving mice were euthanized and the lung, liver, and spleen were examined for abscesses and then cultured for Bp. Mice with no abscesses and no detectable Bp in the three organs were recorded as having sterile immunity (Table S11). Groups were compared using pairwise log-rank tests with Holm-Bonferroni correction of *P* values for multiple comparisons. Significance vs sham: *, *P*  <  0.05; **, *P*  <  0.01; ns, not significant (*P* ≥ 0.05).

To assess the protective efficacy of the two-antigen vaccines described above, we immunized BALB/c mice, a strain of mice especially sensitive to Bp infection, by the ID route using a homologous boosting regimen (1 × 10^6^ CFU administered at weeks 0, 4, and 8). Three control groups were included: (i) a negative control group (sham-immunized); (ii) a positive control group immunized with Bp82, a moderately attenuated strain of Bp that induces good protection (38), but is not suitable for human use as it only has a single gene deletion; and (iii) the parental vector (LVS Δ*capB*). At week 12, the mice were challenged by the IN route with a lethal dose of Bp 1026b (2,225 CFU, 5× LD_50_; LD_50_ = 450 CFU) and monitored for survival for 6 weeks (Fig. 3). Sham-immunized mice and mice immunized with LVS Δ*capB* succumbed rapidly to infection with Bp. Survival of all other groups was statistically significantly better than for sham-immunized mice, except for the rLVS Δ*capB*/Bp LolC-Hcp1 group. Mice immunized with two recombinant vaccines, those expressing Hcp6-Hcp1 and Hcp6-Hcp2, had greater survival than mice immunized with Bp82 (88% vs 75%), while mice immunized with two other vaccines, expressing LolC-Hcp1 and LolC-Hcp2, had lower survival (38%).

At the end of the 6-week period for monitoring survival, the surviving mice were euthanized and the lung, liver, and spleen were examined for abscesses and then cultured for Bp. Mice with no abscesses and no detectable Bp in the three organs were recorded as having sterile immunity. We obtained moderate to high levels of sterile immunity with rLVS Δ*capB*/Bp vaccines, comparable, and in some cases superior, to that achieved with Bp82 (Table S11).

### Construction of vaccines expressing three or four Bp antigens

As we obtained good protection with rLVS Δ*capB*/Bp vaccines expressing two antigen fusion proteins in experiment 1 (Hcp6-Hcp1, Hcp6-Hcp2, LolC-Hcp1, and LolC-Hcp2, Fig. 3), but not 100% protection, we sought to determine if we could improve upon our results by adding additional antigens. Therefore, we constructed vaccines expressing three-antigen fusion proteins by linking LolC to either the N-terminus or C-terminus of Hcp6-Hcp1 or Hcp6-Hcp2 (two of the best constructs from experiment 1). For each combination of antigens, the C-terminal portion was linked to the N-terminal portion by one of three peptide linkers or linked directly (only a single alanine inserted between the two). We transformed the 16 plasmids into LVS Δ*capB*, and analyzed individual clones for expression of the three-antigen fusion proteins by SDS-PAGE and Western blotting (Fig. 2B and C). Seven of the eight constructs with LolC as the lead protein were expressed well (Fig. 2B), but LolC linked directly to Hcp6-Hcp2 had poor expression (Fig. 2B, right section, linker 4). All four constructs comprising Hcp6-Hcp1-LolC were expressed well and the type of linkage between Hcp6-Hcp1 and LolC seemed to have minimal influence on expression level (Fig. 2C, left section, linkers 1–4). In the case of Hcp6-Hcp2-LolC, all four constructs were expressed poorly. We selected one of the best-expressing constructs, Hcp6-Hcp1-GSAGSAAGSGEF-LolC, to pursue further (Fig. 2C, left section, linker 1). We also constructed an rLVS Δ*capB*/Bp vaccine expressing Hcp6-Hcp1-Hcp2 with Hcp2 linked directly to Hcp6-Hcp1, which had moderate expression (Fig. 2D, lane 3).

To construct vaccines with all four antigens, we joined two of the two-antigen constructs that were expressed well (Hcp6-Hcp1 and LolC-Hcp2, Fig. 2A) with various linkers. As before, the Hcp6-Hcp1 fusion protein was expressed very well, as were derivatives of Hcp6-Hcp1 with peptide linkers on the C-terminus (but lacking LolC-Hcp2) (Fig. S1). Also, as previously observed, LolC-Hcp2 was expressed well enough to be visualized with a total protein stain. However, the four-antigen fusion proteins (Hcp6-Hcp1-LolC-Hcp2), although present, were barely detectable by Western blotting (Fig. S1C) and so this strategy was abandoned. We then tried a second approach to construct vaccines with all four antigens, by linking Hcp6-Hcp1-Hcp2 with LolC fused to either the N-terminus or the C-terminus. As for the three-antigen constructs, the C-terminal portion was linked to the N-terminal portion by one of three peptide linkers or directly. We failed to obtain a correct plasmid for one of the eight constructs and did not pursue it further; the correct seven plasmids were transformed into LVS Δ*capB*, and individual clones were analyzed for expression of the four-antigen fusion proteins by SDS-PAGE and Western blotting (Fig. S2). Although none of the four-antigen fusion proteins were apparent in a total protein stain, six of the seven fusion proteins were detectable by Western blotting (A, linkers 2 and 3; B linkers 1–4). We selected one of the constructs, LolC-(AP)_10_-Hcp6-Hcp1-Hcp2 (A, linker 3), to test as a vaccine.

### Efficacy of LVS Δ*capB* platform vaccines expressing combinations of three Bp antigens in the BALB/c mouse model of pneumonic melioidosis and dose-response study of two-antigen vaccines

**Fig 4:**
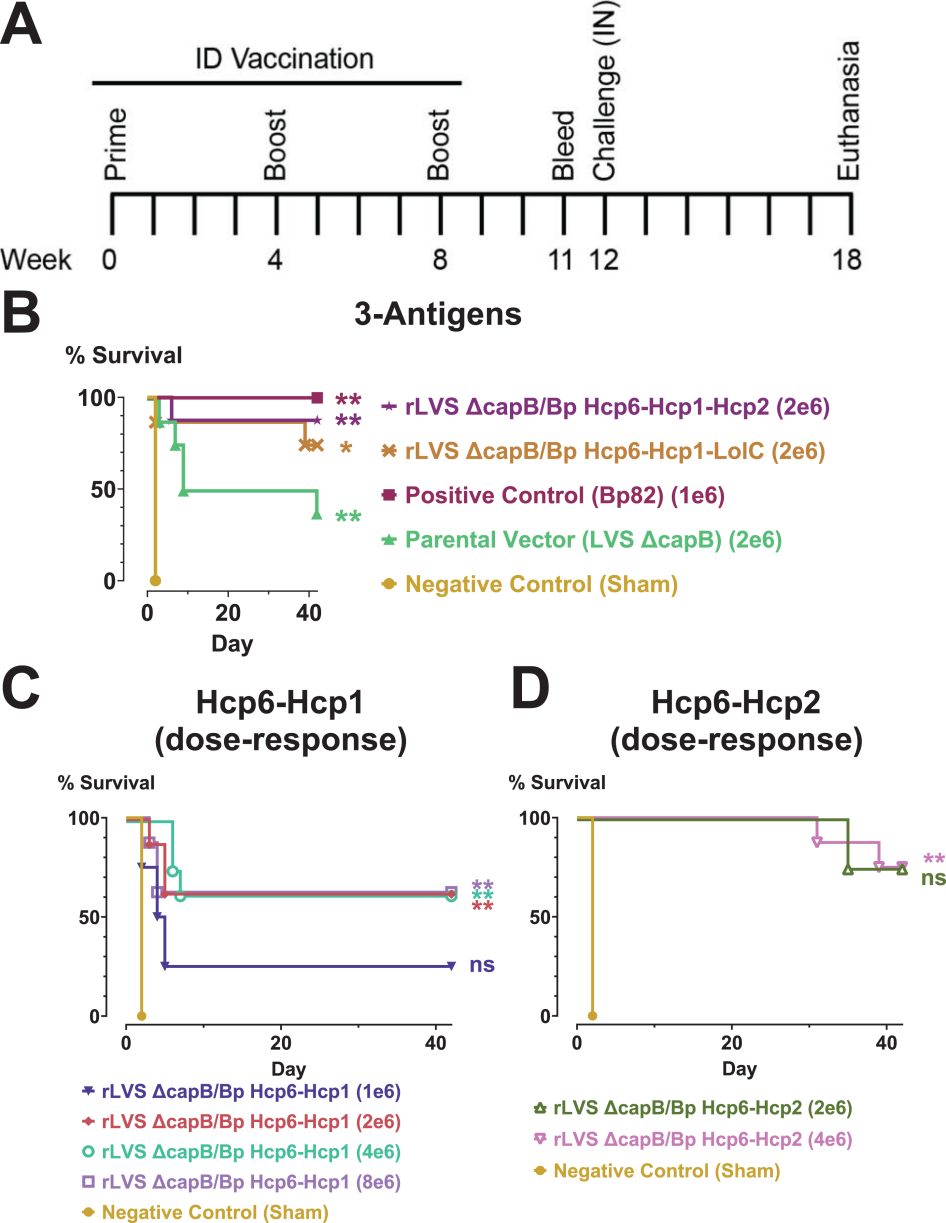
Efficacy experiment 2: rLVS Δ*capB* vaccines expressing three Bp antigens are comparable to or better than vaccines expressing two Bp antigens and immunization doses of ≥2 × 10^6^ CFU provide optimal protection. (**A)**. Experimental schedule. (B–D) Kaplan-Meier survival curves. (**B)** Efficacy of three-antigen vaccines. BALB/c mice (*n* = 8/group) were sham-immunized with PBS (negative control) or immunized by the ID route three times, four weeks apart (weeks 0, 4, and 8) with 2 × 10^6^ CFU of LVS Δ*capB* (parental vector control), 1 × 10^6^ CFU Bp82 (positive control), or 2 × 10^6^ CFU rLVS Δ*capB* vaccines expressing fusion proteins of Bp antigens (Hcp6-Hcp1-Hcp2 or Hcp6-Hcp1-LolC). (C and D) Dose-response experiment. In the same experiment, BALB/c mice were immunized with 1, 2, 4, or 8 × 10^6^ CFU Hcp6-Hcp1 (**C**) or 2 or 4 × 10^6^ CFU Hcp6-Hcp2 (**D**); all groups had eight mice except for the group immunized with the Hcp6-Hcp2 vaccine at 2 × 10^6^ CFU, which had only four mice. (B–D) At week 12, mice were challenged by the IN route with 1,800 CFU (4× LD_50_) Bp 1026b and monitored for survival for 6 weeks. The negative control is shown on all three graphs, but the positive control and parental vector are omitted from the graphs in C and D to facilitate visualization of the dose-response groups. Surviving mice were euthanized and the lung, liver, and spleen were examined for abscesses and then cultured for Bp. Mice with no abscesses and no detectable Bp in the three organs were recorded as having sterile immunity (Table S11). Groups were compared using pairwise log-rank tests with Holm-Bonferroni correction of *P* values for multiple comparisons. Significance vs sham: *, *P*  <  0.05; **, *P*  <  0.01; ns, not significant (*P* ≥ 0.05). Although the group immunized with the Hcp6-Hcp2 vaccine at 2 × 10^6^ CFU had good protection, it was not significant vs sham probably due to insufficient statistical power with only four mice in this vaccine group.

To evaluate the efficacy of three-antigen vaccines, we immunized BALB/c mice by the ID route using a homologous boosting regimen (weeks 0, 4, and 8) as in experiment 1 (Fig. 4). We administered the new three-antigen vaccines and the LVS Δ*capB* parental vector at 2 × 10^6^ CFU (Fig. 4B). Simultaneously, to determine the optimal dose of vaccine, we selected the two best vaccines from experiment 1 (expressing Hcp6-Hcp1 and Hcp6-Hcp2) and performed a dose-response study, using 1, 2, 4, or 8 × 10^6^ CFU for Hcp6-Hcp1 (Fig. 4C) and 2 or 4 × 10^6^ CFU for Hcp6-Hcp2 (Fig. 4D). A negative control group (sham-immunized) and a positive control group (immunized with Bp82) were included as in experiment 1. At week 12, mice were challenged by the IN route with a lethal dose of Bp 1026b (1,800 CFU, 4× LD_50_) and monitored for survival for 6 weeks.

As in experiment 1, sham-immunized mice succumbed rapidly to infection with Bp. However, in contrast to experiment 1, mice immunized with LVS Δ*capB*, the parental vector, were moderately protected. The difference in results may be due to using a higher vaccination dose (2 × 10^6^ vs 1 × 10^6^ CFU) and/or a somewhat lower challenge dose in experiment 2 (1,800 vs 2,225 CFU).

All vaccines tested in experiment 2 produced statistically significant protection compared with sham-vaccinated mice, with the exception of the lowest dose of the Hcp6-Hcp1 vaccine and Hcp6-Hcp2 vaccine, the latter of which only had 4 mice in the group. The new three-antigen vaccines expressing Hcp6-Hcp1-Hcp2 or Hcp6-Hcp1-LolC provided good protection (88% and 75% survival, respectively), comparable or superior to the two-antigen vaccines expressing Hcp6-Hcp1 or Hcp6-Hcp2, although the differences were not statistically significant. We obtained moderate levels of sterile immunity for the three-antigen vaccines, comparable to that achieved with Bp82 (Table S11). Based on these results, we decided to move forward with the three-antigen vaccines reasoning that a greater antigen repertoire may be beneficial in outbred populations.

In the same experiment, a vaccine expressing a four-antigen fusion protein, LolC-(AP)_10_-Hcp6-Hcp1-Hcp2, was also tested at a dose of 2 × 10^6^ CFU. Immunization with this vaccine resulted in 50% survival, somewhat less than the efficacy of the three-antigen vaccines (data not shown).

In the dose-response study (Fig. 4C and D ) , the lowest dose of the Hcp6-Hcp1 vaccine (1 × 10^6^ CFU) had the lowest survival of any of the rLVS Δ*capB*/Bp vaccine groups. Since there was no apparent advantage to doses greater than 2 × 10^6^ CFU, and potentially lesser efficacy at 1 × 10^6^ CFU, we decided to use 2 × 10^6^ CFU as the dose in subsequent experiments.

### Three-antigen vaccines delivered intranasally induce potent protection against high-dose Bp challenge in the BALB/c mouse model of pneumonic melioidosis and are efficacious with a single immunization

**Fig 5:**
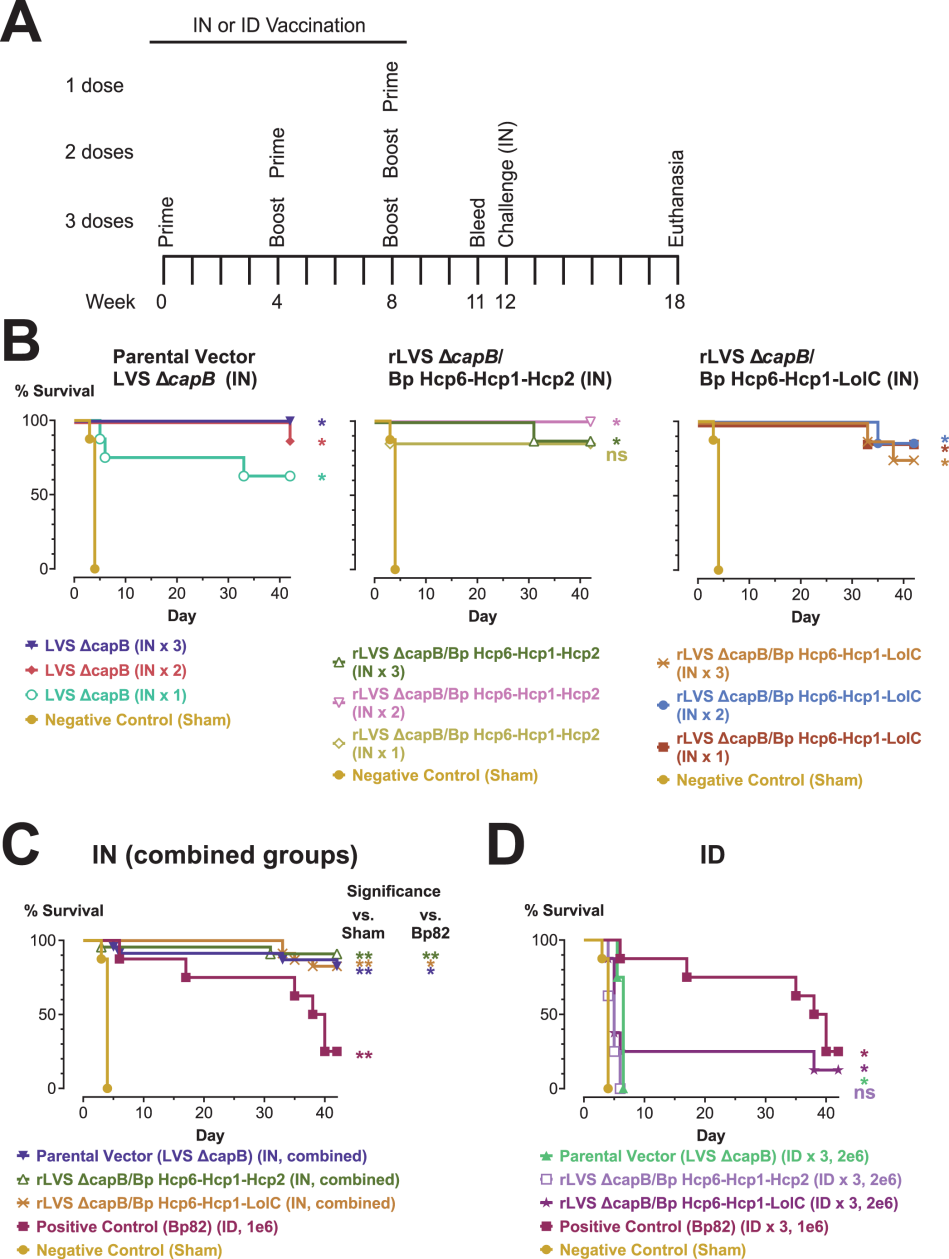
Efficacy experiment 3: rLVS Δ*capB* vaccines expressing three Bp antigens administered by the IN route are highly protective against a high challenge dose of Bp 1026b. (**A)** Experimental schedule. (B–D) Kaplan-Meier survival curves. BALB/c mice (*n* = 7 or 8/group) were immunized by the IN (**B, C**) or ID (**D**) route three times, four weeks apart (weeks 0, 4, and 8) with 2 × 10^6^ CFU of LVS Δ*capB* (parental vector control) or rLVS Δ*capB* strains expressing fusion proteins of Bp antigens (Hcp6-Hcp1-Hcp2 and Hcp6-Hcp1-LolC). Additional groups of mice were immunized by the IN route with only one dose (week 8) or two doses (weeks 4 and 8) of vaccine while maintaining a constant immunization-challenge interval. The positive control strain, Bp82, was administered at 1 × 10^6^ CFU by the ID route, as in the first two animal efficacy experiments (Fig. 3 and Fig. 4) and is shown in both panels C and D. Mice sham-immunized with PBS served as a negative control and are shown in all graphs. (B–D) At week 12 (4 weeks after the last boost), mice were challenged by the IN route with 2,700 CFU (6× LD_50_) Bp 1026b and monitored for survival for 6 weeks. (**B**) IN immunization with one, two, or three doses of vaccine. (**C**) IN immunization, combining groups for the same vaccine from (**B)** that received one, two, or three doses. (**D**) ID immunization with three doses of vaccine. Surviving mice were euthanized and the lung, liver, and spleen were examined for abscesses and then cultured for Bp. Mice with no abscesses and no detectable Bp in the three organs were recorded as having sterile immunity (Table S11). Groups were compared using pairwise log-rank tests with Holm-Bonferroni correction of *P* values for multiple comparisons. Significance vs sham (and vs Bp82 in panel C): *, *P*  <  0.05; **, *P*  <  0.01; ns, not significant (*P* ≥ 0.05).

In our third efficacy experiment, we tested IN delivery of our two three-antigen vaccines (expressing Hcp6-Hcp1-Hcp2 and Hcp6-Hcp1-LolC) to determine if this might further improve protective efficacy (Fig. 5). We immunized BALB/c mice with 2 × 10^6^ CFU of these vaccines and the LVS Δ*capB* parental vector by the IN route using a homologous boosting regimen (weeks 0, 4, and 8). Additionally, to determine the optimal number of doses of vaccine, we immunized other groups of mice with only one dose (week 8) or two doses (weeks 4 and 8) of vaccine instead of the usual three doses while maintaining a constant immunization-challenge interval. A negative control group (sham-immunized) and a positive control group (immunized with 1 × 10^6^ CFU Bp82 by the ID route) were included as in experiments 1 and 2. At week 12, mice were challenged by the IN route with a lethal dose of Bp 1026b (2,700 CFU, 6× LD_50_) and monitored for survival for 6 weeks (Fig. 5).

As in previous experiments, sham-immunized mice succumbed rapidly to infection with Bp (Fig. 5B). Bp82, while still protective, had the worst survival of the three experiments (25% vs 75% and 100%) (Fig. 5C and D), presumably due to experiment 3 having the highest challenge dose of the three experiments (2,700 CFU vs 2,225 and 1,800 CFU). Despite the high challenge dose, the six groups immunized by the IN route with the two three-antigen vaccines (one, two, or three doses) had very high survival (75–100%) (Fig. 5B, center and rightmost graphs). The LVS Δ*capB* parental vector was also highly protective by the IN route (Fig. 5B, leftmost graph). Although 1 dose of LVS Δ*capB* was not as effective as two or three doses (63% vs 88% and 100%, respectively), the differences in the survival curves were not statistically significant. Despite the high efficacy of the three-antigen rLVS Δ*capB*/Bp vaccines and the LVS Δ*capB* parental vector, most groups had lower sterile immunity than observed in the two previous experiments, as did Bp82 (Table S11). This likely reflects the higher challenge dose in experiment 3. Although all nine IN vaccine groups had better protection than the Bp82 positive control (62.5–100% survival vs 25% survival), individually differences between the survival curves were not statistically significant. However, combining groups for the same vaccine that received one, two, or three doses to increase statistical power demonstrates the superiority of IN vaccination with the LVS Δ*capB* vaccine platform over ID immunization with Bp82 (Fig. 5C).

### Three-antigen vaccines delivered intranasally provide long-term protection in the BALB/c mouse model of pneumonic melioidosis

**Fig 6:**
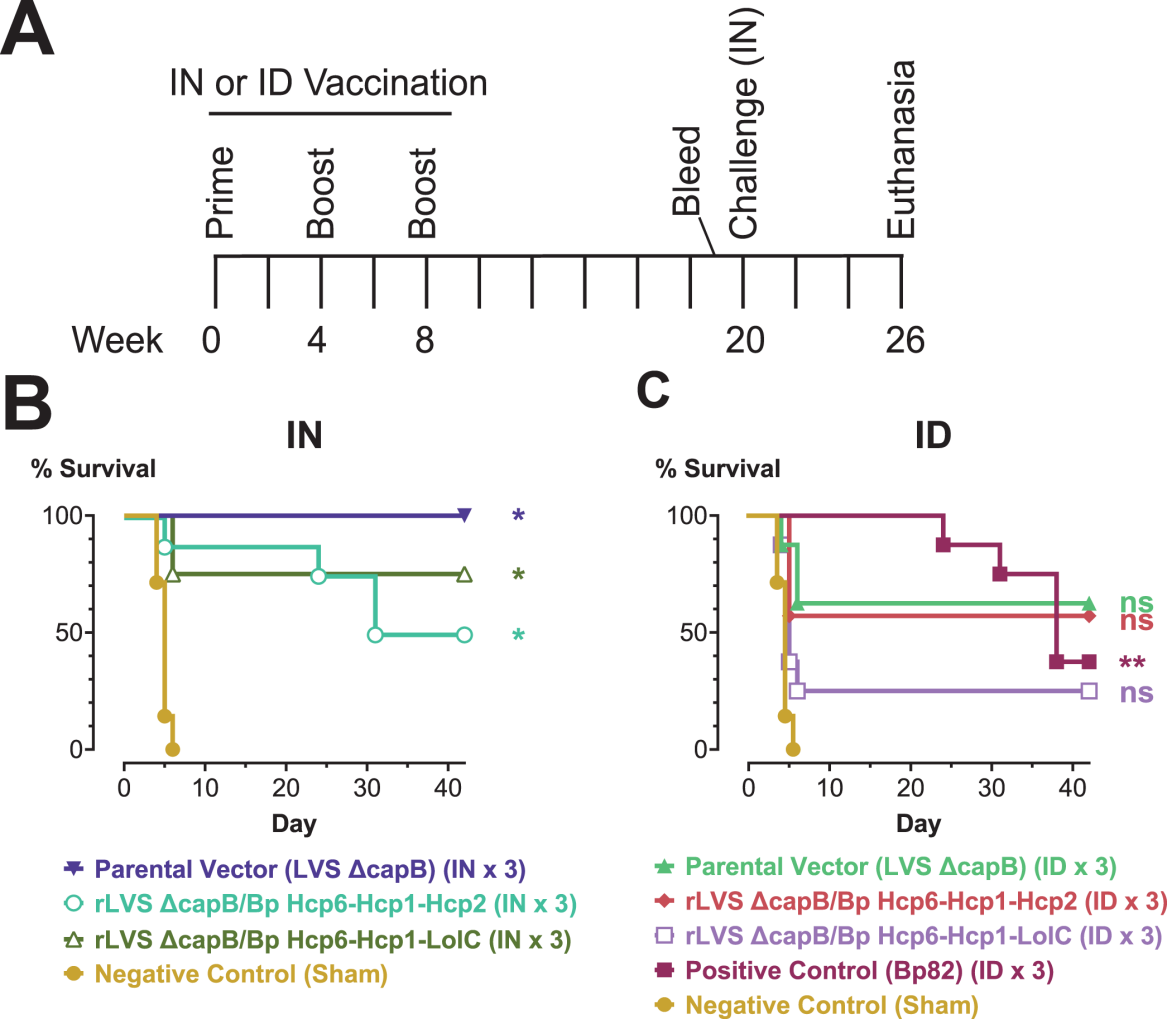
Efficacy experiment 4: rLVS Δ*capB* vaccines expressing three Bp antigens administered by the IN route provide long-term protection against a high challenge dose of bp 1026b. (**A)** Experimental schedule. (**B and C)** Kaplan-Meier survival curves. BALB/c mice (*n* = 7 or 8/group) were immunized by the IN (**B**) or ID (**C**) route three times, four weeks apart (weeks 0, 4, and 8) with 2 × 10^6^ CFU of LVS Δ*capB* (parental vector control) or rLVS Δ*capB* strains expressing fusion proteins of Bp antigens (Hcp6-Hcp1-Hcp2 and Hcp6-Hcp1-LolC) as in Fig. 5. Control mice were sham-immunized or immunized ID with the positive control strain Bp82 as in the previous efficacy experiments. At week 20 (12 weeks after the last boost), mice were challenged by the IN route with 1,430 CFU (3.2× LD_50_) Bp 1026b and monitored for survival for 6 weeks. Surviving mice were euthanized and the lung, liver, and spleen were examined for abscesses and then cultured for Bp. Mice with no abscesses and no detectable Bp in the three organs were recorded as having sterile immunity (Table S11). Groups were compared using pairwise log-rank tests with Holm-Bonferroni correction of *P* values for multiple comparisons. Significance vs sham: *, *P*  <  0.05; **, *P*  <  0.01; ns, not significant (*P* ≥ 0.05).

In our fourth efficacy experiment (Fig. 6), we investigated the capacity of the two three-antigen vaccines (expressing Hcp6-Hcp1-Hcp2 and Hcp6-Hcp1-LolC) to protect against delayed challenge with a lethal dose of Bp 1026b. We immunized BALB/c mice with 2 × 10^6^ CFU of these vaccines or the LVS Δ*capB* parental vector by the IN or ID route using a homologous boosting regimen (weeks 0, 4, and 8). Controls were sham-immunized or immunized with Bp82 ID as in previous experiments. At week 20 (12 weeks after the last boost), mice were challenged by the IN route with 1,430 CFU (3.2× LD_50_) Bp 1026b and monitored for survival for 6 weeks. When delivered by the IN route (Fig. 6B), both vaccines were highly protective and provided a relatively high level of sterile immunity (50% and 63%); interestingly, the LVS Δ*capB* vector by itself was also highly protective by the IN route and similarly provided a high level of sterile immunity (67%) (Table S11). In the same experiment, Bp82 provided a somewhat lower level of protection from survival and 0% sterile immunity (Fig. 6C; Table S11). The vaccines and vector delivered ID were less protective (difference in survival between vaccine or vector and sham-immunized animals not statistically significant).

### Intranasal vaccination is superior to intradermal vaccination in the BALB/c mouse model of pneumonic melioidosis and protects against even high-dose Bp lethal respiratory challenge

**Fig 7:**
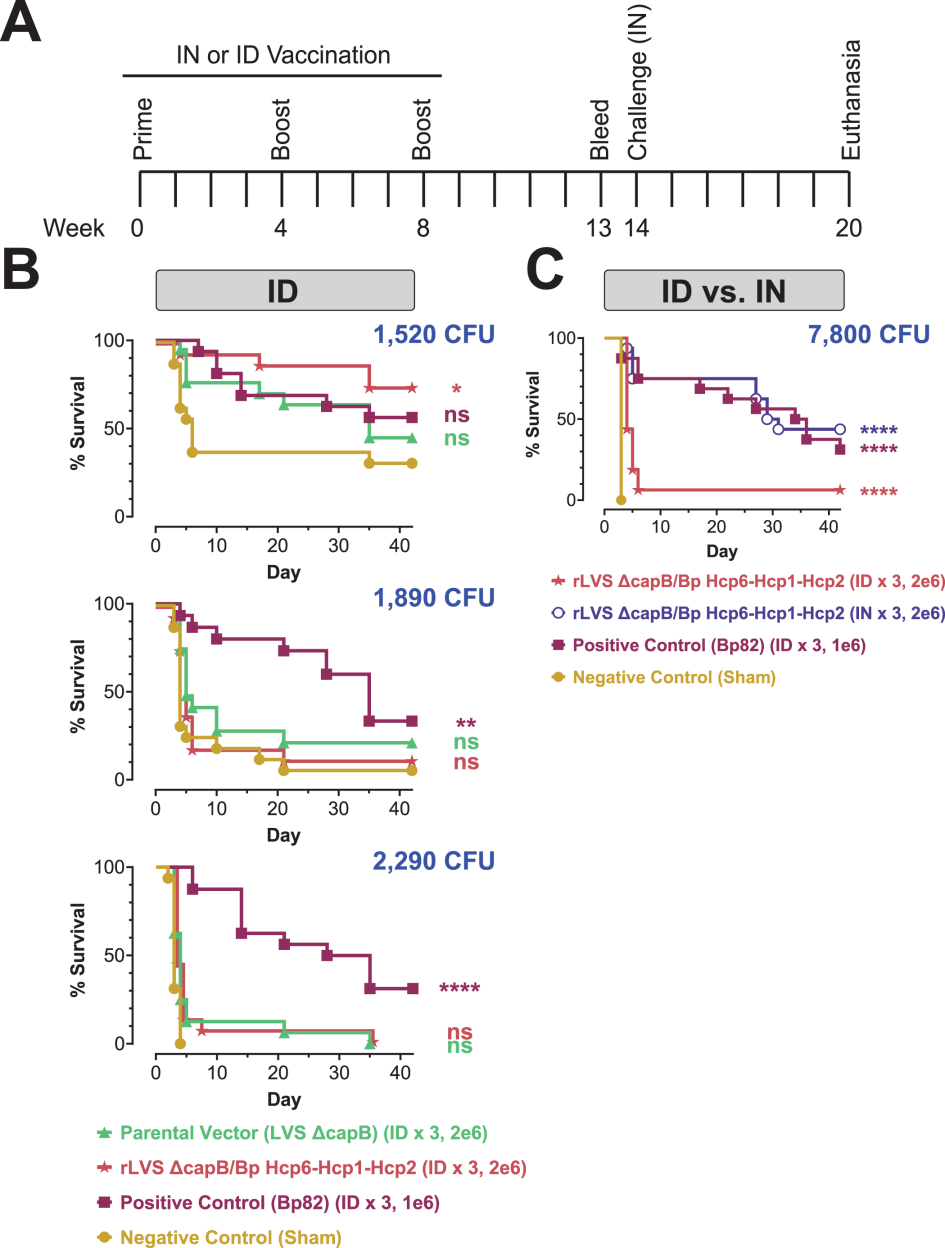
Efficacy experiments 5 and 6: rLVS Δ*capB* vaccine expressing Hcp6-Hcp1-Hcp2 administered by the IN route is protective against a very high challenge dose of Bp 1026b. (**A)**. Experimental schedule. (**B and C)** Kaplan-Meier survival curves. BALB/c mice (*n* = 15 or 16/group) were immunized by the ID route (efficacy experiment 5) (**B**) or the ID or IN route (efficacy experiment 6) (**C**) three times, four weeks apart (weeks 0, 4, and 8) with 2 × 10^6^ CFU of LVS Δ*capB* (parental vector control) or rLVS Δ*capB* expressing the Hcp6-Hcp1-Hcp2 fusion protein. The positive control strain, Bp82, was administered at 1 × 10^6^ CFU by the ID route, as usual. Mice sham-immunized with PBS served as a negative control. At week 14 (6 weeks after the last boost), in experiment 5 (**B**), mice were challenged by the IN route with 1,520 CFU (1.3× LD_50_), 1,890 CFU (1.6× LD_50_), or 2,290 CFU (1.9× LD_50_) Bp 1026b. In experiment 6 (**C**), mice were challenged with 7,800 CFU (2.8× LD_50_, based on a newly calculated LD_50_) Bp 1026b. Mice surviving for 6 weeks were euthanized and the lung, liver, and spleen were examined for abscesses and then cultured for Bp. Mice with no abscesses and no detectable Bp in the three organs were recorded as having sterile immunity (Table S11). Groups were compared using pairwise log-rank tests with Holm-Bonferroni correction of *P* values for multiple comparisons. Significance vs sham: *, *P* <0.05, **, *P*  <  0.01; ****, *P*  <  0.0001; ns, not significant (*P* ≥ 0.05).

Since we had mixed results with ID vaccination, sometimes producing good protection and sometimes not, and because after the first efficacy experiment, we did not have a clear, statistically significant difference in efficacy between the LVS Δ*capB* vector control and the rLVS Δ*capB*/Bp vaccines expressing Bp antigens, we performed a fifth efficacy experiment comparing rLVS Δ*capB*/Bp Hcp6-Hcp1-Hcp2 and the LVS Δ*capB* vector delivered by the ID route, at three different challenge doses (Fig. 7B). We immunized BALB/c mice with 2 × 10^6^ CFU of rLVS Δ*capB*/Bp Hcp6-Hcp1-Hcp2 or the LVS Δ*capB* parental vector using a homologous boosting regimen (weeks 0, 4, and 8). Controls were sham-immunized or immunized with Bp82 ID as in previous experiments. At week 14 (6 weeks after the last boost), mice were challenged by the IN route with 1,520 CFU, 1,890 CFU, or 2,290 CFU Bp 1026b and monitored for survival for 6 weeks (Fig. 7B). In contrast to all of our previous experiments, we did not obtain 100% killing of the sham-vaccinated mice at the two lower challenge doses (1,520 and 1,890 CFU; 3.4× and 4.2× LD_50_ based on our original LD_50_ = 450 CFU). Analysis of the sham-vaccinated mice from this study as well as mice from an LD_50_ study we ran concurrently (data not shown), resulted in a revised LD_50_ of 1,200 CFU. At the lowest challenge dose (1,520 CFU), mice vaccinated with rLVS Δ*capB*/Bp Hcp6-Hcp1-Hcp2 had better survival than sham-vaccinated mice, whereas mice vaccinated with the LVS Δ*capB* vector did not show a statistically significant difference (Fig. 7B, top graph). Although rLVS Δ*capB*/Bp Hcp6-Hcp1-Hcp2 also had better survival than the LVS Δ*capB* vector, this difference was not statistically significant. At the two higher challenge doses, both vaccines administered by the ID route performed poorly (Fig. 7B, middle and bottom graphs).

In our sixth, and final, efficacy experiment, we compared ID vs IN vaccination for the rLVS Δ*capB*/Bp Hcp6-Hcp1-Hcp2 vaccine using a very high challenge dose of 7,800 CFU (2.8× LD_50_ based on an LD_50_ study completed shortly before the challenge, LD_50_ = 2,800 CFU, data not shown). (Fig. 7C). Controls were sham-immunized or immunized with Bp82 by the ID route, as in previous experiments. We challenged 6 weeks after the last boost and monitored the mice for 6 weeks. As in the previous experiment at high challenge doses, the protective efficacy of rLVS Δ*capB*/Bp Hcp6-Hcp1-Hcp2 was poor when administered by the ID route (although statistically better than sham-vaccinated mice), whereas IN vaccination worked much better. Administered IN, the vaccine was equivalent in potency to the Bp82 positive control.

**Fig 8:**
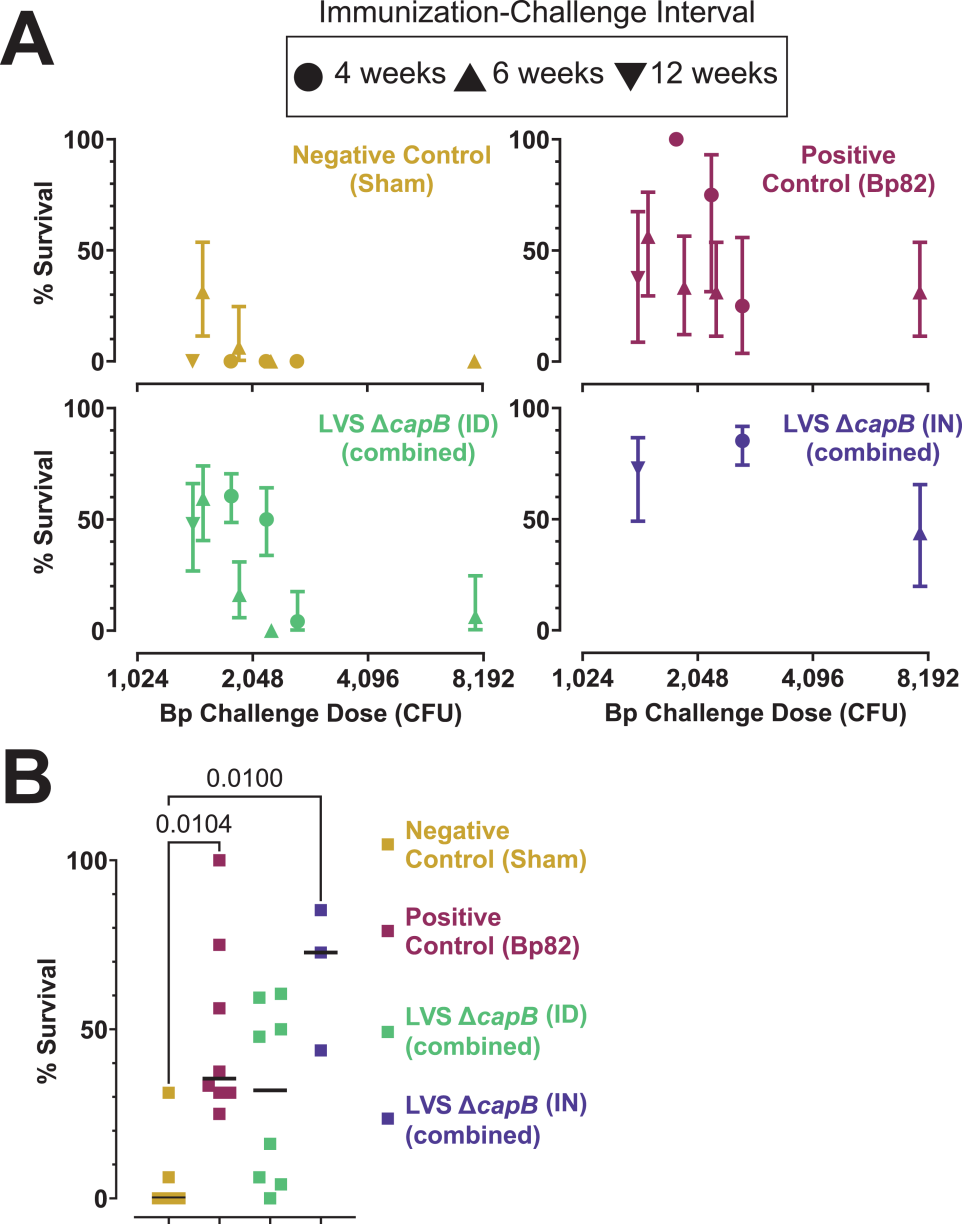
Superiority of intranasal vaccination compared with intradermal vaccination. (**A)** Survival vs challenge dose. The final survival percentage with 95% confidence intervals is shown for each experiment, with each group displayed on a separate graph. The group “LVS Δ*capB* (ID) combined,” represents the data from all LVS Δ*capB* and rLVS Δ*capB* vaccines administered by the ID route in a particular experiment. Likewise, the group “LVS Δ*capB* (IN) combined,” represents the data from all LVS Δ*capB* and rLVS Δ*capB* vaccines administered by the IN route in a particular experiment. For efficacy experiment 6, there was only one ID and one IN group. Efficacy experiment 5 had three separate challenge doses which are plotted individually. The symbol shape indicates the length of the immunization-challenge interval for that particular experiment: circle, 4 weeks; triangle, 6 weeks; inverted triangle, 12 weeks. (**B)** Survival for all experiments. Each symbol represents the percent survival for an individual experiment at a specific challenge dose from panel A. Horizontal bar indicates median survival. Groups were compared using the Kruskal-Wallis non-parametric test with Dunn’s multiple comparisons test (Prism 9.3.1) and *P* values <0.05 are shown on the graph.

We further analyzed the results of all six efficacy experiments to compare ID with IN vaccination and the role of challenge dose (Fig. 8). For this analysis, we treated all groups within an experiment that were vaccinated by the ID route with LVS Δ*capB* and rLVS Δ*capB*/Bp vaccines as a single group [LVS Δ*capB* (ID) combined]. Similarly, all groups within an experiment that were vaccinated by the IN route were treated as a single group [LVS Δ*capB* (IN) combined]. Sham-vaccinated mice showed 0% survival except in two experiments with low challenge doses (Fig. 8A, upper left graph). Bp82-vaccinated mice showed moderate to high survival at all challenge doses (Fig. 8A, upper right graph). LVS Δ*capB* vaccines administered IN were consistently protective in the three experiments where the IN route was tested, even at high challenge doses, and was consistently superior to Bp82 vaccination within the same experiment (Fig. 8A, lower right graph). LVS Δ*capB* vaccines administered ID, however, showed decidedly mixed results, with good protection in half the studies and almost no protection in the other half (Fig. 8A , lower left graph). The latter mostly involved higher challenge doses, suggesting that protective efficacy induced by ID vaccination is more readily overwhelmed than that induced by IN vaccination. Combining all experiments without regard to the magnitude of the challenge dose, LVS Δ*capB* vaccines administered IN but not ID induced statistically significant protection compared with sham-vaccinated mice (*P* = 0.01) (Fig. 8B).

### Serum IgG response to LVS Δ*capB* platform vaccines expressing two or more Bp antigens

**Fig 9:**
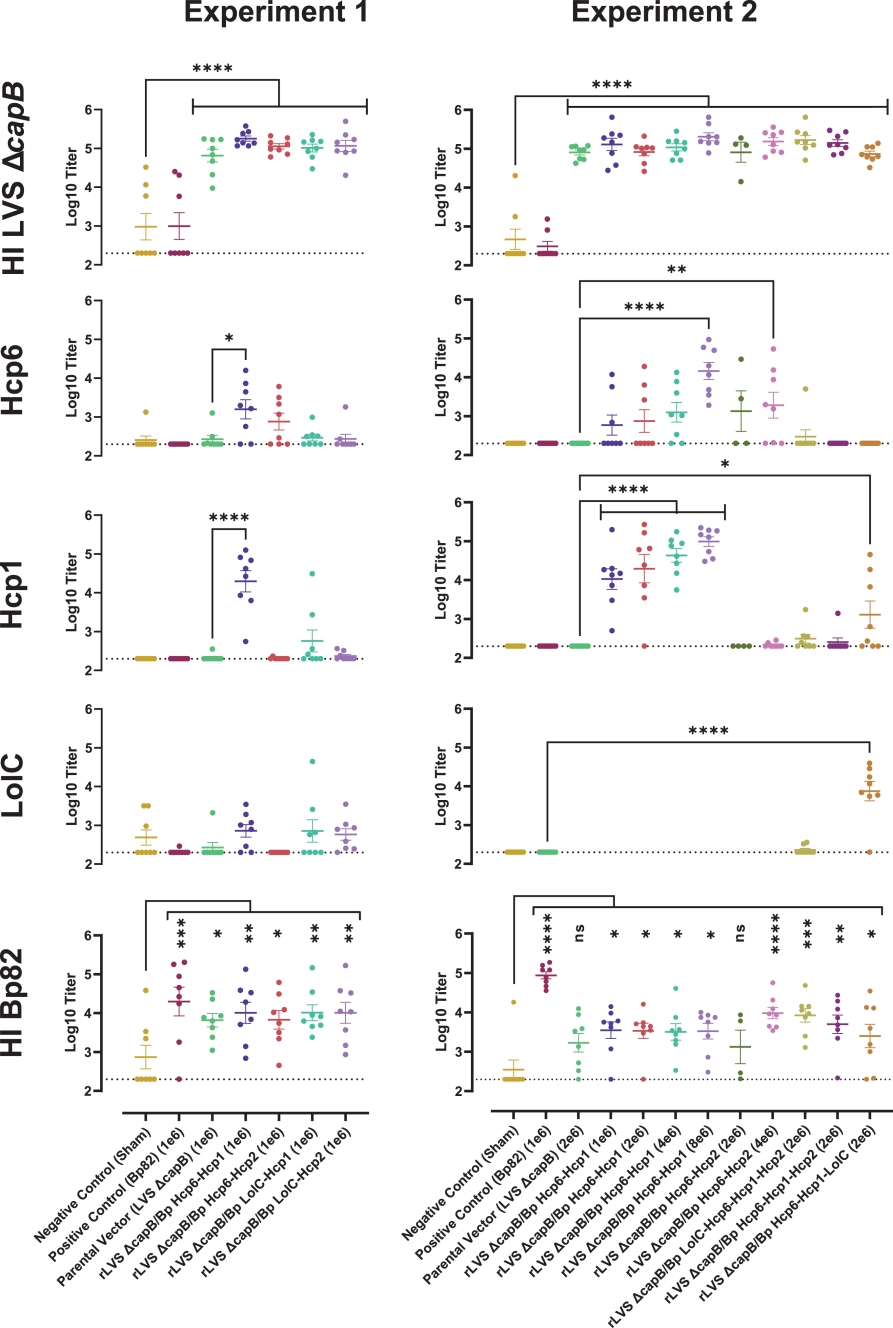
Serum IgG titers to LVS and Bp antigens. Immunized mice from experiment 1 (Fig. 3) and experiment 2 (Fig. 4) were bled 1 week prior to challenge and serum IgG titers for five antigens (HI LVS Δ*capB*, Hcp6, Hcp1, LolC, and HI Bp82) were determined by ELISA. Circles represent the values for individual mice and the mean ± standard error of the mean (SEM) is shown with bars. Only four groups were tested for antibody to LolC in experiment 2. The dashed lines indicate the lower limit of detection (200-fold initial serum dilution). For each antigen, the mean serum IgG titers were compared to the single most relevant control group (sham for HI LVS Δ*capB* and HI Bp82, and parental LVS Δ*capB* for Bp antigens Hcp6, Hcp1, or LolC, as indicated in the graphs) using ordinary one-way ANOVA with Dunnett’s multiple comparisons test (Prism 9.3.1). *, *P*  <  0.05; **, *P*  <  0.01; ***, *P*  <  0.001; ****, *P*  <  0.0001; ns, not significant.

To evaluate the immune response of mice immunized by the ID route, we bled mice from experiment 1 (Fig. 3) and experiment 2 (Fig. 4) 1 week prior to challenge and analyzed serum IgG titers to LVS and Bp antigens (HI LVS Δ*capB*, Hcp6, Hcp1, LolC, and HI Bp82, Fig. 9). Unfortunately, we were unable to purify rHcp2 in sufficient purity and yield to be able to use it for immunologic assays. All groups immunized with the LVS Δ*capB* parental strain or an rLVS Δ*capB*/Bp vaccine, produced strong antibody titers to HI LVS Δ*capB*, whereas sham-immunized and Bp82 immunized mice did not (a few individual sham-immunized mice and Bp82 immunized mice had elevated titers to HI LVS Δ*capB* antigen, but the groups as a whole had low titers) (Fig. 9, top row). Mice immunized with rLVS Δ*capB*/Bp Hcp6-Hcp1 and rLVS Δ*capB*/Bp Hcp6-Hcp2 had anti-Hcp6 antibody titers significantly greater than LVS Δ*capB* immunized mice at various doses (Fig. 9, second row from the top). In the dose-response study (experiment 2), only the highest doses tested (8 × 10^6^ for rLVS Δ*capB*/Bp Hcp6-Hcp1 and 4 × 10^6^ for rLVS Δ*capB*/Bp Hcp6-Hcp2) showed anti-Hcp6 antibody titers significantly different from the vector control. In both experiments, the rLVS Δ*capB*/Bp Hcp6-Hcp1 vaccine showed higher anti-Hcp1 than anti-Hcp6 antibody titers; in the dose-response study (experiment 2), all doses of the rLVS Δ*capB*/Bp Hcp6-Hcp1 vaccine showed significantly elevated anti-Hcp1 titers compared with the vector control (Fig. 9, second and third rows from top). In mice immunized with escalating doses of the rLVS Δ*capB*/Bp Hcp6-Hcp1 vaccine (Fig. 9, experiment 2), serum IgG titers to Hcp6 and Hcp1 both increased with increasing vaccine dose, whereas serum IgG titers to HI LVS Δ*capB* were already maximal at the lowest dose of vaccine tested (Fig. 9, right, top three rows; Fig. S3).

Although Hcp6 is present in the three-antigen and four-antigen vaccines, these vaccines did not produce a response to Hcp6 significantly different from LVS Δ*capB*. Despite the lack of a response to Hcp6, the three-antigen vaccine, rLVS Δ*capB*/Bp Hcp6-Hcp1-LolC, produced a significant response to Hcp1, and an even stronger response to LolC (Fig. 9, right, second to fourth rows from top, rightmost vaccine).

The four-antigen vaccine, rLVS Δ*capB*/Bp LolC-Hcp6-Hcp1-Hcp2, which had fairly low expression of the fusion protein (Fig. S2), did not induce significant antibody titers to any of the three proteins tested (Fig. 9, right, second to fourth rows from top).

As expected, mice immunized with Bp82 produced high titers of IgG to HI Bp82, significantly greater than sham-immunized mice in both experiments (*P* < 0.001 and *P* < 0.0001 in experiments 1 and 2, respectively) (Fig. 9, bottom row). Of note, all groups of mice immunized with LVS Δ*capB* or rLVS Δ*capB*/Bp vaccines produced consistently elevated antibody titers to HI Bp82 in both experiments, indicating some cross-reactivity between LVS Δ*capB* and Bp (Fig. 9, bottom row); these differences were significant for all groups including the LVS Δ*capB* vector in experiment 1 (ranging between *P* < 0.05 to *P* < 0.01) and for all but the parental vector and one vaccine in experiment 2 (ranging from *P* < 0.05 to *P* < 0.0001); combining all LVS Δ*capB* and r LVS Δ*capB*/Bp vaccines, the increase in antibody to HI Bp82 was highly significant (*P* = 0.0002 and *P* < 0.0001 by two-tailed *t* test in experiments 1 and 2, respectively).

### T cell-mediated response to LVS Δ*capB* platform vaccines expressing three Bp antigens

**Fig 10:**
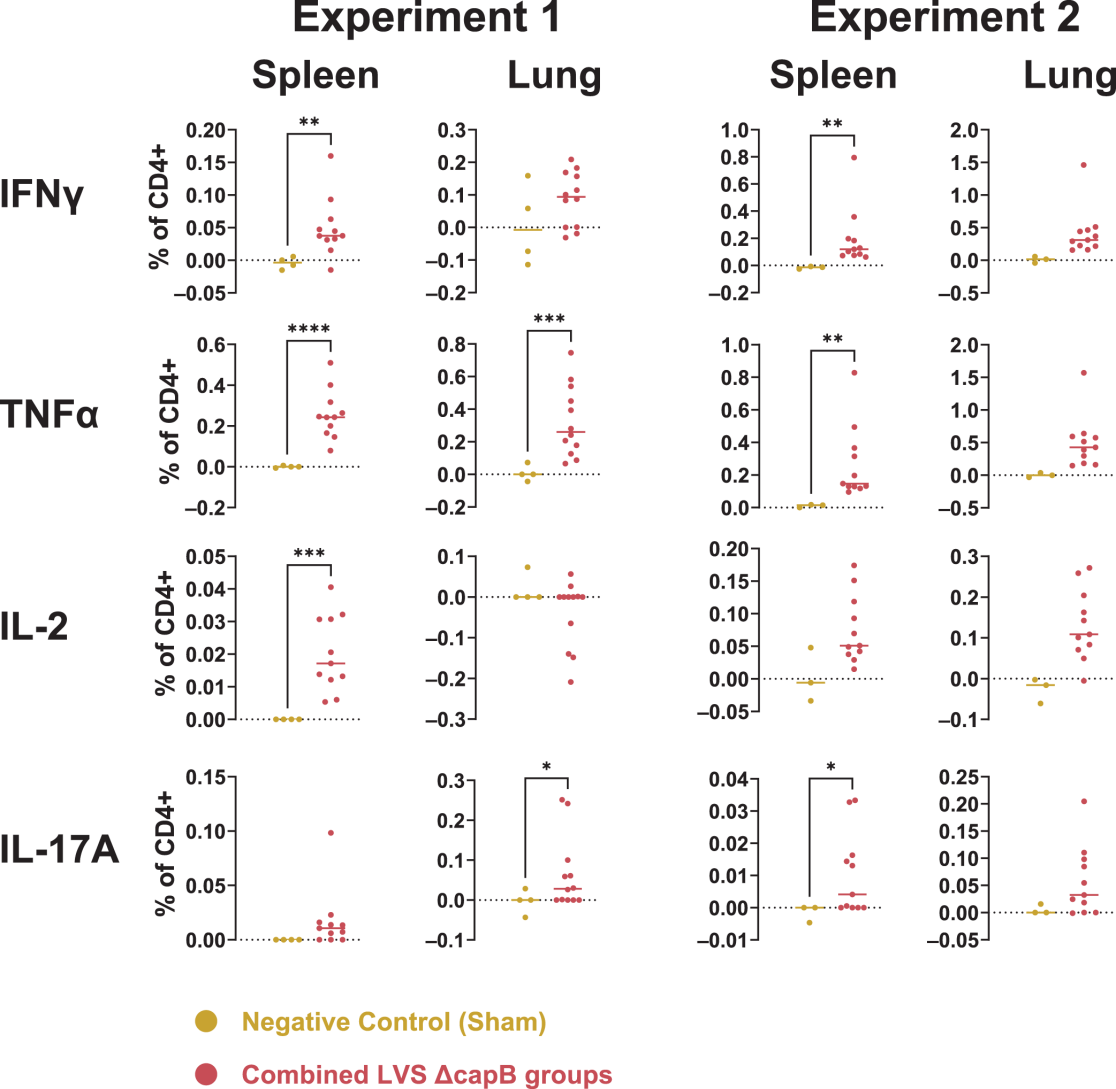
Frequency of cytokine-producing CD4+ T cells (as a percentage of total CD4+ T cells) in response to HI LVS Δ*capB* antigen (markers represent the values for individual mice and the median is shown with a horizontal bar). Mice were sham-immunized or immunized with LVS Δ*capB*, rLVS Δ*capB*/Bp Hcp6-Hcp1-Hcp2, or rLVS Δ*capB*/Bp Hcp6-Hcp1-LolC three times at weeks 0, 4, and 8 by the ID route. One week after the last immunization, splenocytes and lung cells were isolated, stimulated *in vitro* with HI LVS Δ*capB* for 6 h, and analyzed by multiparameter flow cytometry. Background numbers of cells producing cytokines without antigen stimulation were subtracted. The results from two independent experiments are shown with the three LVS Δ*capB* vaccine groups combined. The combined vaccine group was compared to the sham-vaccinated group in each graph using an unpaired, two-tailed *t* test with Welch’s correction (Prism 9.3.1). *, *P*  <  0.05; **, *P*  <  0.01; ***, *P*  <  0.001; ****, *P*  <  0.0001. Results showing the individual vaccines are presented in Fig. S4 and the flow cytometry gating strategy is shown in Fig. S5.

To further assess the response of mice immunized by the ID route, we performed two independent experiments in which mice were immunized three times, as in most of the protective efficacy studies. Each experiment consisted of four groups (sham-immunized, LVS Δ*capB*, rLVS Δ*capB*/Bp Hcp6-Hcp1-Hcp2, and rLVS Δ*capB*/Bp Hcp6-Hcp1-LolC). One week after the last immunization, spleen and lung cells were stimulated *in vitro* with LVS antigens (HI LVS Δ*capB*) and Bp antigens (rHcp1, rHcp6, rLolC, Hcp2 peptide pool, and HI Bp82) and then analyzed by multiparameter flow cytometry. Surprisingly, we did not detect any vaccine specific increased T cell responses to any of the Bp antigens (data not shown). However, spleen and lung cells from mice immunized with the parental LVS Δ*capB*, as well as the two three-antigen rLVS Δ*capB*/Bp vaccines, produced IFNγ, TNFα, IL-2, and IL-17A in response to stimulation with HI LVS Δ*capB* (Fig. S4; Fig. 10); when the LVS Δ*capB* groups were combined, these differences were statistically significant in half of the comparisons (Fig. 10).

## DISCUSSION

In this study, we demonstrate that LVS Δ*capB*-vectored vaccines expressing Bp antigens are highly protective against lethal pneumonic melioidosis in a highly sensitive animal model. We chose the IN route to challenge animals with Bp since respiratory challenge (IN or aerosol): (i) is generally regarded as being more difficult to protect against than IP challenge, the route often used in earlier Bp studies (39), (ii) provides the most relevant model for an intentional bioterrorist attack, and (iii) models natural disease acquired via inhalation. We used BALB/c mice in our studies as this strain is more sensitive to Bp than the other commonly used mouse strain, C57BL/6; BALB/c mice are reported to be 10–100× more sensitive to Bp challenge than C57BL/6 mice depending on the strain of Bp, although smaller differences have also been reported (18–20, 38, 40–42). BALB/c mice are also seemingly harder to protect by vaccination than C57BL/6 mice. For example, Silva et al., using Bp82 as a vaccine, obtained 60% survival in BALB/c mice vs 100% protection in C57BL/6 mice at 60 days post-challenge with 5× LD50 of Bp 1026b delivered by the IN route (38). Similarly, Zimmerman et al. (42), using an attenuated *B. mallei batA* mutant as a melioidosis vaccine, obtained 67% survival at 55 days against intratracheal (IT) aerosol challenge with 5× LD50 Bp 1026b (eight independent experiments) vs 100% survival (and 100% sterile immunity) against IT aerosol challenge with 5× LD50 Bp 1026b in C57BL/6 mice (one experiment with 6 mice per group). Bp outer membrane vesicles also apparently protect C57BL/6 mice (43) better than BALB/c mice (44), but the two relevant studies cited have a number of other differences, which might account for the difference in efficacy. Thus, in challenging by the respiratory route and using the BALB/c mouse model of lethal pneumonic melioidosis, we set a high bar for assessing the efficacy of our rLVS Δ*capB*/Bp vaccines.

Besides ours, only a handful of candidate melioidosis vaccines have demonstrated substantial efficacy against lethal respiratory challenge in the BALB/c mouse model, and some of these have done so under less stringent conditions than we employed (Table S12). These vaccines include Bp82 (Bp 1026b Δ*purM*) (38) (60% survival at 60 days) and *B. mallei batA (*42) (67% survival at 55 days), both single-deletional mutant strains; heat-inactivated Bp in CLDC (cationic liposome-DNA complex) adjuvant (100% survival at 40 days) (45); *B. mallei* protein BopA in CLDC adjuvant (60% survival at 55 days) (14); Bp-derived outer membrane vesicles (Bp OMVs) (53% survival at 14 days) (44); and parainfluenza virus 5 expressing *B. mallei* BatA (PIV5-BatA, 60% survival at 42 days) (46). Live attenuated Bp and *B. mallei batA* mutants are among the vaccines with the highest efficacies but, as single deletional mutants, they are probably unsuitable for human use because of concern for possible reversion to virulence and establishment of latent infection. The two studies using CLDC adjuvant had very short immunization-challenge intervals of only 2 weeks following IN vaccination (14, 45), which may not be long enough for non-specific innate immune responses to wane. The study using Bp OMVs only monitored survival for 14 days (44), so it is likely that survival would be lower at longer times post-challenge such as the 42 days we used in our experiments.

Other vaccines have shown significant protection against lethal respiratory Bp challenge in the more resistant C57BL/6 mouse strain, including outer membrane vesicles (43) and glycoconjugates (47–52) (Table S13). CPS-CRM197 + Hcp1, among the best and most studied of these vaccines, yielded long-term protection in C57BL/6 mice of 100% survival (at 35 days) (48), 80% survival (at 60 days) (47), and 60–80% survival (at 30 days) (49). CPS-CRM197 + Hcp1 + AhpC^C57G^ and CPS-CRM197 + AhpC^C57G^ vaccines have also produced moderate to good protective efficacy (35–80% survival measured at 30, 35, or 60 days) (47, 49, 51). Gold nanoparticle glycoconjugate vaccines have also performed very well in C57BL/6 mice (90% and 100% survival at 35 days for the best vaccines) (50, 52).

In our study, we adapted the Electra cloning system to facilitate the rapid construction of many rLVS Δ*capB*/Bp strains expressing antigens of interest. Using this system, we were able to readily build and test different expression plasmids in which we varied the codon usage of the selected antigen genes, the linker joining CDSs, the order of CDSs, the presence or absence of a tag for detecting the expressed antigen, and the tag’s location (N-terminus or C-terminus), in order to select well-expressed antigens. Many of the findings from testing many different expression constructs were not obvious a priori (e.g., expression of *hcp6* codon-optimized specifically for *L. monocytogenes* was better than the version codon-optimized for LVS), indicating the value of the approach. This system should prove useful for constructing rLVS Δ*capB* vaccines targeting other pathogens as well.

In our studies, we used Bp82 (Bp 1026b Δ*purM*), as a positive control vaccine in all of our experiments. As noted above, this vaccine, albeit a single-deletional mutant and hence unsuitable for human use, has demonstrated strong protective immunity against pneumonic melioidosis. In our six experiments in BALB/c mice, Bp82 delivered by the ID route in a homologous boosting regimen (1 × 10^6^ CFU administered at weeks 0, 4, and 8) yielded protection ranging from 25% to 100% survival at 42 days post-challenge (median survival 35%, mean survival 49%). Survival with sterile immunity for Bp82 vaccinated mice ranged from 0% to 50% (median 19%, mean 21%). Our results are roughly comparable to the original Bp82 vaccine study which obtained 60% survival in BALB/c mice challenged by the IN route and reported that surviving mice had detectable Bp in their organs at the end of the experiment (38).

Administration of our vaccines by the IN route was superior to administration by the ID route, especially at high challenge doses. When rLVS Δ*capB*/Bp vaccines were administered by the ID route at doses of ≥1 × 10^6^ CFU in a homologous boosting regimen (week 0, 4, and 8), they showed good protective efficacy (median survival 63% at 42 days; experiments 1, 2, 4, and 5) in several experiments in which animals were given a relatively low-dose lethal respiratory challenge. However, the vaccine showed little to no efficacy (median survival 3% at 42 days; experiments 3, 5, and 6) in experiments in which the animals were challenged with a relatively high lethal dose (Fig. 8). In contrast, when rLVS Δ*capB*/Bp vaccines were administered by the IN route, they were highly efficacious against both relatively low and high lethal challenge doses (median survival 75% at 42 days; experiments 3, 4, and 6).

Of note, even a single IN dose of the rLVS Δ*capB*/Bp vaccine was highly effective. In the study comparing one, two, or three doses, one or two doses were as effective as three doses (median survival 87% at 42 days, experiment 3). Thus, a single dose is all that appears to be needed to achieve high-level protection when the vaccine is administered IN. We do not know if this will hold true for ID immunization as we have not yet tried immunizing by the ID route with fewer than three doses.

Also of note, our rLVS Δ*capB*/Bp vaccines were efficacious even when the immunization-challenge interval was extended to 12 weeks. While we used an immunization-challenge interval of 4 or 6 weeks in most of our studies, in one study, we extended this to 12 weeks and still obtained good protection by the ID and, in particular, the IN route (experiment 4). To our knowledge, this is the longest immunization-challenge interval for which a melioidosis vaccine has demonstrated efficacy. The vaccine studies by others cited above used immunization-challenge intervals ranging from two to six weeks.

In our first experiment, the LVS Δ*capB* parental vector had no protective efficacy (1 × 10^6^ CFU dose), whereas the rLVS Δ*capB*/Bp vaccines had good protective efficacy. However, in subsequent experiments using a higher immunization dose (2 × 10^6^ CFU), the LVS Δ*capB* parental vector was protective against Bp challenge. Interestingly, previous studies also found that a live tularemia vaccine (GTV) provided some protective efficacy against melioidosis (53, 54).

Although it is possible that non-specific immunity may be contributing to protection against Bp elicited by the parental vector, akin to the heterologous non-specific protection against other pathogens described for BCG, influenza virus, and intranasally administered *E. coli* heat-labile toxin, among others (55–57), there is evidence against this being the primary factor. First, LVS Δ*capB* administered by the ID and IN routes, is rapidly cleared from the spleen, lung, liver, and lymph nodes of BALB/c mice within 2–3 weeks post-vaccination (see Fig. S4 and S5 in reference 9); hence, the innate immune response is likely to have subsided substantially by the time of challenge at ≥4 weeks. In the same study, rLVS Δ*capB* expressing anthrax and plague antigens were also cleared within 2–3 weeks. Thus, for all experiments in the current study, our vaccines are expected to have been cleared well before challenge. In experiment 4 (Fig. 6), we increased the immunization-challenge interval to 12 weeks to evaluate long-term protection, which additionally should have further minimized the likelihood of non-specific immunity impacting the result, yet we still obtained very potent protection when vaccines were administered by the IN route. Thus, short-lived innate immune responses would not seem to be a factor, especially in this case. However, as trained immunity can be rather long lived in some instances (58), this result alone cannot rule out non-specific immunity induced by the vector as contributing to the protection against Bp. A second argument against non-specific immunity playing a major role in protection in our studies is that the LVS Δ*capB* parental vector is unprotective, or at best very weakly protective, against two other pathogens. In the case of SARS-CoV-2, LVS Δ*capB* administered by the SQ, ID, IN, or PO routes was not at all or very poorly protective in ameliorating weight loss, lung pathology, loss of alveolar air space, and viral load in the oropharynx and lungs after SARS-CoV-2 respiratory challenge in hamsters (4 or 5 weeks after the last immunization), whereas an rLVS Δ*capB* vaccine expressing SARS-CoV-2 membrane and nucleocapsid proteins was highly protective in all these respects (7, 8). In the case of *Yersinia pestis*, LVS Δ*capB* administered by the ID route had no protective efficacy against either weight loss or survival (0% survival) after *Y. pestis* respiratory challenge in BALB/c mice 4 weeks after the last immunization, whereas rLVS Δ*capB* expressing *Y. pestis* immunoprotective proteins fully protected against weight loss and survival (100% survival) (Qingmei Jia, Richard A. Bowen, and Marcus A. Horwitz, unpublished data). While these arguments do not fully rule out LVS Δ*capB* eliciting a non-specific immune response that is effective against Bp, we believe cross-protective immunity, as reflected by elevated anti-HI Bp82 antibody titers (see below), is playing the major role in protection of the vector against Bp respiratory challenge.

As observed in previous studies utilizing the LVS Δ*capB* vaccine platform, we obtained robust humoral immunity to LVS Δ*capB* antigens (HI LVS Δ*capB*) in all groups of mice immunized with LVS Δ*capB* or rLVS Δ*capB*/Bp vaccines (7–11). This response was already maximal at the lowest dose of vaccine tested (1 × 10^6^ CFU). We also found that mice immunized with LVS Δ*capB* or rLVS Δ*capB*/Bp vaccines had significantly elevated antibody titers to HI Bp82 vs sham-immunized mice (*P* = 0.0002 and *P* < 0.0001 by two-tailed *t* test in experiments 1 and 2, respectively, for combined vaccines), demonstrating cross-reactivity between LVS Δ*capB* and Bp. Like the response to HI LVS Δ*capB*, the response to HI Bp82 also appeared to be maximal at the lowest dose of vaccine tested (1 × 10^6^ CFU). For the heterologously expressed Bp antigens, we observed antibody responses to Hcp6, Hcp1, and LolC from various rLVS Δ*capB*/Bp vaccines. In contrast to HI LVS Δ*capB* and HI Bp82 antigens, we observed antibody titers to Hcp6 and Hcp1 that increased with increasing doses of vaccine. Interestingly, for the three-antigen vaccine, rLVS ΔcapB/Bp Hcp6-Hcp1-LolC, the strongest antibody response was to LolC, followed by Hcp1, and with no detectable response to Hcp6.

While we found that T cells from mice immunized with LVS Δ*capB* or rLVS Δ*capB*/Bp vaccines expressed statistically significantly elevated levels of cytokines in response to *in vitro* stimulation with HI LVS Δ*capB*, we did not detect statistically significant increases in T cells expressing cytokines in response to *in vitro* stimulation with Bp antigens (rHcp6, rHcp1, rLolC, or Hcp2 peptide pool). Hence, humoral rather than cell-mediated immunity appears to dominate the immune response to individual Bp antigens expressed by our rLVS Δ*capB*/Bp vaccines.

We encountered some variation in the LD_50_ for Bp 1026b used as the challenge strain in all our experiments, spanning several years. Based on an initial calculated LD_50_ of 450 CFU, our first four experiments used IN challenge doses of 5×, 4×, 6×, and 3.2× LD_50_ and all four experiments had 100% rapid lethality in the sham-vaccinated mice. In contrast, for experiment 5, the low-dose and medium-dose challenges, which should have been 3.4× and 4.2× LD_50_ challenge doses based on a 450 CFU LD_50_, did not result in 100% lethality. We obtained a revised LD_50_ of 1,200 CFU for this experiment. Finally, in view of these results in experiment 5, we performed another LD_50_ study immediately prior to initiating experiment 6, and we obtained an LD_50_ of 2,800 CFU. We are uncertain as to why the LD_50_ varied among these experiments over time.

While our studies were designed to assess the efficacy of our rLVS Δ*capB*/Bp vaccines against pneumonic melioidosis, modeling natural disease acquired via inhalation or disease acquired via a bioterrorist attack employing aerosolization of Bp, in future studies, we intend to assess the efficacy of our vaccines against subcutaneous challenge, to model the presumed primary means of naturally acquired infection.

This work extends our laboratory’s previous studies, which used the LVS Δ*capB* vaccine platform to develop vaccines against the three Tier 1 select agents causing anthrax, plague, and tularemia (9–11), to a fourth Tier 1 select agent, that causing melioidosis. We believe that a single vector platform approach targeting multiple diseases has significant advantages in terms of vaccine cost, ease of administration, regulatory approval, and patient acceptability. Cost is an especially important consideration in a melioidosis vaccine targeted for endemic regions (4, 59), many of which are in relatively low-income countries in Southeast Asia, making our rLVS Δ*capB*/Bp vaccines attractive candidates for further development.

In addition to its use as a platform for vaccines against Tier 1 select agents, the LVS Δ*capB* vector platform has been used successfully to develop a low-cost oral universal vaccine against COVID-19 (7, 8).

In summary, rLVS Δ*capB*/Bp vaccines expressing multiple Bp antigens, especially three-antigen vaccines expressing Hcp6-Hcp1-Hcp2 or Hcp6-Hcp1-LolC, induce potent protective immunity against lethal respiratory challenge with highly virulent Bp in the highly sensitive BALB/c mouse model. The vaccines are most efficacious when administered by the IN route and even a single IN dose is highly effective. Moreover, the rLVS Δ*capB*/Bp vaccines induce long-lasting protective immunity. Thus, the three-antigen rLVS Δ*capB*/Bp vaccines show great promise as a safe, potent, low-cost vaccine against melioidosis, a major neglected infectious disease for which no licensed vaccine currently exists.

## Data Availability

Data will be made available on request.

## SUPPLEMENTAL MATERIAL

Supplemental material mbio.00186-24-s0001.pdf Figures S1-S5 and Tables S1-S13.

ASM does not own the copyrights to Supplemental Material that may be linked to, or accessed through, an article. The authors have granted ASM a non-exclusive, world-wide license to publish the Supplemental Material files. Please contact the corresponding author directly for reuse.
