# Supplementary material for: LVS ΔcapB-vectored multiantigenic melioidosis vaccines protect against lethal respiratory Burkholderia pseudomallei challenge in highly sensitive BALB/c mice
Source: mBio. 2024 Mar 21;15(4):e00186-24. doi: 10.1128/mbio.00186-24 (PMC11005352; doi:10.1128/mbio.00186-24)
Supplement: Supplemental material — Figures S1-S5 and Tables S1-S13. [file mbio.00186-24-s0001.pdf]

Supplementary Information

Supplementary Figures S1-S5

Supplementary Tables S1-S13

LVS Δ*capB*-vectored multiantigenic melioidosis vaccines protect against lethal respiratory *Burkholderia pseudomallei* challenge in highly sensitive BALB/c mice

***Authors*:**

Michael V. Tullius[a], Richard A. Bowen[b], Peter S. Back[a], Saša Masleša-Galić[a], Susana Nava[a], and Marcus A. Horwitz[a,#]

***Author Affiliation*:**

[a]Division of Infectious Diseases, Department of Medicine, 32-150 Center for Health Sciences, School of Medicine, University of California – Los Angeles, 10833 Le Conte Avenue, Los Angeles, CA 90095-1688

[b]Department of Biomedical Sciences, Colorado State University, Fort Collins, CO, 80523

Supplementary Figure S1

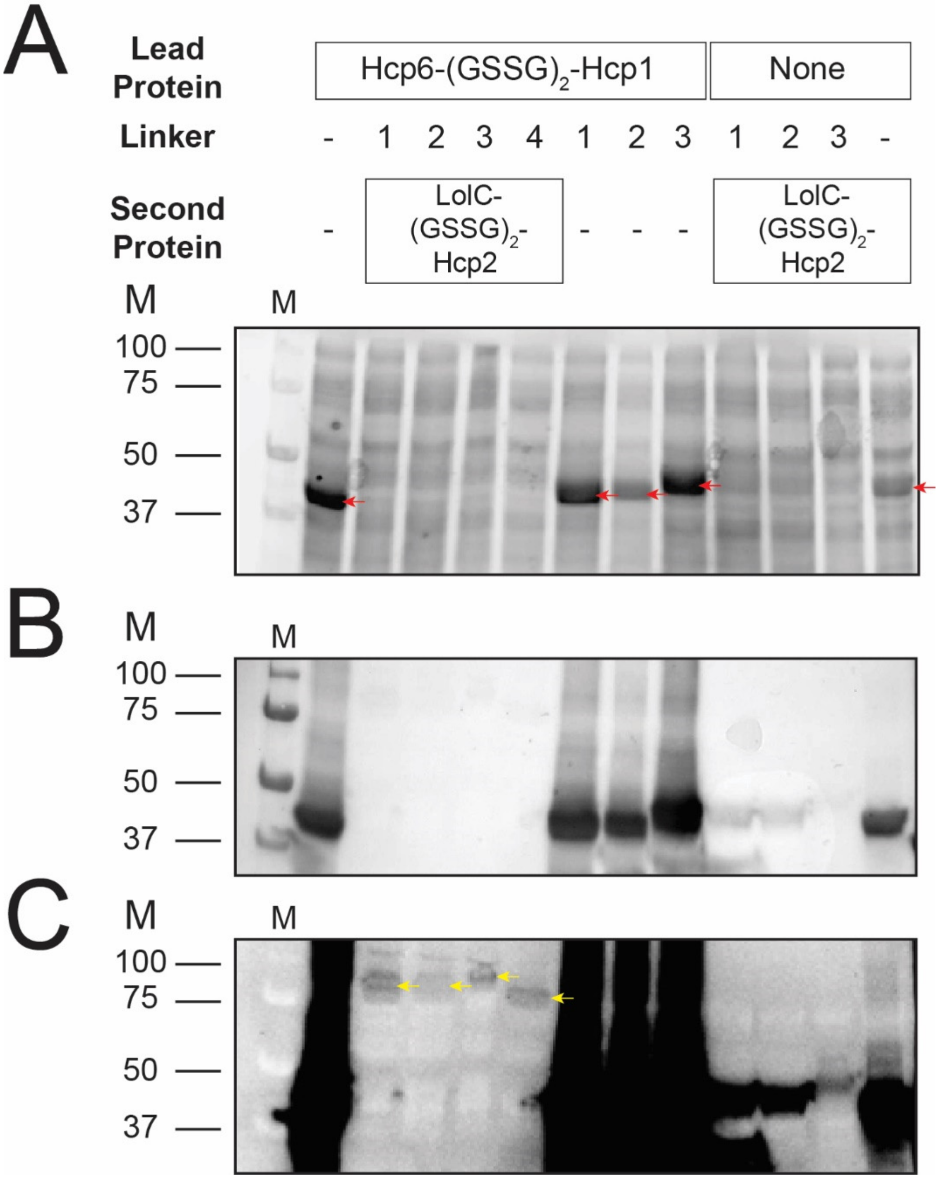

Supplementary Figure S1. Expression of four-antigen fusion proteins by rLVS Δ*capB*. Cleared cell lysate from rLVS Δ*capB* strains expressing four-antigen fusion proteins with a C-terminal FLAG tag were analyzed by SDS-PAGE (A) and Western blotting (B and C) using anti-FLAG monoclonal antibody (the amount of lysate loaded per lane is equivalent to the amount of cells from 100 µL of overnight culture). The blot in C is the same blot as in B, but the brightness and contrast adjusted in order to visualize the very poorly expressed four-antigen fusion proteins (yellow arrows). To construct the four-antigen fusion proteins, we joined LolC-(GSSG)$_2$-Hcp2 to the C-terminus of Hcp6-(GSSG)$_2$-Hcp1 using one of four linkers (indicated above the gel): (1) GSAGSAAGSGEF; (2) A(EAAAK)$_3$A; (3) (AP)$_{10}$; (4) direct linkage (single alanine residue). Red arrows indicate the position of recombinant fusion proteins when apparent. M, molecular mass markers in kDa.

Supplementary Figure S2

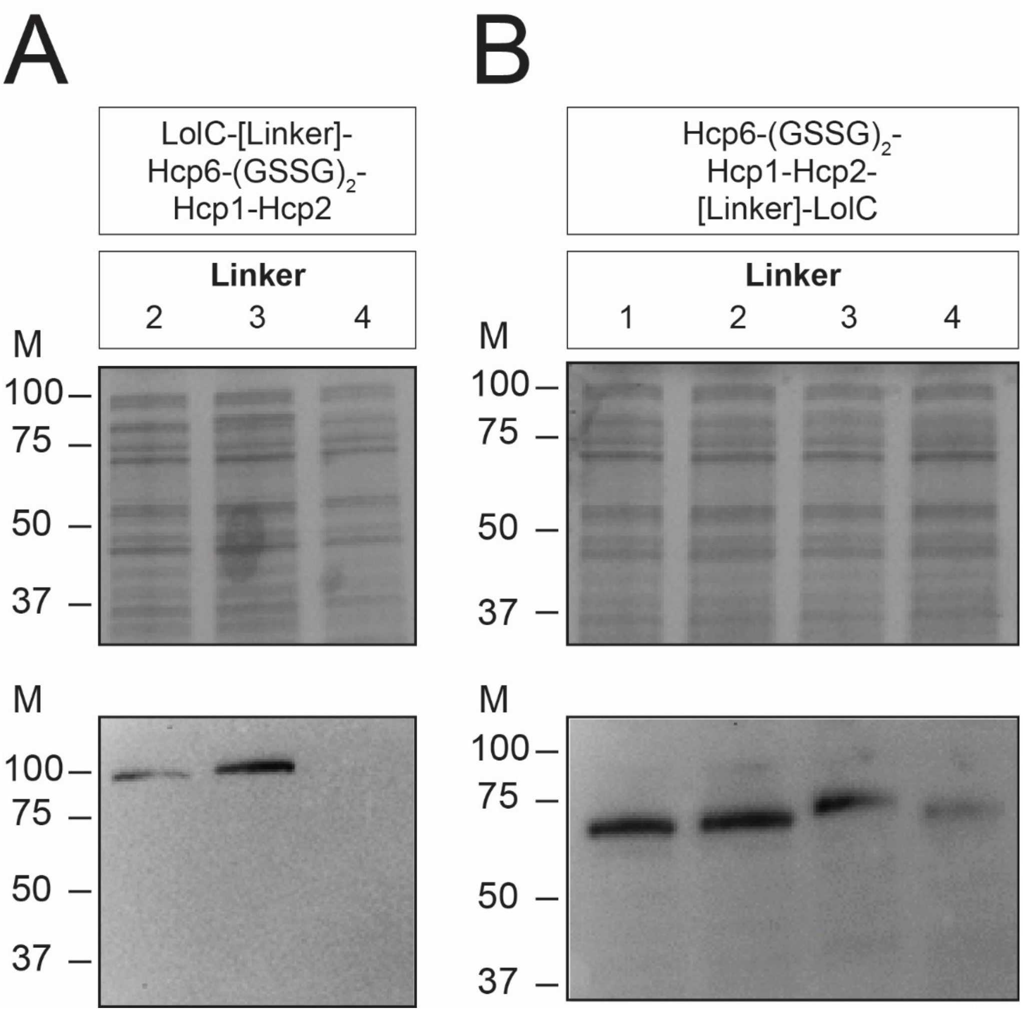

Supplementary Figure S2. Expression of four-antigen fusion proteins by rLVS Δ*capB*. Cleared cell lysate from rLVS Δ*capB* strains expressing four-antigen fusion proteins with a C-terminal FLAG tag were analyzed by SDS-PAGE (upper images) and Western blotting (lower images) using anti-FLAG monoclonal antibody (the amount of lysate loaded per lane is equivalent to the amount of cells from 25 µL of overnight culture). To construct the four-antigen fusion proteins, LolC was joined to either the N-terminus (A) or C-terminus (B) of Hcp6-(GSSG)$_2$-Hcp1-Hcp2 using one of four linkers (indicated above the gels): (1) GSAGSAAGSGEF; (2) A(EAAAK)$_3$A; (3) (AP)$_{10}$; (4) direct linkage (single A residue). M, molecular mass markers in kDa.

Supplementary Figure S3

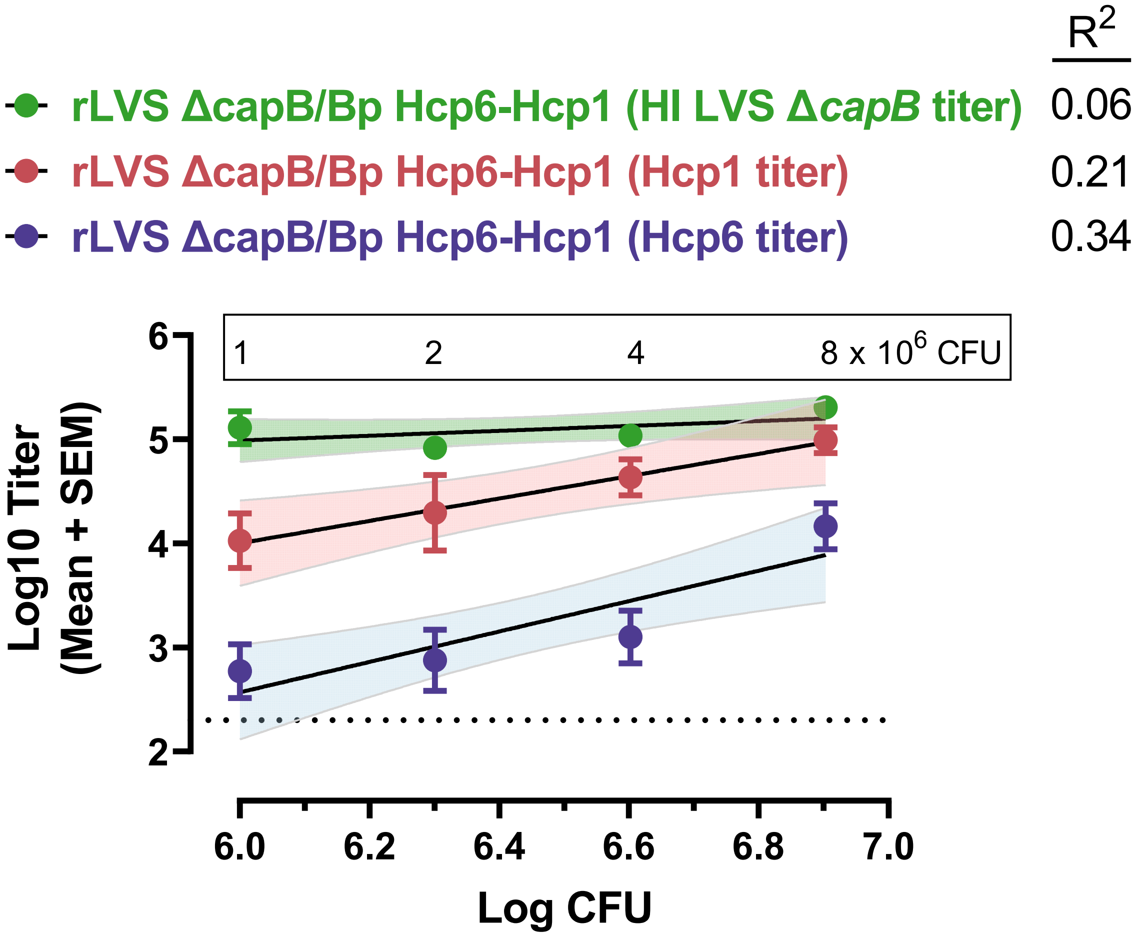

Supplementary Figure S3. Dose response of antibody titers and Log CFU of the rLVS Δ*capB*/Bp Hcp6-Hcp1 vaccine which was given at 1, 2, 4, and 8 x 10^6 CFU. The data for the three different antigens (HI LVS Δ*capB*, Hcp6, and Hcp1) from Experiment 2 (**Fig. 9**) were fit to a straight line using non-linear regression (Prism 9.3.1) and the R^2 value is shown. Shaded areas represent the 95% confidence interval. The dashed line indicates the lower limit of detection (200-fold initial serum dilution).

Supplementary Figure S4

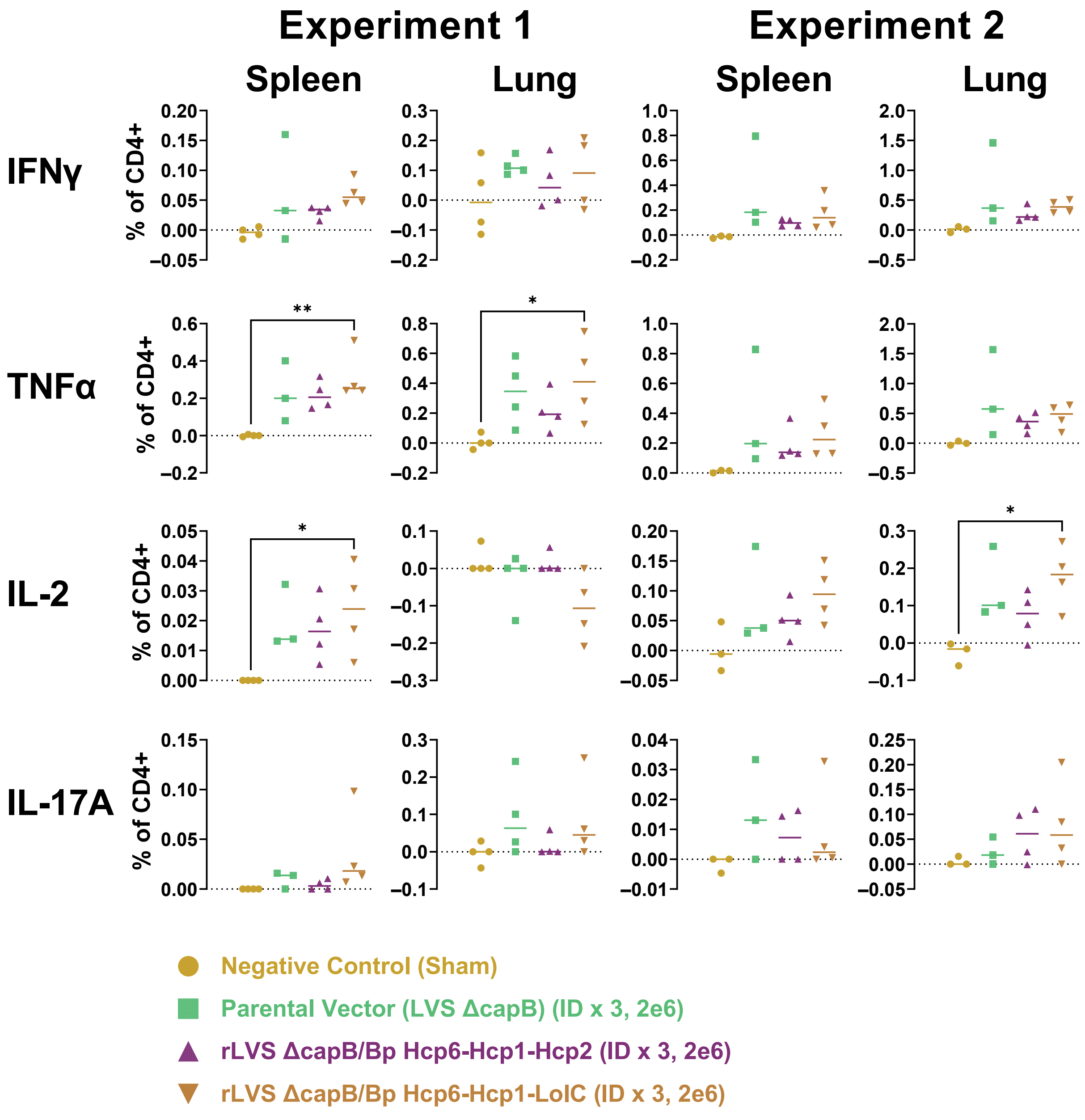

Supplementary Figure S4. Frequency of cytokine-producing CD4+ T cells (as a percentage of total CD4+ T cells) in response to HI LVS Δ*capB* antigen (markers represent the values for individual mice and the median is shown with a horizontal bar). Mice were sham-immunized or immunized with LVS Δ*capB*, rLVS Δ*capB*/Bp Hcp6-Hcp1-Hcp2, or rLVS Δ*capB*/Bp Hcp6-Hcp1-LolC three times at weeks 0, 4, and 8 by the ID route. One week after the last immunization, splenocytes and lung cells were isolated, stimulated *in vitro* with HI LVS Δ*capB* for 6 h, and analyzed by multiparameter flow cytometry. Background numbers of cells producing cytokines without antigen stimulation were subtracted. The results from two independent experiments are shown. For each graph, the mean percentage of cytokine expressing cells from each group was compared with all other groups using ordinary One-Way ANOVA with Dunnett's multiple comparisons test (Prism 9.3.1). *, P < 0.05; **, *P* < 0.01. Results showing the three LVS Δ*capB* vaccine groups combined are presented in **Fig. 10** and the flow cytometry gating strategy is shown in **Supplementary Fig. S5**.

Supplementary Figure S5

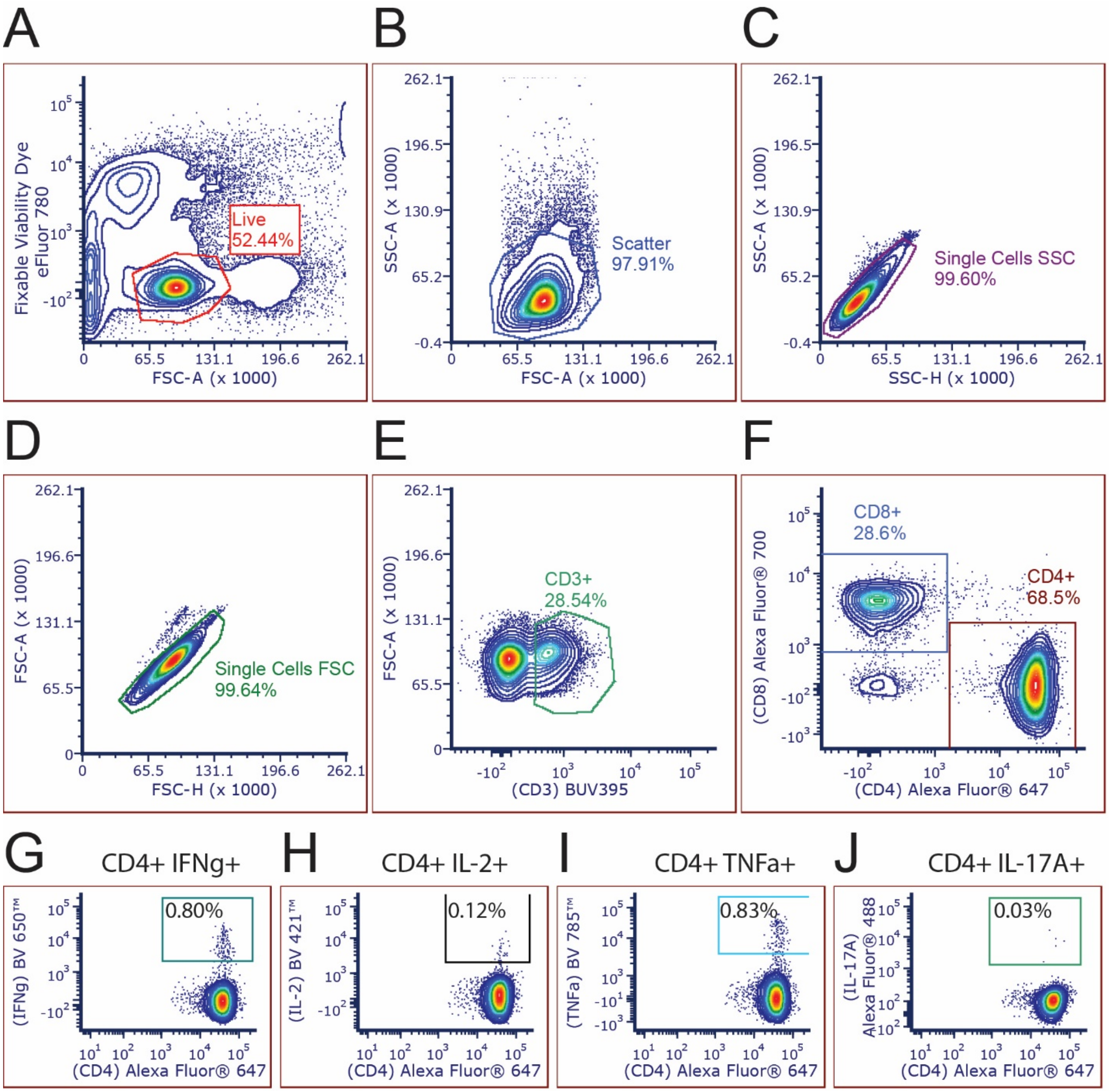

Supplementary Figure S5. Flow cytometry gating strategy used to determine the frequency of cytokine-producing CD4+ T cells for the data presented in **Fig. 10** and **Supplementary Fig. S4**. **A** through **F**: sequential gates used to identify live, CD3+ CD4+ T cells; **A**. Live cells. **B**. Scatter. **C**. Single cells (SSC area vs. height). **D**. Single cells (FSC area vs. height). **E**. CD3+. **F**. CD4+ and CD8+ T cells. **G** through **J**: gates used to identify cytokine expressing CD4+ T cells; **G**. IFNγ+. **H**. IL-2+. **I**. TNFα+. **J**. IL-17A+. The data shown are from splenocytes from a single mouse immunized with LVS Δ*capB* from Experiment 2, stimulated *in vitro* with HI LVS Δ*capB* antigen.

Supplementary Table S1. Bacterial Strains

Strain	Description	Plasmid	Antibiotic Resistance Markers	Included in Protective Efficacy Experiments	Included in Immunology Experiments	Reference or Source
E. coli DH5a	General cloning strain		None			Gibco BRL
E. cloni ® 10G	*Escherichia coli* cloning strain optimized for high efficiency transformation; also used for expression of recombinant proteins		Str^r			Lucigen
LVS Δ*capB*	*F. tularensis* live vaccine strain (LVS) with unmarked deletion of *capB*		None*	1, 2, 3, 4, 5	1, 2	Jia, Q. et al. Infect Immun 78, 4341-4355, doi:10.1128/IAI.00192-10 (2010).
Bp82	*B. pseudomallei* 1026b Δ*purM* (adenine and thiamine auxotroph)<br><br>excluded from select-agent regulations		None**	1, 2, 3, 4, 5, 6		Provided by Herbert P. Schweizer. Propst, K. L. et al. Infect Immun 78, 3136-3143, doi:10.1128/IAI.01313-09 (2010).
Bp 1026b	*B. pseudomallei* 1026b virulent strain used for all challenges		None**	1, 2, 3, 4, 5, 6		Provided by Herbert P. Schweizer. See reference above.

* LVS is naturally resistant to ampicillin

** Bp is naturally resistant to several antibiotics

Supplementary Table S1 (continued). Bacterial Strains

Strain	Description	Plasmid	Antibiotic Resistance Markers	Included in Protective Efficacy Experiments	Included in Immunology Experiments	Reference or Source
rLVS Δ*capB*/Bp Hcp6-Hcp1	rLVS Δ*capB* vaccine strain expressing Bp antigens as a fusion protein	pFNL-bfr-**Hcp6 (coLm)**-(GSSG)2-**Hcp1 (coLVS)**-C8H-3F	Km^r	1, 2		This study
rLVS Δ*capB*/Bp Hcp6-Hcp2	rLVS Δ*capB* vaccine strain expressing Bp antigens as a fusion protein	pFNL-bfr-**Hcp6 (coLm)**-(GSSG)2-**Hcp2 (coLVS)**-C8H-3F	Km^r	1, 2		This study
rLVS Δ*capB*/Bp LolC-Hcp1	rLVS Δ*capB* vaccine strain expressing Bp antigens as a fusion protein	pFNL-bfr-**LolC (coLVS)**-(GSSG)2-**Hcp1 (coLVS)**-C8H-3F	Km^r	1		This study
rLVS Δ*capB*/Bp LolC-Hcp2	rLVS Δ*capB* vaccine strain expressing Bp antigens as a fusion protein	pFNL-bfr-**LolC (coLVS)**-(GSSG)2-**Hcp2 (coLVS)**-C8H-3F	Km^r	1		This study

Supplementary Table S1 (continued). Bacterial Strains

**Strain**	**Description**	**Plasmid**	**Antibiotic Resistance Markers**	**Included in Protective Efficacy Experiments**	**Included in Immunology Experiments**	**Reference or Source**
rLVS Δ*capB*/Bp Hcp6-Hcp1-LolC	rLVS Δ*capB* vaccine strain expressing Bp antigens as a fusion protein	pFNLdA-bfr-**Hcp6**-(GSSG)2-**Hcp1**-GSAGSAAGSGEF-**LolC**-C8H-3F	Km^r	2, 3, 4	1, 2	This study
rLVS Δ*capB*/Bp Hcp6-Hcp1-Hcp2	rLVS Δ*capB* vaccine strain expressing Bp antigens as a fusion protein	pFNLdA-bfr-(5'gca)-**Hcp6**-(GSSG)2-**Hcp1-Hcp2**-C8H-3F	Km^r	2, 3, 4, 5, 6	1, 2	This study
rLVS Δ*capB*/Bp LolC-Hcp6-Hcp1-Hcp2	rLVS Δ*capB* vaccine strain expressing Bp antigens as a fusion protein	pFNLdA-bfr-**LolC**-(AP)10-**Hcp6**-(GSSG)2-**Hcp1-Hcp2**-C8H-3F	Km^r	2		This study

Supplementary Table S2. Oligonucleotides

Purpose	Name	Sequence (5'→ 3')
**Construction of Electra compatible pFNL plasmids**		
**Purpose**	**Name**	**Sequence (5'→ 3')**
Site-Directed Mutagenesis to remove SapI sites from pFNL/pbfr-SD-iglA	pFNL-ΔSapI-1	GGATACTTTTCATGAAG**t**TCTTCTATTTTTCCCCAG
	pFNL-ΔSapI-2	GATCTGGACGAAGA**a**CATCAGGGGCTCG
	pFNL-ΔSapI-3	GATATTGCTGAAGA**a**CTTGGCGGCGAATGG
	pFNL-ΔSapI-4	CGAGGAAGCGGAA**a**AGCGCCCAATAC
Amplification of pFNL plasmids to remove the ampicillin resistance gene via IVA	pFNL-delAmp-F	**cttgacgagttcttctgaa**CTGTCAGACCAAGTTTACTCATATATACT
	pFNL-delAmp-R	**TTCAGAAGAACTCGTCAAG**AAGGC
**Overexpression of Bp antigens in *E. coli***		
**Purpose**	**Name**	**Sequence (5'→ 3')**
Amplification of Bp antigen genes for cloning into pRham N-His SUMO Kan expression vector (Lucigen)	hcp1(coLVS)-SumoF4	**CGCGAACAGATTGGAGGT**ggatccCTAGCGGGAATATATCTTAAGGTGAAGGGG
	hcp1(coLVS)-Sumo-R	**GTGGCGGCCGCTCTATTA**ATTAGTCCAATTTGCAGCACCTGCAATGCC
	hcp2(coLVS)-Sumo-F	**CGCGAACAGATTGGAGGT**GCGAATGCTTTGGTAGACTACTTTCTTCAG
	hcp2(coLVS)-Sumo-R	**GTGGCGGCCGCTCTATTA**AATTGGAGCATTTTGTTTCAGATCATATCC
	hcp6(coLVS)-SumoF4	**CGCGAACAGATTGGAGGT**ggatccTTACACATGCATTTGAAATTTGGATCTCCTGCAA
	hcp6(coLVS)-Sumo-R	**GTGGCGGCCGCTCTATTA**AACTGCATAAGTCTTATCATTTTTCGTAAGACTC
	lolC(coLVS)-Sumo-F	**CGCGAACAGATTGGAGGT**AATGGATTTCAAAAAGAAGTTAGAGATAGAATGTTA
	lolC(coLVS)-Sumo-R	**GTGGCGGCCGCTCTATTA**TCGCTTCTCAATCTGCACAGCACTAAACCA
**Construction of peptide linkers for fusing Bp antigens**		
**Purpose**	**Name**	**Sequence (5'→ 3')**
Construction of protein linker ORFs by annealing complementary oligonucleotides	GSAGSAAGSGEF-Top	**gca**GGTTCCGCTGGGTCTGCTGCAGGAAGTGGTGAGTTC
	GSAGSAAGSGEF-Bottom	**aga**GAACTCACCACTTCCTGCAGCAGACCCAGCGGAACC
	(AP)10-Top	**gca**CCTGCGCCCGCGCCTGCACCAGCTCCTGCCCCTGCTCCTGCTCCAGCGCCGGCTCCT
	(AP)10-Bottom	**aga**AGGAGCCGGCGCTGGAGCAGGAGCAGGGGCAGGAGCTGGTGCAGGCGCGGGCGCAGG
	A(EAAAK)3A-Top	**gca**GAGGCGGCTGCTAAGGAAGCTGCTGCTAAAGAAGCGGCTGCCAAGGCA
	A(EAAAK)3A-Bottom	**aga**TGCCTTGGCAGCCGCTTCTTTAGCAGCAGCTTCCTTAGCAGCCGCCTC

Supplementary Table S2 (continued). Oligonucleotides

Construction of hcp6-hcp1, hcp6-hcp2, lolC-hcp1, and lolC-hcp2 fusions joined with a GSSG-GSSG linker		
**Purpose**	**Name**	**Sequence (5'→ 3')**
Construction of *hcp6* and *lolC* derivatives with GSSG-GSSG linker by Gibson Assembly	pM264-FP	TGTTATCAGTATTTATTATGCATTTAGAAT
	hcp6_coLm-L-R	ACCTGAAGATCCTCCAGAACTACCTACAGCGTAGGTCTTATCATTTTTC
	lolC_coLm-L-R	ACCTGAAGATCCTCCAGAACTACCTCTTTTTTCAATTTGCACAGCAGAAAAC
	lolC_coLVS-L-R	ACCTGAAGATCCTCCAGAACTACCTCGCTTCTCAATCTGCACAGCAC
	Linker-3gca-R	AATTTTTCATGATTTGTGTCGCCCTTTTGACGGCTCTTCTtgcACCTGAAGATCCTCCAGAACTACC
Construction of *hcp1* and *hcp2* derivatives with 5'GCA SapI overhang by Gibson Assembly	pM264-RP	TCACAAAGCAAATAAATTTTTCATGA
	hcp1_nat-5gca-F	AAATTTTGTGTCGCCCTTCGCTGAAGCTCTTCTgcaCTGGCCGGAATATATCTCAAGGTC
	hcp1_coLm-5gca-F	AAATTTTGTGTCGCCCTTCGCTGAAGCTCTTCTgcaCTAGCTGGGATTTACTTAAAGGTT
	hcp1_coLVS-5gca-F	TTTTGTGTCGCCCTTCGCTGAAGCTCTTCTgcaCTAGCGGGAATATATCTTAAGGTGAAG
	hcp2_nat-5gca-F	TTTTGTGTCGCCCTTCGCTGAAGCTCTTCTgcaGCAAATGCTTTGGTTGATTACTTCTTG
	hcp2_coLm-5gca-F	TTTTGTGTCGCCCTTCGCTGAAGCTCTTCTgcaGCTAACGCTTTAGTGGATTATTTCTTG
	hcp2_coLVS-5gca-F	ATTTTGTGTCGCCCTTCGCTGAAGCTCTTCTgcaGCGAATGCTTTGGTAGACTACTTTCT
**Construction of modified pM264-sacB MOTHER plasmids**		
**Purpose**	**Name**	**Sequence (5'→ 3')**
Construction of pM264(5'gca)-sacB by Gibson Assembly	sacB-(5gca)-F	TTTTGTGTCGCCCTTCGCTGAAGCTCTTCTgcaCTTCAGTTTAAACGAGTCGAGCCTGCA
	pM264-RP	TCACAAAGCAAATAAATTTTTCATGA
Construction of pM264(3'gca)-sacB by Gibson Assembly	pM264-FP	TGTTATCAGTATTTATTATGCATTTAGAAT
	sacB-(3gca)-R	CATGATTTGTGTCGCCCTTTTGACGGCTCTTCTtgcACTCACTATAGGGTCAGTGCGGCC
Construction of pM264(5'tct)-sacB by IVA	pM264(5tct)-mutF	CTTCGCTGAAGCTCTTCTtctCTTCAGTTTAAACGAGTCGAGCCTGC
	pM264(5xxx)-mutR	AGAAGAGCTTCAGCGAAGGGCGA
Construction of pM264(3'tct)-sacB and pM264(5'gca, 3'tct)-sacB by IVA	pM264(3tct)-mutF	ACTGACCCTATAGTGAGTtctAGAAGAGCCGTCAAAAGGG
	pM264(3xxx)-mutR	ACTCACTATAGGGTCAGTGCG

Supplementary Table S2 (continued). Oligonucleotides

Construction of modified pFNLdA DAUGHTER plasmids by IVA		
**Purpose**	**Name**	**Sequence (5'→ 3')**
Construction of pFNLdA-bfr-D11(5'gca)-sacB	pFNL-D1-(5gca)-mutF	gtaacaataggaggtacgtaATGgcaAGAAGAGCCTCACAGTTTAAACGAGTCG
	pFNL-D1-(5xxx)-mutR	TACGTACCTCCTATTGTTACCTCCATTATTTAA
Construction of pFNLdA-bfr-D12(5'tct)-sacB	pFNL-D1-(5tct)-mutF	gtaacaataggaggtacgtaATGtctAGAAGAGCCTCACAGTTTAAACGAGTCG
	pFNL-D1-(5xxx)-mutR	TACGTACCTCCTATTGTTACCTCCATTATTTAA
Construction of pFNLdA-bfr-D13(3'gca)-sacB	pFNL-D1,D2-(3gca)-mutF	ttaaaccgtaatgctcttcagcaTAAGGATCCACTAGCTCGTTTCAAATTACCGA
	pFNL-D1-(3xxx)-mutR	TGAAGAGCATTACGGTTTAAACAGAAGACATTG
Construction of pFNLdA-bfr-D14(3'tct)-sacB	pFNL-D1,D2-(3tct)-mutF	ttaaaccgtaatgctcttcatctTAAGGATCCACTAGCTCGTTTCAAATTACCGA
	pFNL-D1-(3xxx)-mutR	TGAAGAGCATTACGGTTTAAACAGAAGACATTG
Construction of pFNLdA-bfr-D21(5'gca)[N3F-8H]-sacB	pFNL-D2-(5gca)-mutF	catcatggaggtggttcaATGgcaAGAAGAGCCTCACAGTTTAAACAGA
	pFNL-D2-(5xxx)-mutR	TGAACCACCTCCATGATGATG
Construction of pFNLdA-bfr-D22(5'tct)[N3F-8H]-sacB	pFNL-D2-(5tct)-mutF	catcatggaggtggttcaATGtctAGAAGAGCCTCACAGTTTAAACAGA
	pFNL-D2-(5xxx)-mutR	TGAACCACCTCCATGATGATG
Construction of pFNLdA-bfr-D23(3'gca)[N3F-8H]-sacB	pFNL-D1,D2-(3gca)-mutF	ttaaaccgtaatgctcttcagcaTAAGGATCCACTAGCTCGTTTCAAATTACCGA
	pFNL-D2,D3-(3xxx)-mutR	TGAAGAGCATTACGGTTTAAACGAGTCGAG
Construction of pFNLdA-bfr-D24(3'tct)[N3F-8H]-sacB	pFNL-D1,D2-(3tct)-mutF	ttaaaccgtaatgctcttcatctTAAGGATCCACTAGCTCGTTTCAAATTACCGA
	pFNL-D2,D3-(3xxx)-mutR	TGAAGAGCATTACGGTTTAAACGAGTCGAG
Construction of pFNLdA-bfr-D31(5'gca)[C8H-3F]-sacB	pFNL-D3-(5gca)-mutF	gtaacaataggaggtacgtaATGgcaAGAAGAGCCTCACAGTTTAAACAGAAGACATT
	pFNL-D3-(5xxx)-mutR	TACGTACCTCCTATTGTTACCTCCATTATTTAA
Construction of pFNLdA-bfr-D32(5'tct)[C8H-3F]-sacB	pFNL-D3-(5tct)-mutF	gtaacaataggaggtacgtaATGtctAGAAGAGCCTCACAGTTTAAACAGAAGACATT
	pFNL-D3-(5xxx)-mutR	TACGTACCTCCTATTGTTACCTCCATTATTTAA
Construction of pFNLdA-bfr-D33(3'gca)[C8H-3F]-sacB	pFNL-D3-(3gca)-mutF	TTaaaccgtaatgctcttcagcaGGAGGTTCACATCATCATCACCACCATC
	pFNL-D2,D3-(3xxx)-mutR	TGAAGAGCATTACGGTTTAAACGAGTCGAG
Construction of pFNLdA-bfr-D34(3'tct)[C8H-3F]-sacB	pFNL-D3-(3tct)-mutF	TTaaaccgtaatgctcttcatctGGAGGTTCACATCATCATCACCACCATC
	pFNL-D2,D3-(3xxx)-mutR	TGAAGAGCATTACGGTTTAAACGAGTCGAG

Supplementary Table S2 (continued). Oligonucleotides

Cloning of two-antigen ORFs in pM264 MOTHER plasmids		
Construction of pM264(3'gca)-Hcp6 (coLm)-(GSSG)2-Hcp1 (coLVS)	hcp6_coLm-(ATG)-F	CCCTTCGCTGAAGCTCTTCTATGTTACACATGCACTTGAAATTCGGA
	hcp1_coLVS-(GCA)-R	GCCCTTTTGACGGCTCTTCTTGCATTAGTCCAATTTGCAGCACCTG
Construction of pM264(3'tct)-Hcp6 (coLm)-(GSSG)2-Hcp1 (coLVS)	hcp6_coLm-(ATG)-F	CCCTTCGCTGAAGCTCTTCTATGTTACACATGCACTTGAAATTCGGA
	hcp1_coLVS-(TCT)-R	GCCCTTTTGACGGCTCTTCTagaATTAGTCCAATTTGCAGCACCTG
Construction of pM264(5'tct)-LolC (coLVS)-(GSSG)2-Hcp2 (coLVS)	lolC_coLVS-(TCT)-F	CCCTTCGCTGAAGCTCTTCTtctAATGGATTTCAAAAAGAAGTTAGAGATAGAATGTT
	hcp2_coLVS-(GGT)-R	GCCCTTTTGACGGCTCTTCTaccAATTGGAGCATTTTGTTTCAGATCATATCCTG

For PCR primers, the portion of the primer that anneals to the template is underlined. For complementary oligonucleotides annealed together, the annealing portion of the oligonucleotide is underlined. Blue, bold font indicates homology used for cloning involving recombination (Gibson Assembly or IVA cloning). Green, bold font indicates the sequence coding for the GSSG-GSSG peptide linker. SapI overhangs are shown in red, bold font.

Supplementary Table S3. pFNL and pFNLdA Electra DAUGHTER Plasmids Constructed in this Study

Plasmid	Description	5' SapI cloning site	3' SapI cloning site	Antibiotic Resistance
pFNL-bfr-D1 (sacB)	Electra compatible *E. coli-Francisella* shuttle expression vector (DAUGHTER plasmid with a *sacB* insert for sucrose counterselection)	ATG (Met)	GGT (Gly)	Km^r Amp^r
pFNL-bfr-D2[N3F-8H] (sacB)	Electra compatible *E. coli-Francisella* shuttle expression vector (DAUGHTER plasmid with sacB insert for sucrose counterselection), N-terminal fusion of a dual 3xFLAG-His8 tag	ATG (Met)	GGT (Gly)	Km^r Amp^r
pFNL-bfr-D3[C8H-3F] (sacB)	Electra compatible *E. coli-Francisella* shuttle expression vector (DAUGHTER plasmid with sacB insert for sucrose counterselection), C-terminal fusion of a dual His8-3x FLAG tag	ATG (Met)	GGT (Gly)	Km^r Amp^r
pFNLdA-bfr-D1 (sacB)	Derivative of pFNL-bfr-D1 (sacB) with ampicillin resistance gene removed	ATG (Met)	GGT (Gly)	Km^r
pFNLdA-bfr-D2[N3F-8H] (sacB)	Derivative of pFNL-bfr-D2[N3F-8H] (sacB) with ampicillin resistance gene removed	ATG (Met)	GGT (Gly)	Km^r
pFNLdA-bfr-D3[C8H-3F] (sacB)	Derivative of pFNL-bfr-D3[C8H-3F] (sacB) with ampicillin resistance gene removed	ATG (Met)	GGT (Gly)	Km^r

Supplementary Table S3 (continued). pFNL and pFNLdA Electra DAUGHTER Plasmids Constructed in this Study

Plasmid	Description	5’ SapI cloning site	3’ SapI cloning site	Antibiotic Resistance
pFNLdA-bfr-D11(5'gca)-sacB	Derivatives of pFNLdA-bfr-D1 (sacB) with modified SapI cloning sites which allow for cloning ORFs from modified pM264 MOTHER plasmids	GCA (Ala)	GGT (Gly)	Km^r
pFNLdA-bfr-D12(5'tct)-sacB		TCT (Ser)	GGT (Gly)	Km^r
pFNLdA-bfr-D13(3'gca)-sacB		ATG (Met)	GCA (Ala)	Km^r
pFNLdA-bfr-D14(3'tct)-sacB		ATG (Met)	TCT (Ser)	Km^r
pFNLdA-bfr-D21(5'gca)[N3F-8H]-sacB	Derivative of pFNL-bfr-D2[N3F-8H] (sacB) with modified SapI cloning sites which allow for cloning ORFs from modified pM264 MOTHER plasmids	GCA (Ala)	GGT (Gly)	Km^r
pFNLdA-bfr-D22(5'tct)[N3F-8H]-sacB		TCT (Ser)	GGT (Gly)	Km^r
pFNLdA-bfr-D23(3'gca)[N3F-8H]-sacB		ATG (Met)	GCA (Ala)	Km^r
pFNLdA-bfr-D24(3'tct)[N3F-8H]-sacB		ATG (Met)	TCT (Ser)	Km^r
pFNLdA-bfr-D31(5'gca)[C8H-3F]-sacB	Derivative of pFNLdA-bfr-D3[C8H-3F] (sacB) with modified SapI cloning sites which allow for cloning ORFs from modified pM264 MOTHER plasmids	GCA (Ala)	GGT (Gly)	Km^r
pFNLdA-bfr-D32(5'tct)[C8H-3F]-sacB		TCT (Ser)	GGT (Gly)	Km^r
pFNLdA-bfr-D33(3'gca)[C8H-3F]-sacB		ATG (Met)	GCA (Ala)	Km^r
pFNLdA-bfr-D34(3'tct)[C8H-3F]-sacB		ATG (Met)	TCT (Ser)	Km^r

Supplementary Table S4. Plasmid Maps, SapI Cloning Site Maps, and DNA Sequences of Cloning Sites

Bacterioferritin promoter present in pFNL Electra DAUGHTER plasmids

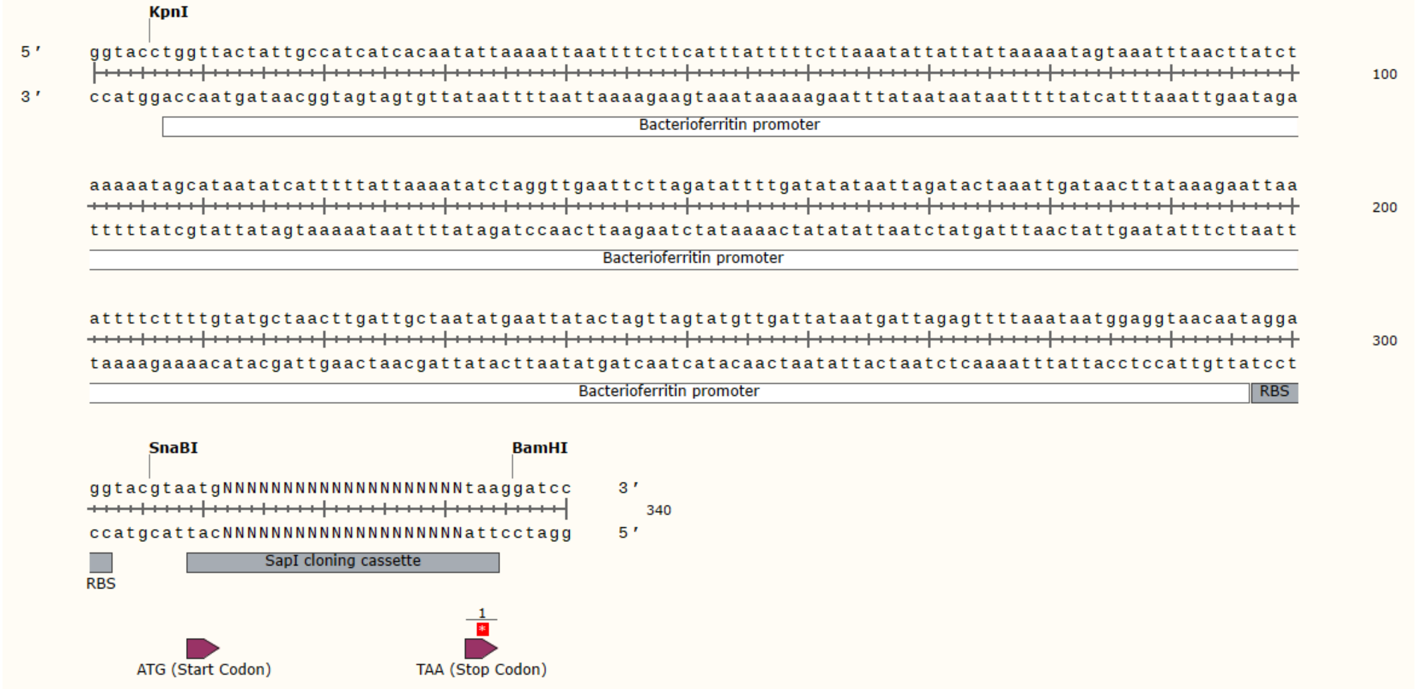

ggtacctggttactattgccatcatcacaatattaaaattaattttcttcatttatttttcttaaatattattattaaaaatagtaaatttaacttatctaaaaatagcataatatcatttttattaaaatatctaggttgaattcttagatattttgatatataattagatactaaattgataacttataaagaattaaattttcttttgtatgctaacttgattgctaatatgaattatactagttagtatgttgattataatgattagagttttaaataatggaggtaacaat**aggagg**tacgta**ATG**…[SapI cloning cassette]…**taa**ggatcc

Supplementary Table S4 (continued). SapI Plasmid Maps, SapI Cloning Site Maps, and DNA Sequences of Cloning Sites

pFNL-bfr-D1 (sacB)
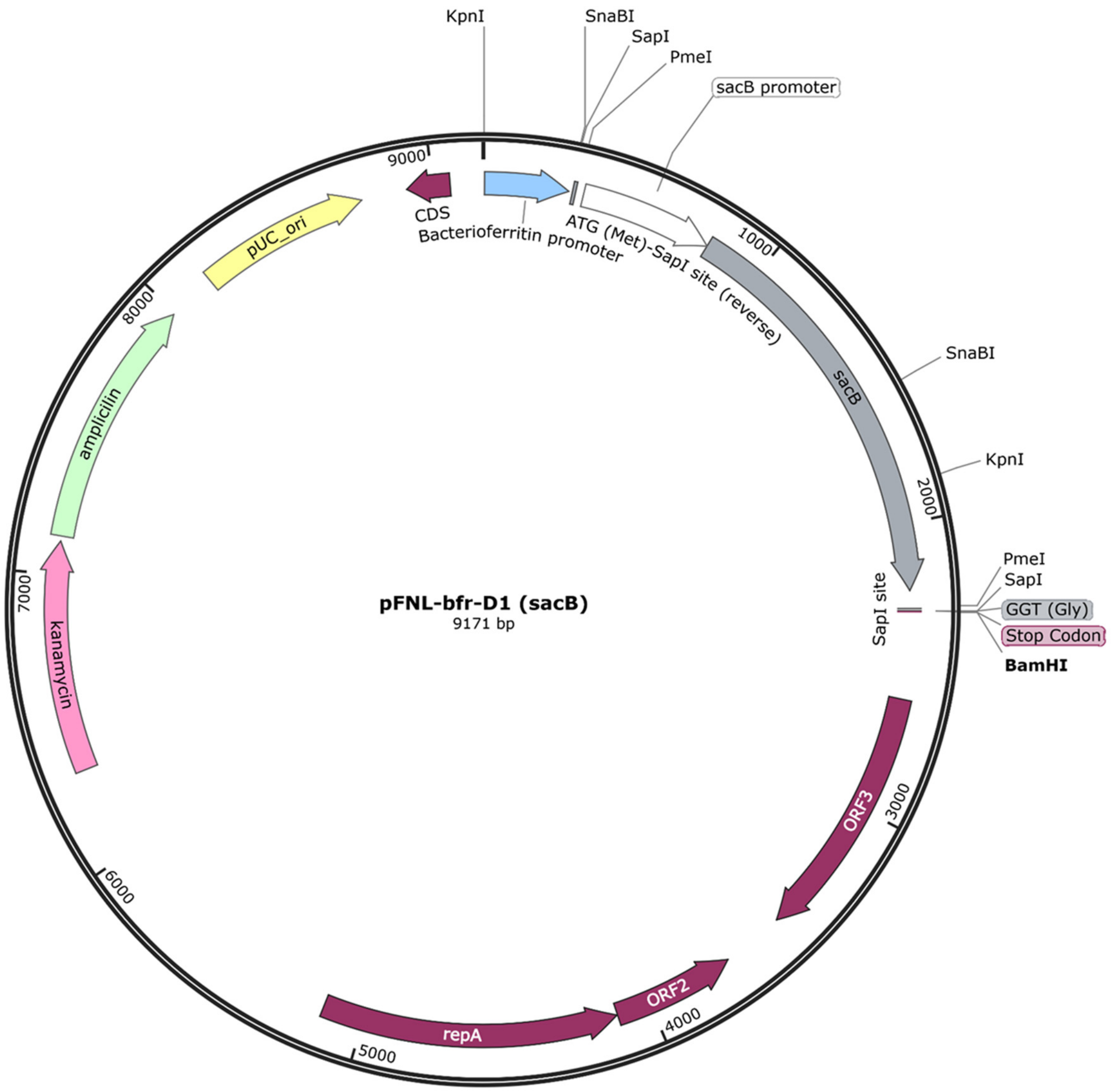
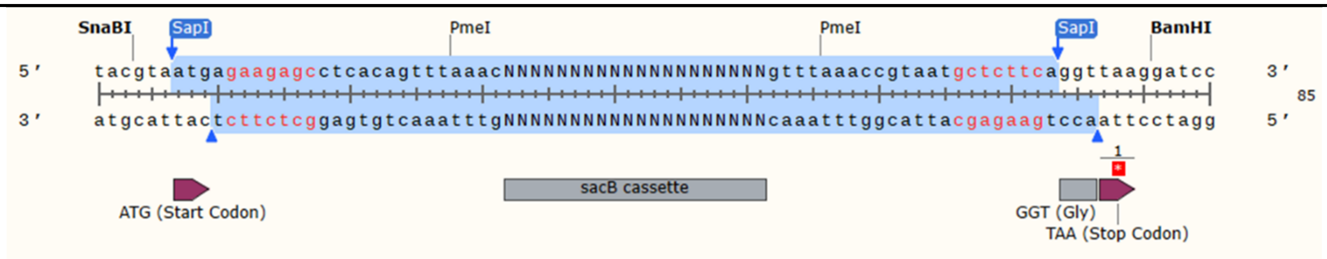
tacgta**ATG**agaagagcctcacagtttaaac…[*sacB*_cassette]…gtttaaaccgtaatgctcttca**GGT**__taa__ggatcc

Supplementary Table S4 (continued). Plasmid Maps, SapI Cloning Site Maps, and DNA Sequences of Cloning Sites

pFNL-bfr-D2[N3F-8H] (sacB)

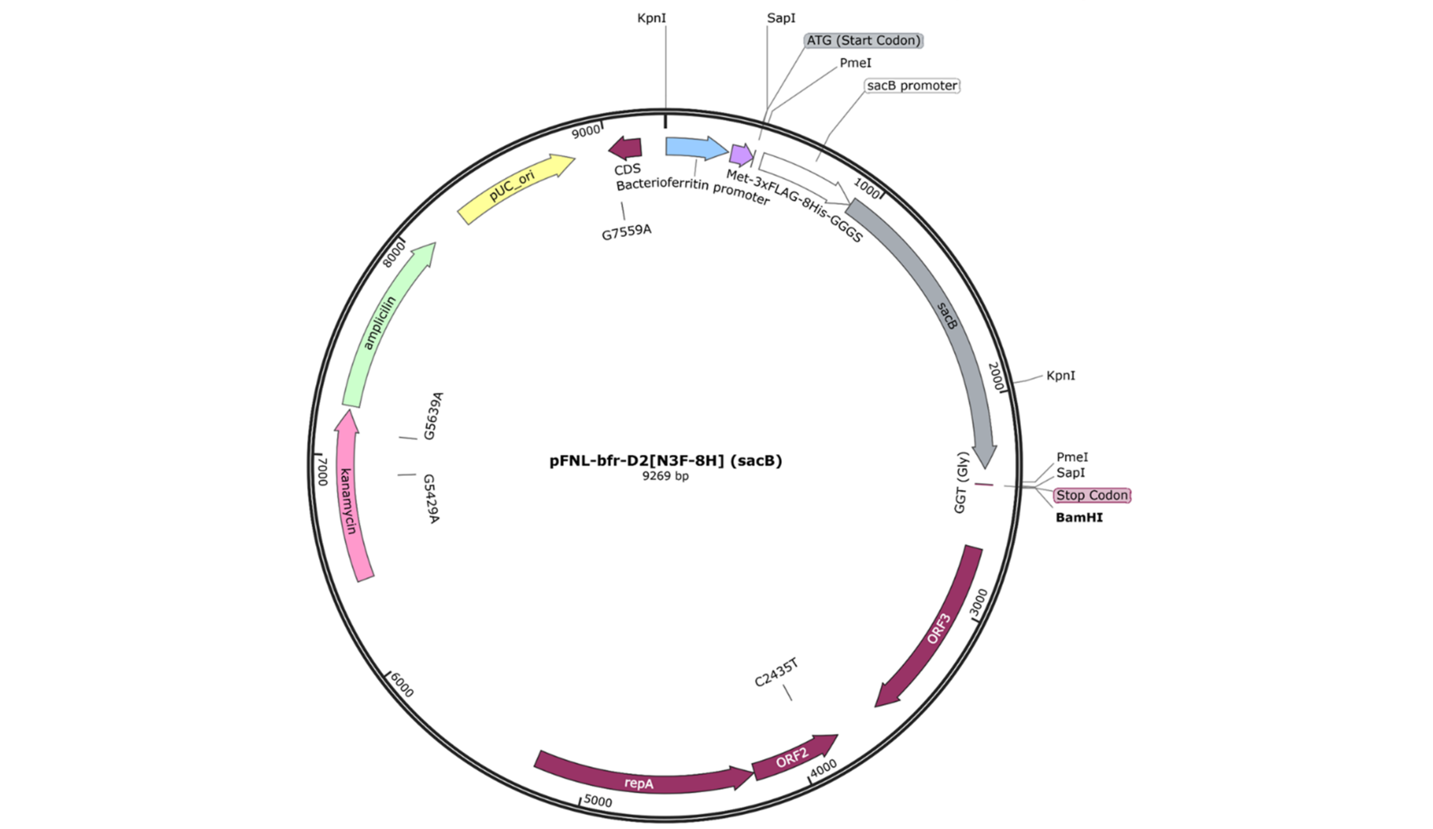

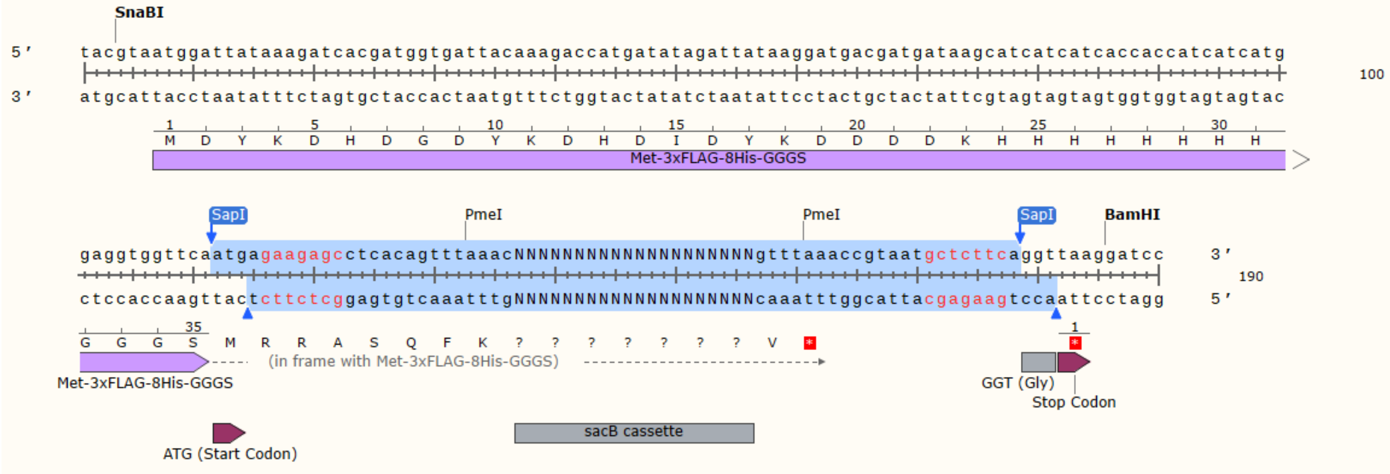

tacgta**atggattataaagatcacgatggtgattacaaagaccatgatatagattataaggatgacgatgataagcatcatcatcaccaccatcatcat ggaggtggttca****ATG**agaagagcctcacagtttaaac…[*sacB*_cassette]…gtttaaaccgtaatgctcttca**GGT****taa**ggatcc

Supplementary Table S4 (continued). Plasmid Maps, SapI Cloning Site Maps, and DNA Sequences of Cloning Sites

pFNL-bfr-D3[C8H-3F] (sacB)

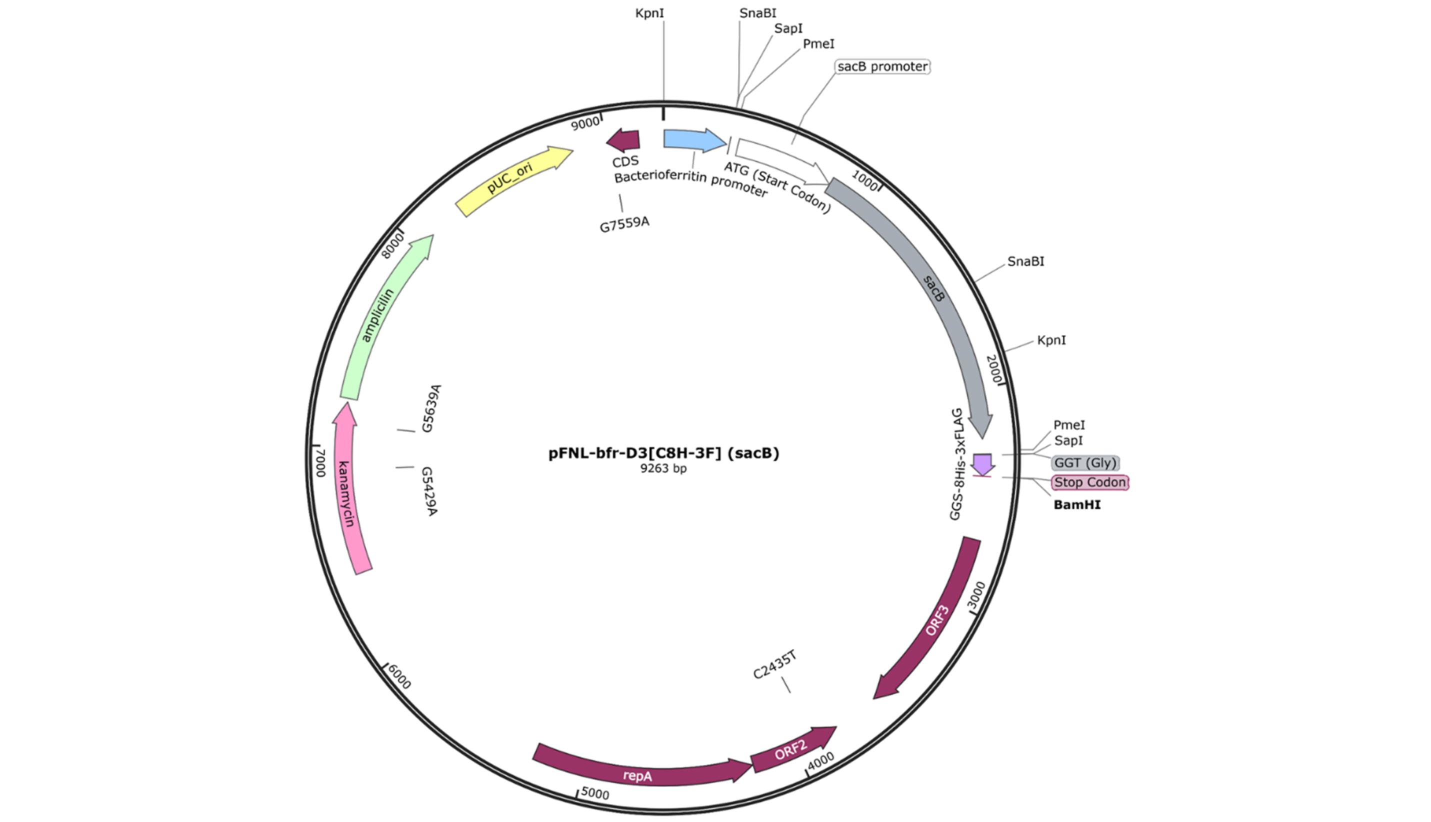

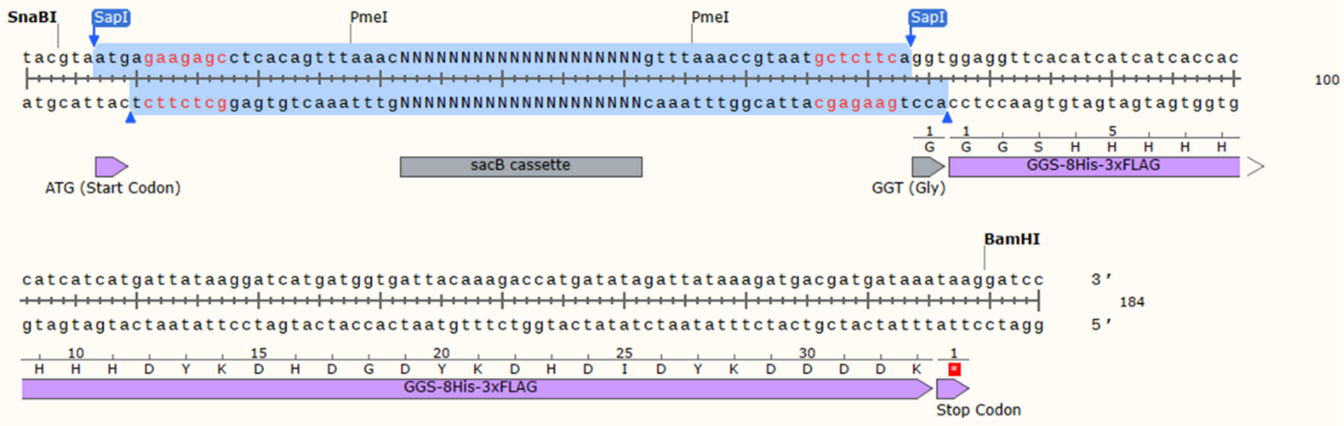

tacgta**ATG**agaagagcctcacagtttaaac…[*sacB*_cassette]…gtttaaaccgtaatgctcttca**ggtggaggttcacatcatcatcaccacca tcatcatgattataaggatcatgatggtgattacaaagaccatgatatagattataaagatgacgatgataaa****taa**ggatcc

Supplementary Table S4 (continued). Plasmid Maps, SapI Cloning Site Maps, and DNA Sequences of Cloning Sites

pM264-sacB

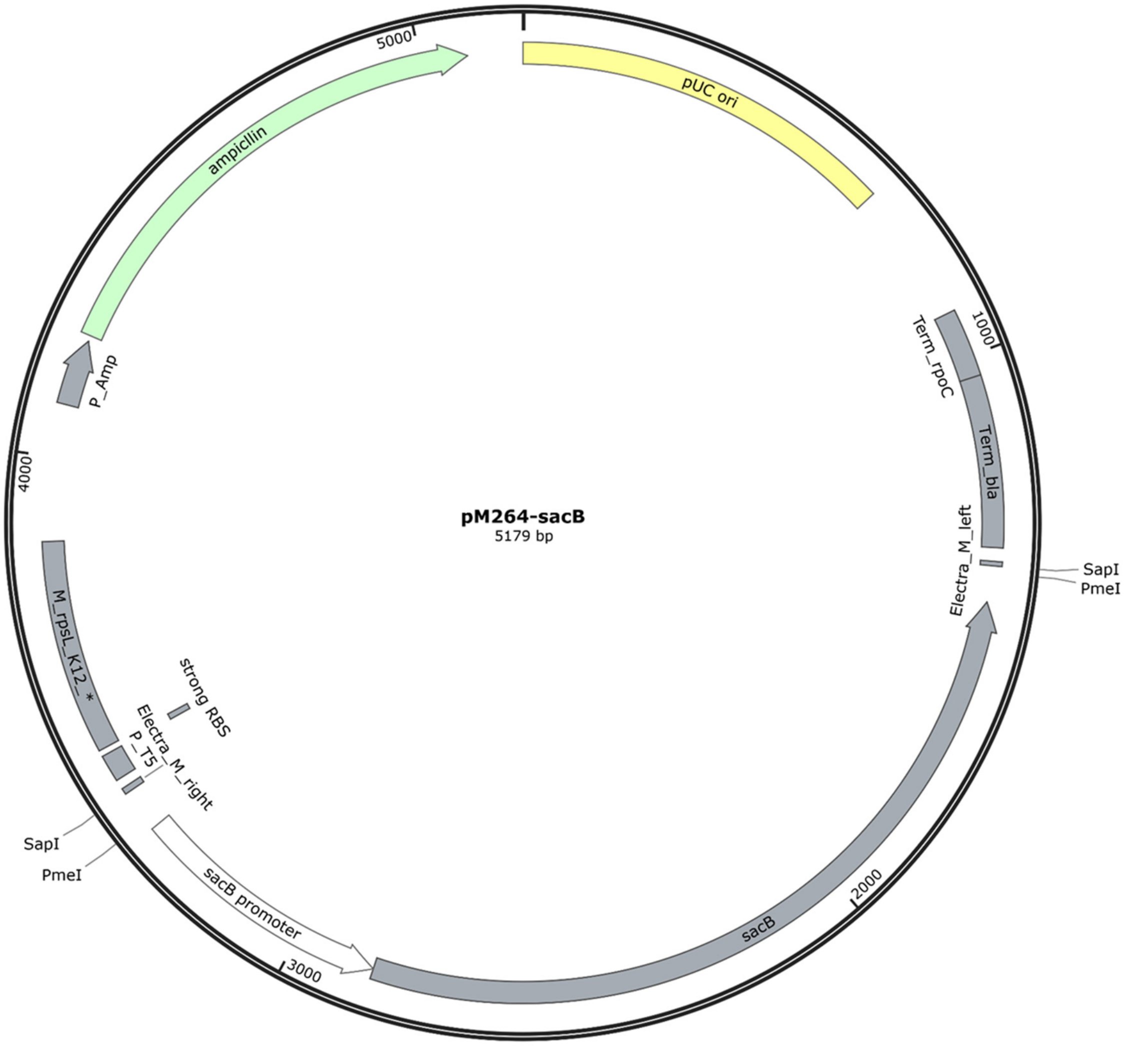

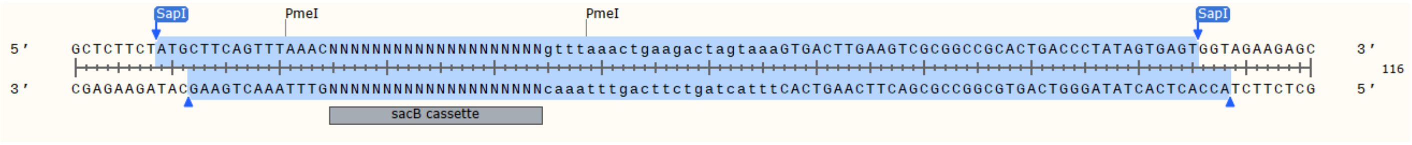

GCTCTTCT**ATG**CTTCAGTTTAAAC…[*sacB*_cassette]…gtttaaactgaagactagtaaaGTGACTTGAAGTCGC GGCCGCACTGACCCTATAGTGAGT**GGT**AGAAGAGC

Supplementary Table S4 (continued). Plasmid Maps, SapI Cloning Site Maps, and DNA Sequences of Cloning Sites

**pM264(5'gca)-sacB: SapI cloning sites**
GCTCTTCT**GCA**CTTCAGTTTAAAC…[*sacB*_cassette]…gtttaaactgaagactagtaaaGTGACTTGAAGTCGC<br>GGCCGCACTGACCCTATAGTGAGT**GGT**AGAAGAGC
**pM264(3'gca)-sacB: SapI cloning sites**
GCTCTTCT**ATG**CTTCAGTTTAAAC…[*sacB*_cassette]…gtttaaactgaagactagtaaaGTGACTTGAAGTCGC<br>GGCCGCACTGACCCTATAGTGAGT**GCA**AGAAGAGC
**pM264(5'tct)-sacB: SapI cloning sites**
GCTCTTCT**TCT**CTTCAGTTTAAAC…[*sacB*_cassette]…gtttaaactgaagactagtaaaGTGACTTGAAGTCGC<br>GGCCGCACTGACCCTATAGTGAGT**GGT**AGAAGAGC
**pM264(3'tct)-sacB: SapI cloning sites**
GCTCTTCT**ATG**CTTCAGTTTAAAC…[*sacB*_cassette]…gtttaaactgaagactagtaaaGTGACTTGAAGTCGC<br>GGCCGCACTGACCCTATAGTGAGT**TCT**AGAAGAGC
**pM264(5'gca, 3'tct)-sacB: SapI cloning sites**
GCTCTTCT**GCA**CTTCAGTTTAAAC…[*sacB*_cassette]…gtttaaactgaagactagtaaaGTGACTTGAAGTCGC<br>GGCCGCACTGACCCTATAGTGAGT**TCT**AGAAGAGC

Supplementary Table S4 (continued). Plasmid Maps, SapI Cloning Site Maps, and DNA Sequences of Cloning Sites

GSAGSAAGSGEF flexible linker in pM264(5'gca,3'tct)-GSAGSAAGSGEF

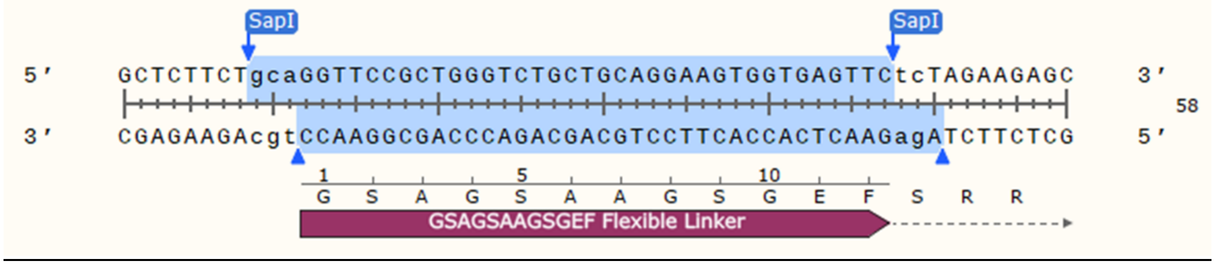

GCTCTTCT**gca**GGTTCCGCTGGGTCTGCTGCAGGAAGTGGTGAGTTC**tcT**AGAAGAGC

A(EAAAK)3A rigid linker in pM264(5'gca,3'tct)-A(EAAAK)3A

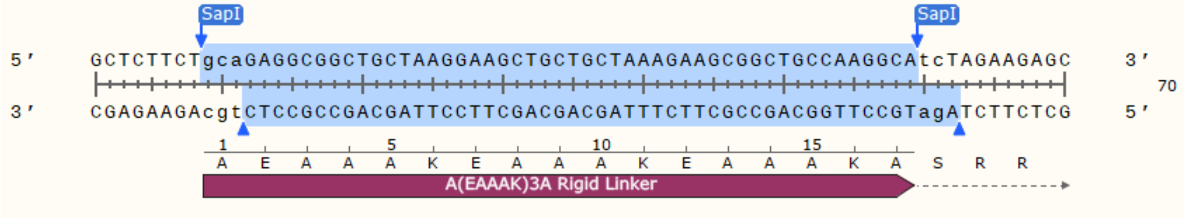

GCTCTTCT**gca**GAGGCGGCTGCTAAGGAAGCTGCTGCTAAAGAAGCGGCTGCCAAGGCA**tcT**AGA
AGAGC

(AP)10 rigid linker in pM264(5'gca,3'tct)-(AP)10

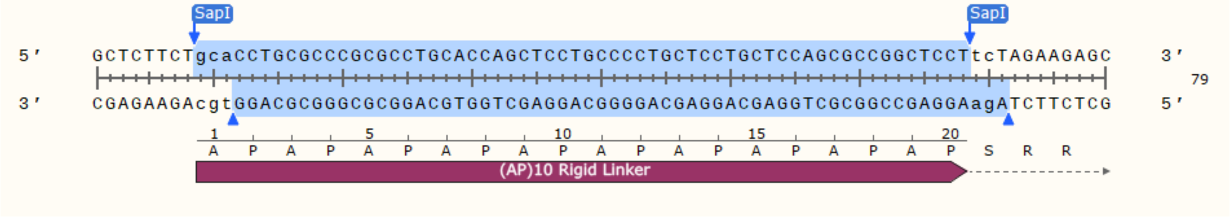

GCTCTTCT**gca**CCTGCGCCCGCGCCTGCACCAGCTCCTGCCCCTGCTCCTGCTCCAGCGCCGGCTC
CT**tcT**AGAAGAGC

Green font = bacterioferritin promoter
Red font = ribosomal binding site
Blue font = SapI overhangs (ATG, start codon; GGT, Gly)
Dark Red font = SapI overhangs (GCA, Ala; TCT, Ser)
Purple font = stop codon (taa)
Orange font = fusion tag
Underline = restriction site

Supplementary Table S5. pM264 derived Electra MOTHER Cloning Plasmids Constructed in this Study

Plasmid	Description	5' SapI cloning site	3' SapI cloning site	Antibiotic Resistance
pM264-sacB	Derivative of pM264 MOTHER plasmid with a *sacB* insert for sucrose counterselection	ATG (Met)	GGT (Gly)	Amp^r
pM264(5'gca)-sacB	Derivative of pM264-sacB MOTHER plasmid with modified SapI cloning site	GCA (Ala)	GGT (Gly)	Amp^r
pM264(3'gca)-sacB	Derivative of pM264-sacB MOTHER plasmid with modified SapI cloning site	ATG (Met)	GCA (Ala)	Amp^r
pM264(5'tct)-sacB	Derivative of pM264-sacB MOTHER plasmid with modified SapI cloning site	TCT (Ser)	GGT (Gly)	Amp^r
pM264(3'tct)-sacB	Derivative of pM264-sacB MOTHER plasmid with modified SapI cloning site	ATG (Met)	TCT (Ser)	Amp^r
pM264(5'gca, 3'tct)-sacB	Derivative of pM264-sacB MOTHER plasmid with modified SapI cloning sites	GCA (Ala)	TCT (Ser)	Amp^r

Supplementary Table S6. MOTHER Plasmids with Bp antigens and Peptide Linkers Constructed in this Study

Plasmid	Description	5' SapI cloning site	3' SapI cloning site	Antibiotic Resistance
pM264-Bp hcp6 (coLm)	*B. pseudomallei hcp6* gene codon optimized for *L. monocytogenes*	ATG (Met)	GGT (Gly)	Amp^r
pM268-Bp hcp6 (coLVS)	*B. pseudomallei hcp6* gene codon optimized for *F. tularensis* LVS	ATG (Met)	GGT (Gly)	Amp^r
pM264-Bp hcp6 (native)	*B. pseudomallei hcp6* gene (native)	ATG (Met)	GGT (Gly)	Amp^r
pM264-Bp hcp1 (coLm)	*B. pseudomallei hcp1* gene codon optimized for *L. monocytogenes*	ATG (Met)	GGT (Gly)	Amp^r
pM264-Bp hcp1 (coLVS)	*B. pseudomallei hcp1* gene codon optimized for *F. tularensis* LVS	ATG (Met)	GGT (Gly)	Amp^r
pM264-Bp hcp1 (native)	*B. pseudomallei hcp1* gene (native)	ATG (Met)	GGT (Gly)	Amp^r
pM264-Bp hcp2 (coLm)	*B. pseudomallei hcp2* gene codon optimized for *L. monocytogenes*	ATG (Met)	GGT (Gly)	Amp^r
pM264-Bp hcp2 (coLVS)	*B. pseudomallei hcp2* gene codon optimized for *F. tularensis* LVS	ATG (Met)	GGT (Gly)	Amp^r
pM264-Bp hcp2 (native)	*B. pseudomallei hcp2* gene (native)	ATG (Met)	GGT (Gly)	Amp^r
pM264-Bp lolC 51-273 (coLm)	*B. pseudomallei lolC* gene codon optimized for *L. monocytogenes* (periplasmic region, amino acid residues 51 to 273)	ATG (Met)	GGT (Gly)	Amp^r
pM264-Bp lolC 51-273 (coLVS)	*B. pseudomallei lolC* gene codon optimized for *F. tularensis* LVS (periplasmic region, amino acid residues 51 to 273)	ATG (Met)	GGT (Gly)	Amp^r

Supplementary Table S6 (continued). MOTHER Plasmids with Bp antigens and Peptide Linkers Constructed in this Study

Plasmid	Description	5' SapI cloning site	3' SapI cloning site	Antibiotic Resistance
pM264(5'gca)-Bp hcp1 (coLm)	Derivative of pM264-Bp hcp1 (coLm) with a 5'GCA SapI overhang	GCA (Ala)	GGT (Gly)	Amp^r
pM264(5'gca)-Bp hcp1 (coLVS)	Derivative of pM264-Bp hcp1 (coLVS) with a 5'GCA SapI overhang	GCA (Ala)	GGT (Gly)	Amp^r
pM264(5'gca)-Bp hcp1 (native)	Derivative of pM264-Bp hcp1 (native) with a 5'GCA SapI overhang	GCA (Ala)	GGT (Gly)	Amp^r
pM264(5'gca)-Bp hcp2 (coLm)	Derivative of pM264-Bp hcp2 (coLm) with a 5'GCA SapI overhang	GCA (Ala)	GGT (Gly)	Amp^r
pM264(5'gca)-Bp hcp2 (coLVS)	Derivative of pM264-Bp hcp2 (coLVS) with a 5'GCA SapI overhang	GCA (Ala)	GGT (Gly)	Amp^r
pM264(5'gca)-Bp hcp2 (native)	Derivative of pM264-Bp hcp2 (native) with a 5'GCA SapI overhang	GCA (Ala)	GGT (Gly)	Amp^r
pM264(3'gca)-Bp hcp6 (coLm)-(GSSG)2	*B. pseudomallei hcp6* gene codon optimized for *L. monocytogenes* with a C-terminal GSSG-GSSG flexible peptide linker	ATG (Met)	GCA (Ala)	Amp^r
pM264(3'gca)-Bp lolC 51-273 (coLm)-(GSSG)2	*B. pseudomallei lolC* gene codon optimized for *L. monocytogenes* (periplasmic region, amino acid residues 51 to 273) with a C-terminal GSSG-GSSG flexible peptide linker	ATG (Met)	GCA (Ala)	Amp^r
pM264(3'gca)-Bp lolC 51-273 (coLVS)-(GSSG)2	*B. pseudomallei lolC* gene codon optimized for *F. tularensis* LVS (periplasmic region, amino acid residues 51 to 273) with a C-terminal GSSG-GSSG flexible peptide linker<br>*The second codon of lolC in this construct is CAT (His) instead of AAT(Asn) due to a cloning error.*	ATG (Met)	GCA (Ala)	Amp^r

Supplementary Table S6 (continued). MOTHER Plasmids with Bp antigens and Peptide Linkers Constructed in this Study

Plasmid	Description	5' SapI cloning site	3' SapI cloning site	Antibiotic Resistance
pM264(5'gca,3'tct)-GSAGSAAGSGEF	GSAGSAAGSGEF flexible peptide linker	GCA (Ala)	TCT (Ser)	Amp^r
pM264(5'gca,3'tct)-(AP)10	$(AP)_{10}$ rigid peptide linker	GCA (Ala)	TCT (Ser)	Amp^r
pM264(5'gca,3'tct)-A(EAAAK)3A	$A(EAAAK)_3A$ rigid peptide linker	GCA (Ala)	TCT (Ser)	Amp^r
pM264(3'gca)-LolC (coLVS)	LolC suitable for linking to a downstream compatible ORF	ATG (Met)	GCA (Ala)	Amp^r
pM264(5'tct)-LolC (coLVS)	LolC suitable for linking to an upstream compatible ORF	TCT (Ser)	GGT (Gly)	Amp^r
pM264(5'gca)-LolC (coLVS)	LolC suitable for linking to an upstream compatible ORF	GCA (Ala)	GGT (Gly)	Amp^r
pM264(5'tct)-LolC (coLVS)-(GSSG)2-Hcp2 (coLVS)	LolC-(GSSG)2-Hcp2 fusion suitable for linking to an upstream compatible ORF	TCT (Ser)	GGT (Gly)	Amp^r
pM264(3'gca)-Hcp6 (coLm)-(GSSG)2-Hcp1 (coLVS)	Hcp6-(GSSG)2-Hcp1 fusion suitable for linking to a downstream compatible ORF	ATG (Met)	GCA (Ala)	Amp^r
pM264(3'tct)-Hcp6 (coLm)-(GSSG)2-Hcp1 (coLVS)	Hcp6-(GSSG)2-Hcp1 fusion suitable for linking to a downstream compatible ORF	ATG (Met)	TCT (Ser)	Amp^r
pM264(5’tct)-Bp Hcp6 (coLm)-(GSSG)2-Hcp1 (coLVS)	Hcp6-(GSSG)2-Hcp1 fusion suitable for linking to an upstream compatible ORF	TCT (Ser)	GGT (Gly)	Amp^r
pM264(5’gca)-Bp Hcp6 (coLm)-(GSSG)2-Hcp1 (coLVS)	Hcp6-(GSSG)2-Hcp1 fusion suitable for linking to an upstream compatible ORF	GCA (Ala)	GGT (Gly)	Amp^r

Supplementary Table S6 (continued). MOTHER Plasmids with Bp antigens and Peptide Linkers Constructed in this Study

Plasmid	Description	5' SapI cloning site	3' SapI cloning site	Antibiotic Resistance
pM264(3'gca)-Bp Hcp6 (coLm)-(GSSG)2-Hcp2 (coLVS)	Hcp6-(GSSG)2-Hcp2 fusion suitable for linking to a downstream compatible ORF	ATG (Met)	GCA (Ala)	Amp^r
pM264(5'tct)-Bp Hcp6 (coLm)-(GSSG)2-Hcp2 (coLVS)	Hcp6-(GSSG)2-Hcp2 fusion suitable for linking to an upstream compatible ORF	TCT (Ser)	GGT (Gly)	Amp^r
pM264(5'gca)-Bp Hcp6 (coLm)-(GSSG)2-Hcp2 (coLVS)	Hcp6-(GSSG)2-Hcp2 fusion suitable for linking to an upstream compatible ORF	GCA (Ala)	GGT (Gly)	Amp^r

Supplementary Table S7. pFNL and pFNLdA Expression Plasmids Constructed in this Study

Plasmid	Description	Version of Gene(s)	Fusion Tag	Antibiotic Resistance	Tested as a Vaccine
pFNL-bfr-Bp hcp6 (coLm)	Expression of Hcp6 from Pbfr promoter	coLm	None	Km^r Amp^r	
pFNL-bfr-Bp hcp6 (coLVS)		coLVS	None	Km^r Amp^r	
pFNL-bfr-Bp hcp6 (native)		native	None	Km^r Amp^r	
pFNL-bfr-N3F-8H-Bp hcp6 (coLm)		coLm	N-terminal 3xFLAG-His8	Km^r Amp^r	
pFNL-bfr-N3F-8H-Bp hcp6 (coLVS)		coLVS	N-terminal 3xFLAG-His8	Km^r Amp^r	
pFNL-bfr-N3F-8H-Bp hcp6 (native)		native	N-terminal 3xFLAG-His8	Km^r Amp^r	
pFNL-bfr-Bp hcp6 (coLm)-C8H-3F		coLm	C-terminal His8-3x FLAG	Km^r Amp^r	
pFNL-bfr-Bp hcp6 (coLVS)-C8H-3F		coLVS	C-terminal His8-3x FLAG	Km^r Amp^r	
pFNL-bfr-Bp hcp6 (native)-C8H-3F		native	C-terminal His8-3x FLAG	Km^r Amp^r	

Supplementary Table S7 (continued). pFNL and pFNLdA Expression Plasmids Constructed in this Study

Plasmid	Description	Version of Gene(s)	Fusion Tag	Antibiotic Resistance	Tested as a Vaccine
pFNL-bfr-Bp hcp1 (coLm)	Expression of Hcp1 from Pbfr promoter	coLm	None	Km^r Amp^r	
pFNL-bfr-Bp hcp1 (coLVS)		coLVS	None	Km^r Amp^r	
pFNL-bfr-Bp hcp1 (native)		native	None	Km^r Amp^r	
pFNL-bfr-N3F-8H-Bp hcp1 (coLm)		coLm	N-terminal 3xFLAG-His8	Km^r Amp^r	
pFNL-bfr-N3F-8H-Bp hcp1 (coLVS)		coLVS	N-terminal 3xFLAG-His8	Km^r Amp^r	
pFNL-bfr-N3F-8H-Bp hcp1 (native)		native	N-terminal 3xFLAG-His8	Km^r Amp^r	
pFNL-bfr-Bp hcp1 (coLm)-C8H-3F		coLm	C-terminal His8-3x FLAG	Km^r Amp^r	
pFNL-bfr-Bp hcp1 (coLVS)-C8H-3F		coLVS	C-terminal His8-3x FLAG	Km^r Amp^r	
pFNL-bfr-Bp hcp1 (native)-C8H-3F		native	C-terminal His8-3x FLAG	Km^r Amp^r	

Supplementary Table S7 (continued). pFNL and pFNLdA Expression Plasmids Constructed in this Study

Plasmid	Description	Version of Gene(s)	Fusion Tag	Antibiotic Resistance	Tested as a Vaccine
pFNL-bfr-Bp hcp2 (coLm)	Expression of Hcp2 from Pbfr promoter	coLm	None	Km^r Amp^r	
pFNL-bfr-Bp hcp2 (coLVS)		coLVS	None	Km^r Amp^r	
pFNL-bfr-Bp hcp2 (native)		native	None	Km^r Amp^r	
pFNL-bfr-N3F-8H-Bp hcp2 (coLm)		coLm	N-terminal 3xFLAG-His8	Km^r Amp^r	
pFNL-bfr-N3F-8H-Bp hcp2 (coLVS)		coLVS	N-terminal 3xFLAG-His8	Km^r Amp^r	
pFNL-bfr-N3F-8H-Bp hcp2 (native)		native	N-terminal 3xFLAG-His8	Km^r Amp^r	
pFNL-bfr-Bp hcp2 (coLm)-C8H-3F		coLm	C-terminal His8-3x FLAG	Km^r Amp^r	
pFNL-bfr-Bp hcp2 (coLVS)-C8H-3F		coLVS	C-terminal His8-3x FLAG	Km^r Amp^r	
pFNL-bfr-Bp hcp2 (native)-C8H-3F		native	C-terminal His8-3x FLAG	Km^r Amp^r	
pFNL-bfr-Bp lolC 51-273 (coLm)	Expression of periplasmic region of LolC (amino acid residues 51 to 273) from Pbfr promoter	coLm	None	Km^r Amp^r	
pFNL-bfr-Bp lolC 51-273 (coLVS)		coLVS	None	Km^r Amp^r	
pFNL-bfr-N3F-8H-Bp lolC 51-273 (coLVS)		coLVS	N-terminal 3xFLAG-His8	Km^r Amp^r	
pFNL-bfr-Bp lolC 51-273 (coLm)-C8H-3F		coLm	C-terminal His8-3x FLAG	Km^r Amp^r	
pFNL-bfr-Bp lolC 51-273 (coLVS)-C8H-3F		coLVS	C-terminal His8-3x FLAG	Km^r Amp^r	

Supplementary Table S7 (continued). pFNL and pFNLdA Expression Plasmids Constructed in this Study

Plasmid	Description	Version of Gene(s)	Fusion Tag	Antibiotic Resistance	Tested as a Vaccine
pFNL-bfr-Hcp6 (coLm)-(GSSG)2-Hcp1 (coLm)-C8H-3F	Expression of Hcp6-(GCCG)2-Hcp1 fusion protein from Pbfr promoter	coLm/coLm	C-terminal His8-3x FLAG	Km^r Amp^r	
pFNL-bfr-Hcp6 (coLm)-(GSSG)2-Hcp1 (coLVS)-C8H-3F		coLm/coLVS	C-terminal His8-3x FLAG	Km^r Amp^r	Yes
pFNL-bfr-Hcp6 (coLm)-(GSSG)2-Hcp1 (native)-C8H-3F		coLm/native	C-terminal His8-3x FLAG	Km^r Amp^r	
pFNL-bfr-Hcp6 (coLm)-(GSSG)2-Hcp2 (coLm)-C8H-3F	Expression of Hcp6-(GCCG)2-Hcp2 fusion protein from Pbfr promoter	coLm/coLm	C-terminal His8-3x FLAG	Km^r Amp^r	
pFNL-bfr-Hcp6 (coLm)-(GSSG)2-Hcp2 (coLVS)-C8H-3F		coLm/coLVS	C-terminal His8-3x FLAG	Km^r Amp^r	Yes
pFNL-bfr-Hcp6 (coLm)-(GSSG)2-Hcp2 (native)-C8H-3F		coLm/native	C-terminal His8-3x FLAG	Km^r Amp^r	
pFNL-bfr-LolC (coLVS)-(GSSG)2-Hcp1 (coLm)-C8H-3F	Expression of LolC-(GCCG)2-Hcp1 fusion protein from Pbfr promoter	coLVS/coLm	C-terminal His8-3x FLAG	Km^r Amp^r	
pFNL-bfr-LolC (coLVS)-(GSSG)2-Hcp1 (coLVS)-C8H-3F		coLVS/coLVS	C-terminal His8-3x FLAG	Km^r Amp^r	Yes
pFNL-bfr-LolC (coLVS)-(GSSG)2-Hcp1 (native)-C8H-3F		coLVS/native	C-terminal His8-3x FLAG	Km^r Amp^r	
pFNL-bfr-LolC (coLVS)-(GSSG)2-Hcp2 (coLm)-C8H-3F	Expression of LolC-(GCCG)2-Hcp2 fusion protein from Pbfr promoter	coLVS/coLm	C-terminal His8-3x FLAG	Km^r Amp^r	
pFNL-bfr-LolC (coLVS)-(GSSG)2-Hcp2 (coLVS)-C8H-3F		coLVS/coLVS	C-terminal His8-3x FLAG	Km^r Amp^r	Yes
pFNL-bfr-LolC (coLVS)-(GSSG)2-Hcp2 (native)-C8H-3F		coLVS/native	C-terminal His8-3x FLAG	Km^r Amp^r	

Supplementary Table S8. Electra Reactions linking LolC to Hcp6-(GSSG)2-Hcp1 or Hcp6-(GSSG)2-Hcp2

MOTHER plasmids (SapI sites used for joining ORFs shown below)							
**3'TCT**	**3'GCA**	**5'GCA, 3'TCT**	**5'TCT**	**5'GCA**	**Recombinant Plasmid**	**Fusion Protein**	**Used in a Vaccine**
	pM264(3'gca)-LolC (coLVS)	pM264(5'gca,3'tct)-GSAGSAAGSGEF	pM264(5'tct)-Bp Hcp6 (coLm)-(GSSG)2-Hcp1 (coLVS)		pFNLdA-bfr-LolC-GSAGSAAGSGEF-Hcp6-(GSSG)2-Hcp1-C8H-3F	LolC-GSAGSAAGSGEF-Hcp6-(GSSG)2-Hcp1-C8H-3F	
	pM264(3'gca)-LolC (coLVS)	pM264(5'gca,3'tct)-A(EAAAK)3A	pM264(5'tct)-Bp Hcp6 (coLm)-(GSSG)2-Hcp1 (coLVS)		pFNLdA-bfr-LolC-A(EAAAK)3A-Hcp6-(GSSG)2-Hcp1-C8H-3F	LolC-A(EAAAK)3A-Hcp6-(GSSG)2-Hcp1-C8H-3F	
	pM264(3'gca)-LolC (coLVS)	pM264(5'gca,3'tct)-(AP)10	pM264(5'tct)-Bp Hcp6 (coLm)-(GSSG)2-Hcp1 (coLVS)		pFNLdA-bfr-LolC-(AP)10-Hcp6-(GSSG)2-Hcp1-C8H-3F	LolC-(AP)10-Hcp6-(GSSG)2-Hcp1-C8H-3F	
	pM264(3'gca)-LolC (coLVS)			pM264(5'gca)-Bp Hcp6 (coLm)-(GSSG)2-Hcp1 (coLVS)	pFNLdA-bfr-LolC-Hcp6-(GSSG)2-Hcp1-C8H-3F	LolC-Hcp6-(GSSG)2-Hcp1-C8H-3F	

Supplementary Table S8 (continued). Electra Reactions linking LolC to Hcp6-(GSSG)2-Hcp1 or Hcp6-(GSSG)2-Hcp2

MOTHER plasmids (SapI sites used for joining ORFs shown below)							
**3'TCT**	**3'GCA**	**5'GCA, 3'TCT**	**5'TCT**	**5'GCA**	**Recombinant Plasmid**	**Fusion Protein**	**Used in a Vaccine**
	pM264(3'gca)-LolC (coLVS)	pM264(5'gca,3'tct)-GSAGSAAGSGEF	pM264(5'tct)-Bp Hcp6 (coLm)-(GSSG)2-Hcp2 (coLVS)		pFNLdA-bfr-LolC-GSAGSAAGSGEF-Hcp6-(GSSG)2-Hcp2-C8H-3F	LolC-GSAGSAAGSGEF-Hcp6-(GSSG)2-Hcp2-C8H-3F	
	pM264(3'gca)-LolC (coLVS)	pM264(5'gca,3'tct)-A(EAAAK)3A	pM264(5'tct)-Bp Hcp6 (coLm)-(GSSG)2-Hcp2 (coLVS)		pFNLdA-bfr-LolC-A(EAAAK)3A-Hcp6-(GSSG)2-Hcp2-C8H-3F	LolC-A(EAAAK)3A-Hcp6-(GSSG)2-Hcp2-C8H-3F	
	pM264(3'gca)-LolC (coLVS)	pM264(5'gca,3'tct)-(AP)10	pM264(5'tct)-Bp Hcp6 (coLm)-(GSSG)2-Hcp2 (coLVS)		pFNLdA-bfr-LolC-(AP)10-Hcp6-(GSSG)2-Hcp2-C8H-3F	LolC-(AP)10-Hcp6-(GSSG)2-Hcp2-C8H-3F	
	pM264(3'gca)-LolC (coLVS)			pM264(5'gca)-Bp Hcp6 (coLm)-(GSSG)2-Hcp2 (coLVS)	pFNLdA-bfr-LolC-Hcp6-(GSSG)2-Hcp2-C8H-3F	LolC-Hcp6-(GSSG)2-Hcp2-C8H-3F	

Supplementary Table S8 (continued). Electra Reactions linking LolC to Hcp6-(GSSG)2-Hcp1 or Hcp6-(GSSG)2-Hcp2

MOTHER plasmids (SapI sites used for joining ORFs shown below)							
**3'TCT**	**3'GCA**	**5'GCA, 3'TCT**	**5'TCT**	**5'GCA**	**Recombinant Plasmid**	**Fusion Protein**	**Used in a Vaccine**
	pM264(3'gca)-Hcp6 (coLm)-(GSSG)2-Hcp1 (coLVS)	pM264(5'gca,3'tct)-GSAGSAAGSGEF	pM264(5'tct)-LolC (coLVS)		pFNLdA-bfr-Hcp6-(GSSG)2-Hcp1-GSAGSAAGSGEF-LolC-C8H-3F	Hcp6-(GSSG)2-Hcp1-GSAGSAAGSGEF-LolC-C8H-3F	Yes
	pM264(3'gca)-Hcp6 (coLm)-(GSSG)2-Hcp1 (coLVS)	pM264(5'gca,3'tct)-A(EAAAK)3A	pM264(5'tct)-LolC (coLVS)		pFNLdA-bfr-Hcp6-(GSSG)2-Hcp1-A(EAAAK)3A-LolC-C8H-3F	Hcp6-(GSSG)2-Hcp1-A(EAAAK)3A-LolC-C8H-3F	
	pM264(3'gca)-Hcp6 (coLm)-(GSSG)2-Hcp1 (coLVS)	pM264(5'gca,3'tct)-(AP)10	pM264(5'tct)-LolC (coLVS)		pFNLdA-bfr-Hcp6-(GSSG)2-Hcp1-(AP)10-LolC-C8H-3F	Hcp6-(GSSG)2-Hcp1-(AP)10-LolC-C8H-3F	
	pM264(3'gca)-Hcp6 (coLm)-(GSSG)2-Hcp1 (coLVS)			pM264(5'gca)-LolC (coLVS)	pFNLdA-bfr-Hcp6-(GSSG)2-Hcp1-LolC-C8H-3F	Hcp6-(GSSG)2-Hcp1-LolC-C8H-3F	

Supplementary Table S8 (continued). Electra Reactions linking LolC to Hcp6-(GSSG)2-Hcp1 or Hcp6-(GSSG)2-Hcp2

MOTHER plasmids (SapI sites used for joining ORFs shown below)							
**3'TCT**	**3'GCA**	**5'GCA, 3'TCT**	**5'TCT**	**5'GCA**	**Recombinant Plasmid**	**Fusion Protein**	**Used in a Vaccine**
	pM264(3'gca)-Bp Hcp6 (coLm)-(GSSG)2-Hcp2 (coLVS)	pM264(5'gca,3'tct)-GSAGSAAGSGEF	pM264(5'tct)-LolC (coLVS)		pFNLdA-bfr-Hcp6-(GSSG)2-Hcp2-GSAGSAAGSGEF-LolC-C8H-3F	Hcp6-(GSSG)2-Hcp2-GSAGSAAGSGEF-LolC-C8H-3F	
	pM264(3'gca)-Bp Hcp6 (coLm)-(GSSG)2-Hcp2 (coLVS)	pM264(5'gca,3'tct)-A(EAAAK)3A	pM264(5'tct)-LolC (coLVS)		pFNLdA-bfr-Hcp6-(GSSG)2-Hcp2-A(EAAAK)3A-LolC-C8H-3F	Hcp6-(GSSG)2-Hcp2-A(EAAAK)3A-LolC-C8H-3F	
	pM264(3'gca)-Bp Hcp6 (coLm)-(GSSG)2-Hcp2 (coLVS)	pM264(5'gca,3'tct)-(AP)10	pM264(5'tct)-LolC (coLVS)		pFNLdA-bfr-Hcp6-(GSSG)2-Hcp2-(AP)10-LolC-C8H-3F	Hcp6-(GSSG)2-Hcp2-(AP)10-LolC-C8H-3F	
	pM264(3'gca)-Bp Hcp6 (coLm)-(GSSG)2-Hcp2 (coLVS)			pM264(5'gca)-LolC (coLVS)	pFNLdA-bfr-Hcp6-(GSSG)2-Hcp2-LolC-C8H-3F	Hcp6-(GSSG)2-Hcp2-LolC-C8H-3F	

Supplementary Table S9. Other Plasmids

Plasmid	Description	5’ SapI cloning site	3’ SapI cloning site	Antibiotic Resistance	Reference or Source
pFNL/pbfr-SD-iglA	*E. coli-Francisella* shuttle expression vector expressing *F. tularensis iglA* used to construct Electra compatible DAUGHTER plasmids	NA	NA	Km^r Amp^r	Jia, Q., et al. Vaccine 34, 4969-4978 (2016).
pM264	Electra MOTHER plasmid for cloning ORFs	ATG (Met)	GGT (Gly)	Amp^r	ATUM
pM268	Electra MOTHER plasmid for cloning ORFs	ATG (Met)	GGT (Gly)	Amp^r	ATUM
pRham N-His SUMO Kan	*E. coli* expression vector with N-His SUMO tag and rhamnose inducible expression	NA	NA	Km^r	Lucigen
pRham-H6-SUMO-Gly-Ser-Bp hcp1	Overexpression of H6-SUMO-Gly-Ser-Hcp1	NA	NA	Km^r	This study
pRham-H6-SUMO-Bp hcp2	Overexpression of H6-SUMO-Hcp2	NA	NA	Km^r	This study
pRham-H6-SUMO-Gly-Ser-Bp hcp6	Overexpression of H6-SUMO-Gly-Ser-Hcp6	NA	NA	Km^r	This study
pRham-H6-SUMO-Bp lolC 51-273	Overexpression of H6-SUMO-LolC	NA	NA	Km^r	This study

NA, not applicable.

Supplementary Table S10. Fluorescent antibodies for flow cytometry

Antibody	Company	Catalog Number	Clone
R718 Rat Anti-Mouse CD8a	BD Biosciences	566985	53-6.7
Alexa Fluor® 700 anti-mouse CD8a Antibody	BioLegend	100730	53-6.7
BUV395 Rat Anti-Mouse CD3 molecular complex (CD3e)	BD Biosciences	740268	17A2
Brilliant Violet 650™ anti-mouse IFN-γ	BioLegend	505832	XMG1.2
Brilliant Violet 785™ anti-mouse TNF-α	BioLegend	506341	MP6-XT22
Alexa Fluor® 488 anti-mouse IL-17A	BioLegend	506910	TC11-18H10.1
Brilliant Violet 421™ anti-mouse IL-2 Antibody	BioLegend	503826	JES6-5H4
PE anti-mouse Perforin	BioLegend	154406	S16009B
Alexa Fluor® 647 anti-mouse CD4	BioLegend	100530	RM4-5
Granzyme B Monoclonal Antibody (NGZB), PE-Cyanine7	Thermo Scientific	25-8898-82	NGZB

Supplementary Table S11. Final Survival and Survival with Sterile Immunity

			Protective Efficacy Experiment	1		2		3		4		5a		5b		5c		6	
			Bp Challenge Dose (CFU)	2,225		1,800		2,700		1,430		1,520		1,890		2,290		7,800	
			Immunization-Challenge Interval (weeks)	4		4		4		12		6		6		6		6	
Vaccine	Immunization Route	Immunization Dose (CFU)	Number of Doses	Final Survival (%)	Survival with Sterile Immunity (%)	Final Survival (%)	Survival with Sterile Immunity (%)	Final Survival (%)	Survival with Sterile Immunity (%)	Final Survival (%)	Survival with Sterile Immunity (%)	Final Survival (%)	Survival with Sterile Immunity (%)	Final Survival (%)	Survival with Sterile Immunity (%)	Final Survival (%)	Survival with Sterile Immunity (%)	Final Survival (%)	Survival with Sterile Immunity (%)
Negative Control (Sham)				0	0	0	0	0	0	0	0	31	19	6	6	0	0	0	0
Positive Control (Bp82)	ID	1.00E+06	3	75	50	100	50	25	0	38	0	56	13	33	27	31	25	31	6
Parental Vector (LVS Δ*capB*)	ID	1.00E+06	3	0	0														
	ID	2.00E+06	3			38	25	0	0	63	63	44	31	20	13	0	0		
	IN	2.00E+06	3					100	86	100	67								
	IN	2.00E+06	2					88	13										
	IN	2.00E+06	1					63	38										
rLVS Δ*capB*/Bp Hcp6-Hcp1	ID	1.00E+06	3	88	75	25	25												
	ID	2.00E+06	3			63	63												
	ID	4.00E+06	3			63	25												
	ID	8.00E+06	3			63	25												
rLVS Δ*capB*/Bp Hcp6-Hcp2	ID	1.00E+06	3	88	50														
	ID	2.00E+06	3			75	50												
	ID	4.00E+06	3			75	25												
rLVS Δ*capB*/Bp LolC-Hcp1	ID	2.00E+06	3	38	25														
rLVS Δ*capB*/Bp LolC-Hcp2	ID	2.00E+06	3	38	38														
rLVS Δ*capB*/Bp Hcp6-Hcp1-LolC	ID	2.00E+06	3			75	38	13	13	25	13								
	IN	2.00E+06	3					75	13	75	63								
	IN	2.00E+06	2					86	14										
	IN	2.00E+06	1					88	0										
rLVS Δ*capB*/Bp Hcp6-Hcp1-Hcp2	ID	2.00E+06	3			88	38	0	0	57	43	75	56	13	13	0	0	6	0
	IN	2.00E+06	3					88	0	50	50							44	25
	IN	2.00E+06	2					100	0										
	IN	2.00E+06	1					86	0										
rLVS Δ*capB*/Bp LolC-Hcp6-Hcp1-Hcp2	ID	2.00E+06	3			50	25												

Supplementary Table S12. Candidate melioidosis vaccines with substantial efficacy against lethal respiratory challenge in the BALB/c mouse model

Vaccine	Immunization Route	Immunization-Challenge Interval	Challenge Route	Bp Challenge Strain	Challenge Dose CFU (# of LD50s)	Survival (%)	Survival Endpoint (days)	Reference
rLVS ΔcapB/Bp Hcp6-Hcp1-LolC	ID	4 weeks	IN	1026b	1,800 (4x)	75	42	This study (Experiment 2)
	IN	4 weeks	IN	1026b	2,700 (6x)	75 to 88	42	This study (Experiment 3)
	ID	12 weeks	IN	1026b	1,430 (3.2x)	25	42	This study (Experiment 4)
	IN	12 weeks	IN	1026b	1,430 (3.2x)	75	42	This study (Experiment 4)
rLVS ΔcapB/Bp Hcp6-Hcp1-Hcp2	ID	4 weeks	IN	1026b	1,800 (4x)	88	42	This study (Experiment 2)
	IN	4 weeks	IN	1026b	2,700 (6x)	86 to 100	42	This study (Experiment 3)
	ID	12 weeks	IN	1026b	1,430 (3.2x)	57	42	This study (Experiment 4)
	IN	12 weeks	IN	1026b	1,430 (3.2x)	50	42	This study (Experiment 4)
	ID	6 weeks	IN	1026b	1,520 (1.3x)	75	42	This study (Experiment 5a)
	ID	6 weeks	IN	1026b	1,890 (1.6x)	13	42	This study (Experiment 5b)
	ID	6 weeks	IN	1026b	2,290 (1.9x)	0	42	This study (Experiment 5c)
	ID	6 weeks	IN	1026b	7,800 (2.8x)	6	42	This study (Experiment 6)
	IN	6 weeks	IN	1026b	7,800 (2.8x)	44	42	This study (Experiment 6)
Bp82 (Bp 1026b Δ*purM*)	SC	Not stated	IN	1026b	5,000 (~5x)	60	60	[1]
	ID	4, 6, or 12 weeks	IN	1026b	(1.3 to 6x)	25 to 100	42	This study (Experiments 1-6)
B. mallei batA	Intratracheal (IT)	30 to 45 days	IT	1026b	(~5x)	67	55	[2]
				K96243	(~5x)	85	55	
Heat-inactivated Bp in CLDC adjuvant	IN	2 weeks	IN	1026b	7,500 (8×)	100	40	[3]
B. mallei protein BopA in CLDC adjuvant	IN	2 weeks	IN	1026b	(2x)	60	55	[4]
Bp OMVs	SC	30 days	Aerosol	1026b	5,350 (5x)	53	14	[5]
	IN	30 days	Aerosol	1026b	5,350 (5x)	13	14	
Parainfluenza Virus 5 expressing *B. mallei* BatA (PIV5-BatA)	IN	6 weeks	IT	K96243	300 (5x)	60	35	[6]

1. Silva, E.B., et al., *Correlates of immune protection following cutaneous immunization with an attenuated Burkholderia pseudomallei vaccine.* Infect Immun, 2013. **81**(12): p. 4626-34.
2. Zimmerman, S.M., et al., *Antibodies against In Vivo-Expressed Antigens Are Sufficient To Protect against Lethal Aerosol Infection with Burkholderia mallei and Burkholderia pseudomallei.* Infect Immun, 2017. **85**(8).

3. Henderson, A., et al., *Mucosal immunization with liposome-nucleic acid adjuvants generates effective humoral and cellular immunity.* Vaccine, 2011. **29**(32): p. 5304-12.

4. Whitlock, G.C., et al., *Protective response to subunit vaccination against intranasal Burkholderia mallei and B. pseudomallei challenge.* Procedia Vaccinol, 2010. **2**(1).

5. Nieves, W., et al., *A naturally derived outer-membrane vesicle vaccine protects against lethal pulmonary Burkholderia pseudomallei infection.* Vaccine, 2011. **29**(46): p. 8381-9.

6. Lafontaine, E.R., et al., *The autotransporter protein BatA is a protective antigen against lethal aerosol infection with Burkholderia mallei and Burkholderia pseudomallei.* Vaccine X, 2019. **1**: p. 100002.

Supplementary Table S13. Candidate melioidosis vaccines with substantial efficacy against lethal respiratory challenge in the C57BL/6 mouse model

Vaccine	Immunization Route	Immunization-Challenge Interval	Challenge Route	Bp Challenge Strain	Challenge Dose CFU (# of LD50s)	Survival (%)	Survival Endpoint (days)	Reference
Bp82	SC	Not stated	IN	1026b	12,000 (~5x)	100	60	[1]
B. mallei batA	Intratracheal (IT)	30 days	IT	1026b	(4x)	100	35	[2]
Bp OMVs	SC	1 month	Aerosol	K96243	1,500 (8x)	100	30	[3]
CPS-CRM197 + Hcp1	SC	5 weeks	Aerosol	K96243	~1,600 (~10x)	100	35	[4]
CPS-CRM197 + Hcp1	SC	~1 month	Aerosol	K96243	2,450 (~6x)	60	30	[5]
				K96243	2,450 (~6x)	20	70	
	SC	~1 month	Aerosol	K96243	1,570 (~4x)	80	30	
				K96243	1,570 (~4x)	70	86	
CPS-CRM197 + Hcp1 + AhpC^{C57G}	SC	~1 month	Aerosol	K96243	2,860 (~7x)	60	30	
				K96243	2,860 (~7x)	60	82	
	SC	~1 month	Aerosol	K96243	2,450 (~6x)	56	30	
				K96243	2,450 (~6x)	22	70	
	SC	~1 month	Aerosol	K96243	1,570 (~4x)	80	30	
				K96243	1,570 (~4x)	40	86	
CPS-CRM197 + Hcp1	SC	~5.5 weeks	Aerosol	K96243	1,350 (3.4x)	80	60	[6]
	SC	~5.5 weeks	Aerosol	MSHR5855	(11x and 12x)	35	60	
CPS-CRM197 + Hcp1 + AhpC^{C57G}	SC	~5.5 weeks	Aerosol	K96243	1,350 (3.4x)	50	60	
CPS-CRM197 + AhpC^{C57G}	SC	5 weeks	Aerosol	K96243	4,040 (27x) or 4,180 (28x)	70	35	[7]
AuNP-FlgL-LPS	SC	3 weeks	IN	K96243	1,060 (3.4x)	90	35	[8]
AuNP-combo-LPS	SC	3 weeks	IN	K96243	1,060 (3.4x)	100	35	
AuNP-OpcP-LPS	IN	3 weeks	IN	K96243	90,000 (6x)	90	35	[9]
AuNP-Combo2-LPS	IN	3 weeks	IN	K96243	75,000 (5x)	100	35	

1. Silva, E.B., et al., *Correlates of immune protection following cutaneous immunization with an attenuated Burkholderia pseudomallei vaccine.* Infect Immun, 2013. **81**(12): p. 4626-34.

2. Zimmerman, S.M., et al., *Antibodies against In Vivo-Expressed Antigens Are Sufficient To Protect against Lethal Aerosol Infection with Burkholderia mallei and Burkholderia pseudomallei.* Infect Immun, 2017. **85**(8).

3. Baker, S.M., et al., *Burkholderia pseudomallei OMVs derived from infection mimicking conditions elicit similar protection to a live-attenuated vaccine.* NPJ Vaccines, 2021. **6**(1): p. 18.

4. Burtnick, M.N., et al., *Development of Subunit Vaccines That Provide High-Level Protection and Sterilizing Immunity against Acute Inhalational Melioidosis.* Infect Immun, 2018. **86**(1).

5. Klimko, C.P., et al., *Layered and integrated medical countermeasures against Burkholderia pseudomallei infections in C57BL/6 mice.* Front Microbiol, 2022. **13**: p. 965572.

6. Biryukov, S.S., et al., *Evaluation of two different vaccine platforms for immunization against melioidosis and glanders.* Front Microbiol, 2022. **13**: p. 965518.

7. Schmidt, L.K., et al., *Development of Melioidosis Subunit Vaccines Using an Enzymatically Inactive Burkholderia pseudomallei AhpC.* Infect Immun, 2022. **90**(8): p. e0022222.

8. Muruato, L.A., et al., *Use of Reverse Vaccinology in the Design and Construction of Nanoglycoconjugate Vaccines against Burkholderia pseudomallei.* Clin Vaccine Immunol, 2017. **24**(11).

9. Tapia, D., et al., *Multicomponent Gold-Linked Glycoconjugate Vaccine Elicits Antigen-Specific Humoral and Mixed T(H)1-T(H)17 Immunity, Correlated with Increased Protection against Burkholderia pseudomallei.* mBio, 2021. **12**(3): p. e0122721.
